# Upregulation of p53 through induction of MDM2 degradation: improved potency through the introduction of an alkylketone sidechain on the anthraquinone core

**DOI:** 10.1080/14756366.2022.2116699

**Published:** 2022-08-31

**Authors:** Ravi Tripathi, Abiodun Anifowose, Wen Lu, Xiaoxiao Yang, Binghe Wang

**Affiliations:** Department of Chemistry, Center for Diagnostics and Therapeutics, Georgia State University, Atlanta, GA, USA

**Keywords:** p53, MDM2, anthraquinone, cancer, structure–activity relationship (SAR)

## Abstract

Overexpression of ubiquitin ligase MDM2 causes depletion of the p53 tumour-suppressor and thus leads to cancer progression. In recent years, anthraquinone analogs have received significant attention due to their ability to downregulate MDM2, thereby promoting p53-induced apoptosis. Previously, we have developed potent anthraquinone compounds having the ability to upregulate p53 *via* inhibition of MDM2 in both cell culture and animal models of acute lymphocytic leukaemia. Earlier work was focussed on mechanistic work, pharmacological validation of this class of compounds in animal models, and mapping out structural space that allows for further modification and optimisation. Herein, we describe our work in optimising the substituents on the two phenol hydroxyl groups. It was found that the introduction of an alkylketone moiety led to a potent series of analogs with **BW-AQ-350** being the most potent compound yet (IC_50_ = 0.19 ± 0.01 µM) which exerts cytotoxicity by inducing MDM2 degradation and p53 upregulation.

## 1. Introduction

Acute lymphocytic leukaemia (ALL) is the most prevalent cancer among children and adolescents worldwide^1^. ALL cells are immature malignant lymphoblasts with unsuppressed proliferation ability. If untreated, ALL normally leads to death within a few months. One well-characterized genomic factor is the ubiquitously adoption of a malfunctioned p53/MDM2 signalling pathway^2–5^. While p53 is a tumour suppressor, controlling apoptosis and thus serving as a brake in cellular growth and replication^4^, MDM2 is an E3 ligase that targets p53 to proteolysis^5^^,^^6^. In normal cells, there is a well-maintained feedback control of the p53-MDM2 axis. However, in cancerous cells, the function of p53 is commonly inactivated by either mutation to the TP53 gene or through overexpression of MDM2, leading to the progression of cancer^7^. In some leukaemia phenotypes, both p53 mutation and MDM2 overexpression can be found, which correlates with their aggressive malignancy^8^^,^^9^. In ALL patients, about 30% have over-expression of MDM2, leading to unrestrained proliferation^10^^,^^11^. From a therapeutic point of view, overexpression of MDM2 has also been implicated in chemoresistance and relapse after treatment^5^^,^^10^^,^^12^. Restoring p53 function through intercepting the p53-MDM2 axis by inducing MDM2 degradation or inhibiting its E3 ligase activity has proven to be an effective therapeutic approach for ALL^10^^,^^11^^,^^13^^,^^14^. For example, nutlins^10^^,^^15^^,^^16^, MK-8242^17^, RG7112^18^, and some stapled peptides^19^^,^^20^ bind to the p53 binding domain of MDM2 and thereby prevent ubiquitination of p53. Further, a small molecule Nilotinib downregulates MDM2 by promoting its self-ubiquitination^21^. Another small molecule, triptolide inhibits mRNA expression of MDM2 in cancer cells^22^. PROTAC molecules that target p53-MDM2 have been reported to downregulate MDM2 through recruiting other E3 ligase^23^ or MDM2^24^ itself to induce polyubiquitination of MDM2 and the subsequent proteasomal degradation.

**Figure 1.**
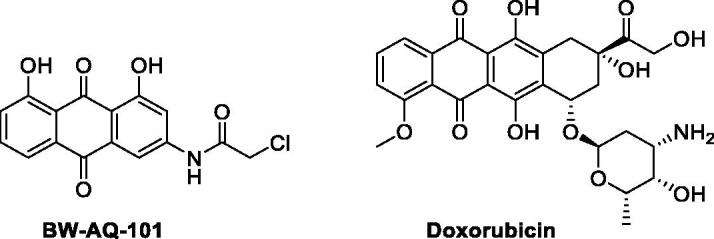
Chemical structures of **BW-AQ-101** and doxorubicin.

In targeting MDM2 for degradation, we discovered a series of anthraquinones with anticancer activity^18^^,^^25–27^. Previous studies on the mechanism of action showed the ability of the lead compound **BW-AQ-101** (Figure 1) to bind to the RING domain of MDM2, thus disrupting the interaction between MDM2 and MDM4 and preventing the formation of the MDM2/MDM4 heterodimer complex which can stabilise MDM2^28^. As a result, **BW-AQ-101** induces self-ubiquitination and proteasomal degradation of MDM2, leading to the upregulation of p53 and apoptosis^28^. In EU-1 ALL cells (an in-house WT-p53 ALL cell line separated from a paediatric ALL patients), **BW-AQ-101** showed an IC_50_ of about 0.8 μM. *In-vivo* studies in mouse ALL models with EU-1 cell xenograft have demonstrated pharmacological efficacy of **BW-AQ-101**. Specifically, treatment with **BW-AQ-101** (20 mg/kg/day, i.v. 3 times per week) led to complete remission without relapse for 150 days, the duration of the study. In comparison, mice in the vehicle control group died within 45 days. Further, mechanistic studies using the p53-null EU-8 cell line also support the role of p53 in the mechanism of this class of compounds. With success in demonstrating the pharmacological efficacy of this class of compounds and a detailed understanding of the mechanism of action, we systematically studied the SAR of **BW-AQ-101** analogs^18^^,^^26^ and identified **BW-AQ-238**^25^ as a compound with improved water solubility while maintaining similar cytotoxicity (Table 1, Entry 1). Water solubility was further improved substantially by making amino acid prodrugs of **BW-AQ-238** without compromising its *in-vitro* activity^25^. Herein, we describe our continuous efforts in optimising this class of compounds seeking improved potency.

**Table 1.** Analogs design.
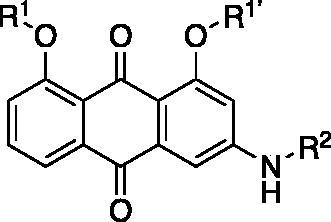

Entry	BW-AQ-#	R^1^/R^1′^	R^2^	IC_50_ (µM) in EU-1 cells
1	**238**	R^1^=R^1′^= 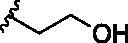	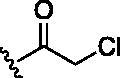	0.74 ± 0.12
2	**260**	R^1^=R^1′^= 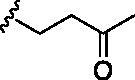	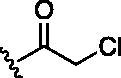	0.45 ± 0.02
3	**295**	R^1^=R^1′^= 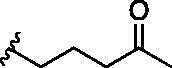	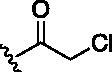	0.43 ± 0.06
4	**345**	R^1^=R^1′^= 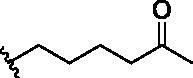	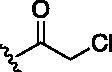	0.79 ± 0.03
5	**336**	R^1^=H, R^1′^= 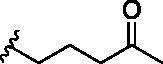	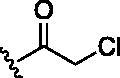	0.84 ± 0.02
6	**350**	R^1^=R^1′^= 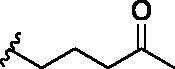	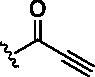	0.19 ± 0.01
7	**353**	R^1^=R^1′^= 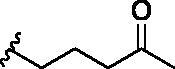	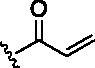	6.21 ± 0.45
8	**354**	R^1^=R^1′^= 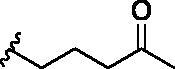	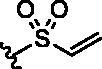	3.45 ± 0.45
9	**349**	R^1^=R^1′^= 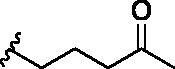	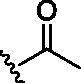	>100
	Control compounds			
10	Propiolamide (CHCCONH_2_)			33.5 ± 7.8
11	Doxorubicin			0.15 ± 0.02

## 2. Results and discussion

Anthraquinone derivatives, such as doxorubicin and mitoxantrone analogs are potent anticancer drugs owing to their ability to intercalate DNA to induce damage^29^. However, our earlier studies^28^ have shown the ability of our lead compound **BW-AQ-101** (Figure 1) derived from rhein to down-regulated MDM2 without the general cytotoxicity by inducing DNA damage. Moreover**, BW-AQ-101** did not show cardiotoxicity at the effective dosage in animal models^28^ as usually seen with doxorubicin^30^ and mitoxantrone^31^. It has been reported that the cardiotoxicity of doxorubicin is attributed to the redox activity of the anthracycline core^32–34^. The lack of cardiotoxicity of our lead compounds is consistent with the significant difference in the cyclo-voltammetry data between an anthraquinone core and doxorubicin^35^^,^^36^. Such differences are easy to understand because of the lack of a fused dihydroquinone ring to the quinone core in our analogs when compared with the presence of such a fused structure in doxorubicin as shown in Figure 1. Henceforth, on understanding the importance of 1,8-hydroxyl or 1,8-alkoxyl substituted anthraquinone core for MDM2 downregulation, we decided to conduct further SAR studies by modifying the R^1^/R^1′^ and R^2^ positions while keeping the anthraquinone core intact. In this study, we optimised this class of compounds by introducing an aliphatic ketone group at the R^1^/R^1′^ positions. This decision was made for two reasons: our understanding of the sidechain’s ability to tolerate modification and our plan to introduce a polar hydrogen bond acceptor group. Of note, an aliphatic ketone group is present in several FDA-approved drugs, such as nabumetone^37^, methadone^38^, and warfarin^37^.

### 2.1. Chemistry

**Scheme 1.**
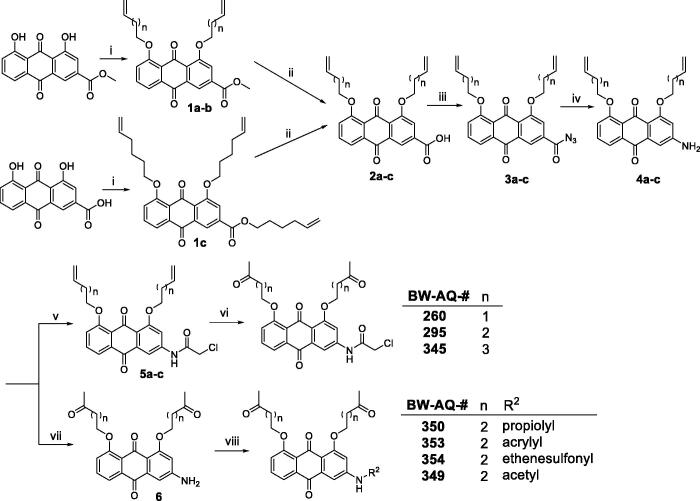
Synthesis of the AQ analogs. (i) Bromo-1-alkene, K_2_CO_3_, DMF, 100–110 °C, 5 h; (ii) LiOH, H_2_O, THF, 4 h; (iii) DPPA, Et_3_N, DMF, rt, 30 min; (iv) (a) dioxane, reflux, 2 h; (b) H_2_O, 1 h, 50 °C; (v) chloroacetyl chloride, 1,4-dioxane, rt, 15 min; (vi) PdCl_2_, CuCl, O_2_, DMF, H_2_O, rt, overnight; (vii) PdCl_2_, CuCl, O_2_, DMF, H_2_O, rt, overnight; (viii) **BW-AQ-350**: propiolic acid, EDC, DMAP, DCM; **BW-AQ-353**: acryloyl chloride, 1,4-dioxane, Et_3_N, rt, 15 min; **BW-AQ-349**: acetyl chloride, 1,4-dioxane, Et_3_N, rt, 15 min; **BW-AQ-354**: (a) 2-chloro-1-ethanesulfonyl chloride, Et_3_N, 1,4-dioxane, rt, 15 min; (b) TBAF, THF, rt, 3 h.

The synthesis of the designed compounds (Table 1) employed a similar approach as that of the previously published analogs^18^^,^^26^. Specifically, the synthesis started with either rhein or its methyl ester (Scheme 1). Alkylation with the corresponding alkenyl halide at the phenol hydroxyl groups of the anthraquinone core allowed for the introduction of the alkenyl group as a latent ketone moiety. Then the ester group of intermediates **1** was hydrolysed before introducing an azido group on the free carboxylic acid **2**, setting up a Curtis rearrangement for the subsequent formation of the arylamino group on **4**. The introduction of various acyl or sulphonyl groups led to the installation of different R^2^ in the target compounds. The conversion of the alkene moiety to a ketone group was conducted as the last step through Wacker oxidation for the synthesis of compounds with the chloroacetyl moiety (**BW-AQ-260, -295, -345**). However, for analogs with functional groups that are sensitive to Wacker oxidation, including **BW-AQ-350**, **-353**, and **-354**, the oxidative conversion of the alkenyl moiety to a ketone group was conducted as the penultimate step.

**Scheme 2.**
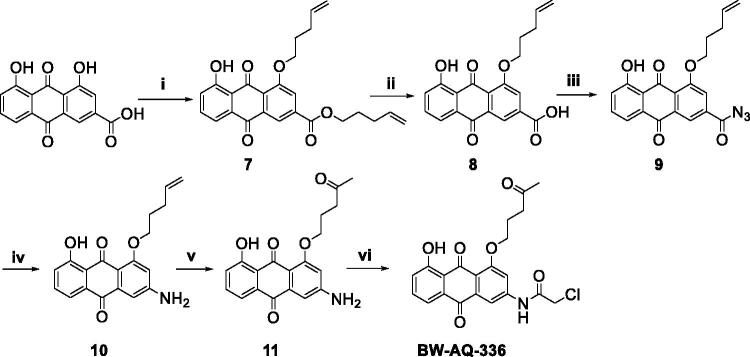
Synthesis of the **BW-AQ-336** analog. (i) 5-bromo-1-pentene, K_2_CO_3_, DMF, 90–100 °C, 4 h; (ii) LiOH, H_2_O, THF, 4 h; (iii) DPPA, Et_3_N, DMF, rt, 30 min; (iv) (a) dioxane, reflux, 2 h; (b) H_2_O, 1 h, 50 °C; (v) PdCl_2_, CuCl, O_2_, DMF, H_2_O, rt, overnight; (vi) chloroacetyl chloride, 1,4-dioxane, rt, 15 min.

**Figure 2.**
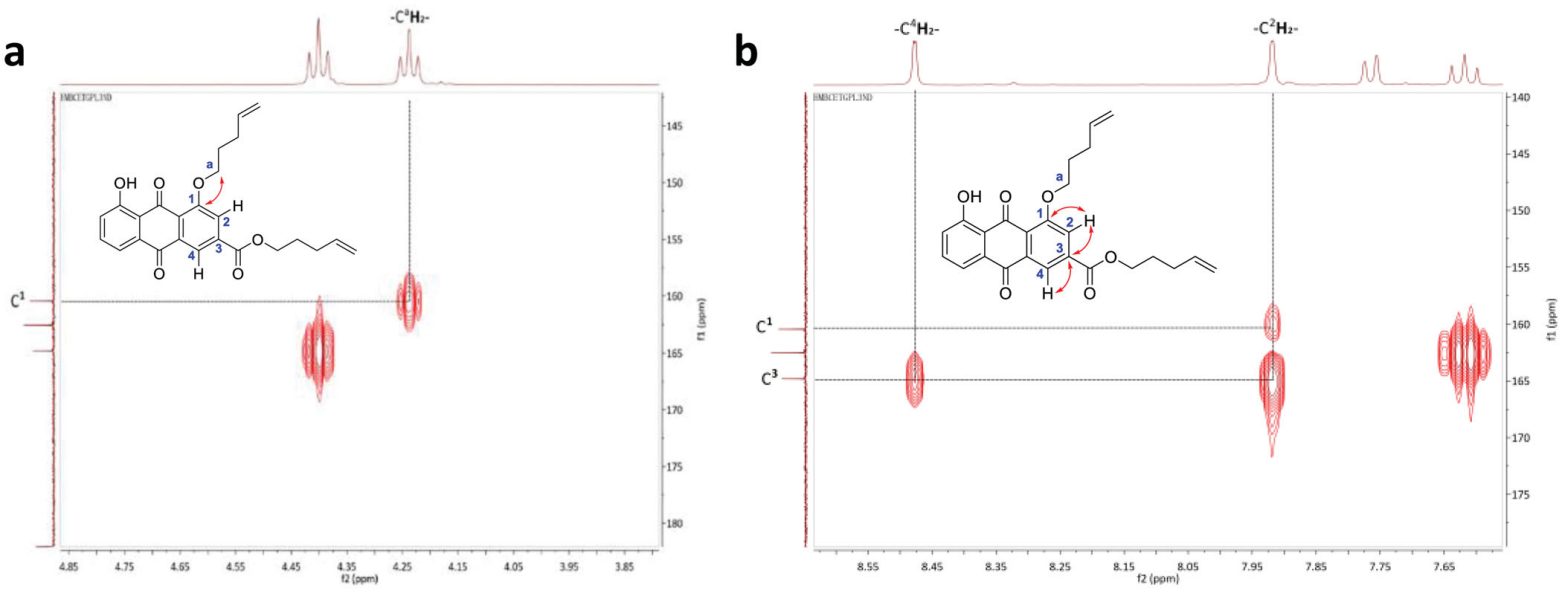
Detailed correlation for structure determination of **BW-AQ-336** by HMBC 2 D-NMR.

In addition to the dialkylated analogs, a mono-substituted anthraquinone analog (**BW-AQ-336**) was synthesised by carefully controlling the alkylation conditions. This compound allows for probing the effect of mono-substitution *vs.* di-substitution of the hydroxyl groups present on the anthraquinone core (Scheme 2). Using HMBC 2 D-NMR (Figure 2, Figure S4), it was found the methylene proton of the alkenyl side-chain correlated with the aromatic carbon at position 2, which can be validated by the net correlation shown in Figure 2. Thus, the alkenyl substitution position was confirmed to be at the R^1′^ position. We reasoned that the preferential formation of this regioisomer could potentially be due to the presence of an electron-withdrawing carboxyl group on the right phenyl ring of the anthraquinone moiety, which makes the hydroxyl group at the R^1′^ position (general structure in Table 1) slightly more acidic than the one on the left side and thus easier to deprotonate.

### 2.2. *In-vitro* structure-activity relationship (SAR) studies

#### 2.2.1. Modification at the R^1^/R^1′^ position

In designing our new analogs, we considered prior SAR information in our earlier studies^18^^,^^26^. Specifically, the anthraquinone core was found to be essential for activity and the R^1^/R^1′^ positions were found to tolerate some degree of variations. We also found an ethyl or a hydroxyethyl (**BW-AQ-238**) (Table 1, Entry 1) substitution at the R^1^/R^1′^ position allowed for the retention of their ability to lead to MDM2 degradation and p53 activation. At the R^2^ position, a chloroacetyl group was found to be important to activity in downregulating MDM2 in the ALL cells^18^. Bearing these SAR findings in mind, we initially focussed on modifying the side-chain (R^1^/R^1′^) positions while keeping R^2^ as the chloroacetyl group (Table 1, Entries 2–5). We were especially interested in incorporating a ketone group as a hydrogen-bond acceptor. To explore the SAR of the chain length, we studied C4 to C6 in the chain length. Table 1 shows the analogs designed.

**Figure 3.**
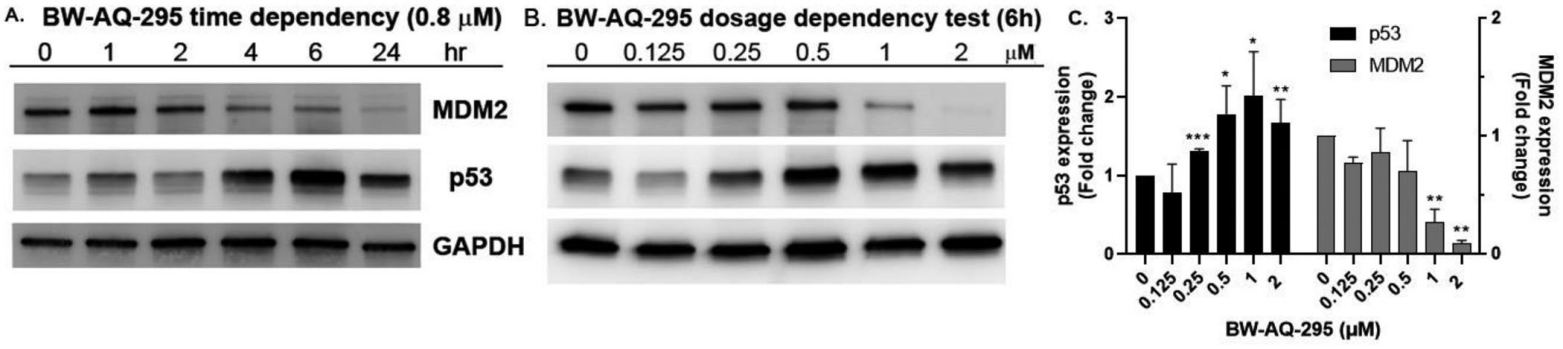
Western blot showed the downregulation of MDM2 and upregulation p53 by **BW-AQ-295** in time- (A) and dosage-dependent fashions (B) in EU-1 leukaemia cells. GAPDH was probed as the loading control. (C) Relative quantification analysis of the dose-dependency of the Western-blot results of **BW-AQ-295** (*n* = 3, mean ± *SD*, data is shown as fold changes compared to the vehicle control group after normalisation by GAPDH, *p < 0.05, **p < 0.01, ***p < 0.001).

With the installation of alkyl ketone groups at the R^1^ and R^1′^ positions, we evaluated the cytotoxicity of these analogs in EU-1 cells using **BW-AQ-238** as the positive control. It was found that the C4 and C5 ketone derivatives **BW-AQ-260** and **BW-AQ-295** were about 2-fold more potent than **BW-AQ-238** (Table 1). Further increasing the chain length to C6 (**BW-AQ-345**) increased the IC_50_ by about 1-fold compared to **BW-AQ-295**. Further, treatment of EU-1 cells with **BW-AQ-336** (mono pentan-2-one substitution) resulted in a significant decrease in potency in comparison to **BW-AQ-295**. Such results indicate that the substitution of both phenol hydroxyl groups is of benefit for preserving the potency. With **BW-AQ-295** being the most potent, we conducted experiments to confirm its degradation of MDM2 using EU-1 cells. Western-blot studies were performed accordingly (Figure 3 and Figure S2). The initial time dependency experiment showed 0.8 µM **BW-AQ-295** decreased MDM2 level after 4 h incubation and the effect of increasing p53 level was the most pronounced at the 6 h time point. Therefore, the dose-response assessment was conducted at the 6 h time point. A significant increase in p53 expression level was seen at concentrations higher than 0.25 µM, reaching a maximum at 1 µM (Figure 3(C)). Correspondingly, a significant decrease in the MDM2 level was also seen at 1 µM. At 2 μM concentration, **BW-AQ-295** decreased MDM2 to an almost undetectable level. Such results are consistent with that of earlier analogs (e.g. **BW-AQ-101**), with which we had conducted extensive studies in pharmacological validation and mechanistic understanding^28^.

#### 2.2.2. Modification at the R^2^ position

Our previous SAR studies^18^^,^^26^ suggest some essential features at the R^2^ position. Replacement of the chloroacetyl group with other alkylating moieties, such as bromoacetyl does not significantly improve potency, while substitution with the azidoacetyl group retains the activity^18^. In addition, modification of the chloroacetamide group to chloromethyl carbamate or hydroxyacetamide led to the loss of cytotoxicity^26^. Further, introducing steric hindrance to the alkylating moiety through α-methylation resulted in decreased activity^26^. Therefore, having **BW-AQ-295** with the introduction of terminal alkyl ketone groups at the R^1^ and R^1′^ positions, we further studied if the chloroacetamide group of **BW-AQ-295** (R^2^ substitution) can be replaced by other commonly used electrophiles^39–43^ including acrylamide^44^^,^^45^, propynamide^46^^,^^47^, and ethensulfonamide^48^ (Table 1). Compared to **BW-AQ-295**, the acrylamide analog **BW-AQ-353** was about 15-fold less potent. A similar trend was also seen in our previous studies^18^, in which the acrylamide analog showed an ∼20-fold decrease in cytotoxic activity. The sulphonamide analog **BW-AQ-354** was found to be more potent than **BW-AQ-353**. To our delight, the propynamide analog **BW-AQ-350** showed a 2-fold increase in potency compared to **BW-AQ-295**, to a level that is comparable to that of doxorubicin (Table 1, Entry 11). The lack of activity in the acetamide analog **BW-AQ-349** (Table 1, Entry 9) and the propiolamide analog (Table 1, Entry 10) suggests a role for the electrophilic moiety and the anthraquinone core. At least, such results suggest the need to explore the effect of their chemical reactivity as an electrophile on their cytotoxicity.

Under physiological conditions, the electrophilic moiety of these anthraquinone analogs is capable of reacting with intracellular nucleophiles, such as a protein thiol group to various degrees^49^. Therefore, we investigated using HPLC the general reactivity of our AQ-analogs (Table 1, Entries 3, 6–9) towards a commonly used model thiol, *N*-acetyl cysteine (NAC)^50^. A comparative analysis of the pseudo-first-order kinetics of the AQ-analogs with NAC at 37 °C in phosphate-buffered saline (PBS) solution indicated the order of reactivity being **BW-AQ-350 **>** BW-AQ-353 **>** BW-AQ-295 **≈** BW-AQ-354 ≫ BW-AQ-349** (propynamide >  acrylamide > chloroacetamide ≈ sulphonamide ≫ acetamide) (Figure S5). **BW-AQ-350** showed the highest reactivity towards NAC among the tested analogs. As expected, **BW-AQ-349** remained unchanged during the study duration (>400 min) because of its lack of an electrophilic group. Our results are in accordance with literature precedence showing a faster thiol reaction with the Michael acceptor with an alkynyl moiety than that with an alkenyl electrophile^49^^,^^51^. Interestingly, the reactivity of the electrophilic moiety is not in the same order as the observed cytotoxicity. For example, **BW-AQ-353** was found to be more reactive to NAC than **BW-AQ-295** and yet showed much lower potency. Therefore, the chemical reactivity of the electrophile does not seem to be the determining factor for potency. This is understandable since we have no information to indicate that an electrophile is an absolute requirement for activity. Further, even if a reaction involving an electrophile is involved, there are other factors that help shape both potency and selectivity^52^^,^^53^. Otherwise, we would be simply dealing with general cytotoxicity. One will need much more work including extensive structural studies to truly assess the contribution of each factor.

**Figure 4.**
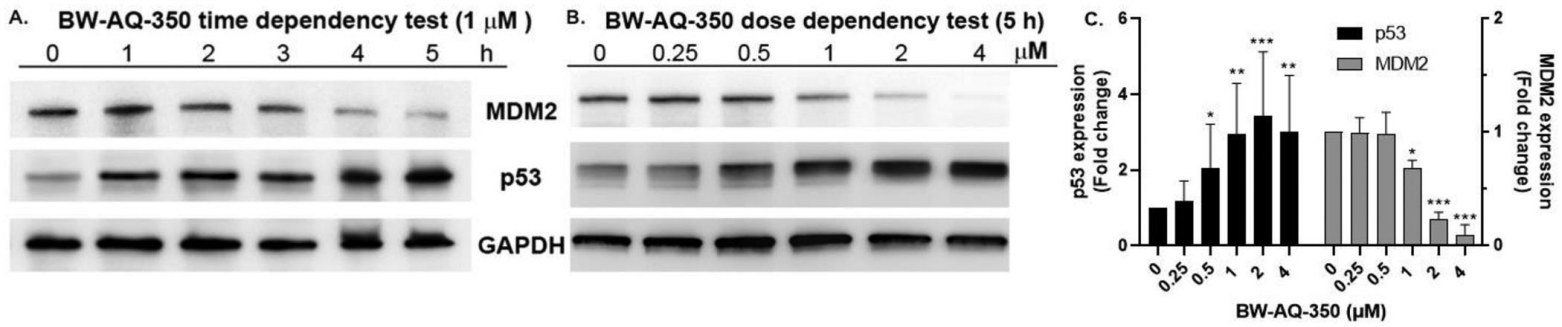
Western blot showed the downregulation of MDM2 and upregulation p53 by **BW-AQ-350** in time- (A) and dosage-dependent fashion (B) in EU-1 leukaemia cells. GAPDH was probed as the loading control. (C) Relative quantification analysis of the dose-dependency of the Western-blot results of **BW-AQ-350** (*n* = 3, mean ± *SD*, data is shown as fold changes compared to the vehicle control group after normalisation by GAPDH, *p < 0.05, **p < 0.01, ***p < 0.001).

With its high potency, the propynamide analog **BW-AQ-350** was selected as a representative to test its effect on downregulating MDM2 by Western-blot studies in EU-1 cells. Initial time-dependency studies showed a decreased MDM2 levels after 4 h incubation (Figure S3). Further dose-dependency studies at 5 h showed significantly decreased MDM2 levels at concentrations higher than 1 µM. A dose-dependent increase in p53 expression levels was also observed at concentrations higher than 0.5 µM (Figure 4). To this end, **BW-AQ-350** was found to be the most potent candidate in inhibiting the proliferation of EU-1 cells by downregulating MDM2, thus upregulating the p53 level. With **BW-AQ-295** and **BW-AQ-350** in hand, we sought to test their cytotoxicity in other cell lines to study their general activity. Table 2 shows the cytotoxicity of **BW-AQ-295** and **BW-AQ-350** in cell lines harbouring WT-p53, including MCF7^54^, RS4;11^55^, as well as HeLa cells with aberrant-p53^56^. A non-cancerous embryonic rat cardiomyoblasts cell line H9c2^57^^,^^58^ and human embryonic kidney cell line HEK-293^59^ were also included. These two compounds retained their high potency against RS4;11 ALL cells which further support their cytotoxicity against leukaemia cells harbouring WT-p53 (Table 2, Entry 1). Further, in the cancerous epithelial cells, **BW-AQ-295** showed over 2-fold less potency in HeLa cells (Table 2, Entry 3) when compared to MCF7 cells (Table 2, Entry 2), indicating the importance of WT-p53 for the higher cytotoxicity of **BW-AQ-295**. These results are in accordance with the previous results of analogs of **BW-AQ-101**^18^^,^^28^ In both HeLa and MCF7 cancer cell lines, **BW-AQ-350** showed significantly lower potency than **BW-AQ-295**. This trend can also be seen in HEK-293 cells, though to a less extent (Table 2, Entry 5). In embryonic H9c2 cells, **BW-AQ-350** showed much higher potency than **BW-AQ-295** (Table 2, Entry 4), similar to the trend seen in EU-1 cells. The difference in the order of potency of these two compounds shown in different cell lines warrants further in-depth mechanistic studies. However, we understand the need to exercise caution in interpreting results from different cell lines, especially in inferring mechanistic implications. This is because of the widely observed differences in potency for most anticancer compounds among different cell lines, including those that are general toxins, such as nitrogen mustards^60–62^. Detailed mechanistic studies need to use cell lines with carefully controlled genetic phenotypes, which are beyond the scope of this optimisation effort. Our earlier studies using **BW-AQ-101** provide more information on the pathway implications of this class of compounds^28^. Furthermore, the IC_50_ in the micromolar range indicates aside from activation of p53 through MDM2 down-regulation, **BW-AQ-295** and **BW-AQ-350** may also induce cell death through other p53-independent pathways.

**Table 2.** Cytotoxicity in different cell lines.

Entry	Cell lines	IC_50_ (μM)	
		BW-AQ-295	BW-AQ-350
1	RS4;11	0.69 ± 0.10	0.52 ± 0.07
2	MCF7	0.95 ± 0.17	4.93 ± 0.45
3	HeLa	2.31 ± 0.08	4.67 ± 0.25
4	H9c2	1.12 ± 0.20	*ca*. 0.30 ± 0.08
5	HEK-293	1.16 ± 0.34	2.35 ± 0.69

## 3. Conclusion

In summary, based on our previous SAR studies of anthraquinone analogs with antileukemia activity, we conducted further structural modifications aimed at improving potency while maintaining its ability to downregulate MDM2. At first, we modified a hydroxyl group of the lead compound (**BW-AQ-238**) to a hydrogen bond acceptor ketone group at the terminal position of the alkyl sidechain. With such modification, **BW-AQ-260** exhibited significant improvement in potency (IC_50_ = 0.45 ± 0.02 μM) against EU-1 cells. By changing the alkyl chain length of **BW-AQ-260**, the C5 ketone side-chain analog **BW-AQ-295** was found to possess improved cytotoxicity, while further extending the sidechain length or removing one side-chain was found to lead to inferior activity. Further, the R^2^ substituent of the **BW-AQ-295** was examined. Replacing the chloroacetamide group with a propynamide group resulted in the most potent compound, **BW-AQ-350** (IC_50_ = 0.19 ± 0.01 μM), in this series of anthraquinone analogs. The activity was comparable to that of doxorubicin in the same ALL cell line. Further, Western-blot experiments showed the ability of **BW-AQ-295** and **BW-AQ-350** to downregulate MDM2 and upregulate p53 in EU-1 cells in a time- and dose-dependent fashion. Satisfying the key features of Lipinski’s Rule of Five^63^, **BW-AQ-350** and **BW-AQ-295** hold potential as potent candidates for treating ALL. Our future efforts are directed towards the assessment of their efficacy in animal models, addressing any potential pharmaceutical issues through either structural modification or prodrug design, and understanding the pharmacokinetic profiles of these compounds.

## 4. Materials and methods

### 4.1. Cell culture and cytotoxicity test

Cell viability was assessed by using Cell Counting Kit-8 (CCK-8, Dojindo, Japan). EU-1 and RS4;11 cells were cultured in RPMI-1640 medium (Corning, USA); MCF7 and HEK-293 cells were cultured in MEM (Corning, USA); and HeLa and H9c2 cells were cultured in high glucose DMEM (Corning, USA). All the culture mediums were supplemented with 10% foetal bovine serum (Corning, USA) and 100 unit/ml penicillin, and 0.1 μg/ml streptomycin. For cytotoxicity assays, cells were seeded into 96-well plates at a density of 3 × 10^5^ cells per well (50 μL). Compounds were dissolved in DMSO (molecular biology grade, Sigma-Aldrich, USA) to make 10 mM stock solutions, which were serially diluted with the culture medium to various concentrations (final DMSO concentration = 0.5%). A culture medium (50 μL) containing the anthraquinone analog was added directly to the cell suspension in each well immediately after seeding. After incubation for 24 h at 37 °C in a humidified atmosphere with 5% CO_2_, 10 μL of CCK-8 solution was added to each well, and the plate was incubated for an additional 2–4 h at 37 °C before measuring the optical density at 450 nm with a microplate reader (PerkinElmer Victor 2, USA). The cell viability of each well was calculated as the percentage of the untreated control according to the manufacturer’s manual. All tests were performed in triplicates, and IC_50_ values were determined with six concentrations by non-linear regression using GraphPad Prism 9.

### 4.2. Western-blot

5 × 10^6^ cells were seeded in a 6-well plate and incubated for 12 h before the drug-loaded medium was added. The cells were harvested at designated time points, washed with cold PBS, and centrifuged at 1500 rpm × 3 min. Then the cells were lysed by adding 100 µL of cold NP-40 buffer (supplied with cOmplete™ protease Inhibitor tablet (Roche, USA) and 1 mM PMSF (Thermo-Fisher, USA) on ice for 30 min. The cell lysates were centrifuged at 12,500 × g at 4 °C for 10 min, and the total protein concentration in the supernatant was measured with the BCA assay (Thermo-Fisher, USA). Thirty microlitres of the cell lysate was mixed with 10 µL 4× Laemmli sample buffer and denatured at 95 °C for 5 min. The total protein concentration was adjusted with 1× Laemmli sample buffer. Equal amounts of protein sample were loaded onto a 4–15% gradient SDS-PAGE gel (Bio-rad, USA). After electrophoresis, the protein was transferred to the PTFE membrane (Bio-rad, USA) with the Trans-Blot Turbo system (Bio-rad, USA). The target protein was probed with the corresponding antibody using iBind Western Systems (Thermo-Fisher, USA), according to the manufacturer’s manual. Antibody and dilution profile: MDM2 [(SMP14), 1:500 Santa Cruz, USA]; p53 [(DO-1), 1:800, Santa Cruz, USA]; GAPDH [(0411), 1:2000, Santa Cruz, USA] and HRP conjugated goat anti-mouse secondary antibody (1:2000, Bio-rad, USA). After incubation with Pierce ECL Plus Substrate (Thermo Scientific), chemiluminescent was detected and imaged with LSA4000 (GE Healthcare, Fairfield, USA).

### 4.3. Reactivity study by HPLC

HPLC was performed on Shimadzu LC-20AT HPLC system. Condition: mobile phase A: water with 0.05% TFA; B: acetonitrile with 0.05% TFA; gradient: 5–95% B, 0–10 min; 5–95% B 10–12 min; 5% B 12.1–15 min. Column: C18, 3.5 μm, 4.6 × 150 mm; UV detector monitored at 254 nm. For the thiol reactivity assay, a 0.3 mM solution of AQ-analogs was prepared in 0.5 ml PBS: MeOH (80:20) and warmed at 37 °C. Next, 25 µL of NAC (from 30 mM stock solution in PBS) was added to the above solution. The resultant solution had final concentrations of 0.3 mM AQ-analogs and 1.5 mM NAC. 20 µL of the reaction mixture was injected into the HPLC at the designated time intervals. The area percentage values were determined via the integration of the area under the curve (AUC) of the chromatogram.

### 4.4. Synthesis and characterisation of the compounds

#### 4.4.1. General information

Rhein was purchased from Nanjing Zelang Medical Technology Co. Ltd. (P.R. China) and used without further purification. All other starting materials were purchased from Sigma-Aldrich (USA) or Oakwood Chemical (USA), and analytical-grade solvents were purchased from Fisher Scientific (USA). Anhydrous chemical solvents were used for all moisture-sensitive reactions. High-resolution mass spectrometry (HRMS) analyses were conducted by the Georgia State University mass spectrometry facilities using ABI API 3200 (ESI-Triple Quadruple) instrument. ^1^H- (400 MHz) and ^13^C-NMR (100 MHz) spectra were recorded on a Bruker Avance 400 MHz NMR spectrometer. Deuterated solvents were purchased from Cambridge Isotope Laboratories, Inc (USA). Chemical shifts were reported as *δ* values (ppm). TMS (*δ* = 0.00 ppm) or residual peaks of the deuterated solvent were used as the internal reference.

##### 4.4.1.1. General procedure for the synthesis of compounds 1 and 7

Rhein (for compounds **1c** and **7**) or its methyl ester (for compounds **1a–b**) (1.676 mmol) was suspended in DMF (40 ml) in a dry round bottom flask. Next, K_2_CO_3_ (8.38 mmol) was added to the mixture, and the mixture was stirred gently for 5 min till the complete dissolution of all components. Further, an alkylating agent (for disubstituted-8.38 mmol; for mono-substituted 4.19 mmol) was added gradually. The solution was stirred for 4–5 h at 90–100 °C. After consumption of the starting material, according to TLC (DCM: methanol= 10:1), the reaction mixture was transferred to a conical flask having 200 ml of water. The compound was extracted by DCM, washed by 1 M HCl and brine, and dried *in-vacuo* to yield a yellow solid product in good yield and purity.

###### 4.4.1.1.1. Methyl 4,5-bis(but-3-en-1-yloxy)-9,10-dioxo-9,10-dihydroanthracene-2-carboxylate (**1a**)

Isolated yield: 86%. ^1^H-NMR (Chloroform-*d*) *δ* 8.45 (d, *J* = 1.6 Hz, 1H), 7.92 (d, *J* = 1.6 Hz, 1H), 7.87–7.83 (m, 1H), 7.63 (t, *J* = 8.0 Hz, 1H), 7.33–7.29 (m, 1H), 6.02–6.07 (m, 2H), 5.24–5.30 (m, 2H), 5.14–5.20 (m, 2H), 4.26 (t, *J* = 6.6 Hz, 2H), 4.20 (t, *J* = 6.7 Hz, 2H), 4.00 (s, 3H), 2.71 (t, *J* = 6.9 Hz, 4H). ^13^C-NMR (Chloroform-*d*) *δ* 183.3, 181.5, 165.5, 158.7, 134.9, 134.6, 134.5, 134.1, 134.0, 133.9, 127.4, 124.6, 119.9, 119.9, 119.8, 119.6, 119.2, 117.5, 117.4, 69.4, 69.4, 69.2, 52.7, 33.6, 33.5. HRMS (ESI) *m/z*: Calculated for C_24_H_23_O_6_ [M + H]^+^ 407.1496; Found 407.1495.

###### 4.4.1.1.2. Methyl 9,10-dioxo-4,5-bis(pent-4-en-1-yloxy)-9,10-dihydroanthracene-2-carboxylate (**1b**)

Isolated yield: 91%. ^1^H-NMR (Chloroform-*d*) *δ* 8.45 (s, 1H), 7.92 (s, 1H), 7.84 (d, *J* = 7.7 Hz, 1H), 7.63 (t, *J* = 8.0 Hz, 1H), 7.31 (d, *J* = 8.3 Hz, 1H), 5.99–5.84 (m, 2H), 5.16–5.07 (m, 2H), 5.02 (d, *J* = 10.0 Hz, 2H), 4.23 (t, *J* = 6.3 Hz, 2H), 4.17 (t, *J* = 6.3 Hz, 2H), 4.00 (s, 3H), 2.40 (dq, *J* = 10.7, 6.6 Hz, 4H), 2.04 (h, *J* = 6.8 Hz, 4H). ^13^C-NMR (Chloroform-*d*) *δ* 183.4, 181.6, 165.6, 158.8, 137.8, 137.7, 134.9, 134.7, 134.4, 133.8, 127.4, 124.5, 119.7, 119.6, 119.4, 119.0, 115.3, 115.3, 69.1, 68.8, 52.7, 29.9, 29.9, 28.2, 28.2. HRMS (ESI) *m/z*: Calculated for C_26_H_26_O_6_Na [M + Na]^+^ 457.1611; Found 457.1627.

###### 4.4.1.1.3. Hex-5-en-1-yl 4,5-bis(hex-5-en-1-yloxy)-9,10-dioxo-9,10-dihydroanthracene-2-carboxylate (**1c**)

Isolated yield: 78%. ^1^H-NMR (Chloroform-*d*) *δ* 8.42 (s, 1H), 7.90 (s, 1H), 7.82 (d, *J* = 7.5 Hz, 1H), 7.61 (t, *J* = 8.0 Hz, 1H), 7.29 (d, *J* = 8.3 Hz, 1H), 5.94–5.76 (m, 3H), 5.02 (dd, *J* = 31.0, 13.5 Hz, 6H), 4.38 (t, *J* = 6.6 Hz, 2H), 4.20 (t, *J* = 6.3 Hz, 2H), 4.14 (t, *J* = 6.2 Hz, 2H), 2.23–2.10 (m, 6H), 1.93 (dt, *J* = 13.0, 6.4 Hz, 4H), 1.87–1.78 (m, 2H), 1.75–1.63 (m, 4H), 1.56 (dt, *J* = 14.4, 7.3 Hz, 2H). ^13^C-NMR (Chloroform-*d*) *δ* 183.7, 181.7, 165.3, 159, 138.7, 138.7, 138.3, 135, 134.9, 134.8, 134, 127.4, 124.6, 119.7, 119.6, 119.5, 119, 115.1, 114.9, 114.9, 69.9, 69.6, 65.9, 33.5, 33.5, 33.4, 28.6, 28.5, 28.2, 25.3, 25.3, 25.2. HRMS (ESI) *m/z*: Calculated for C_33_H_39_O_6_ [M + H]^+^ 531.27; Found 531.2749.

###### 4.4.1.1.4. Pent-4-en-1-yl 5-hydroxy-9,10-dioxo-4-(pent-4-en-1-yloxy)-9,10-dihydroanthracene-2-carboxylate (**7**)

Isolated yield: 41%. ^1^H-NMR (Chloroform-*d*) *δ* 12.89 (s, 1H), 8.48 (d, *J* = 1.2 Hz, 1H), 7.92 (d, *J* = 0.9 Hz, 1H), 7.76 (d, *J* = 7.5 Hz, 1H), 7.62 (t, *J* = 7.9 Hz, 1H), 7.29 (d, *J* = 8.3 Hz, 1H), 5.96–5.80 (m, 2H), 5.15–5.00 (m, 4H), 4.40 (t, *J* = 6.6 Hz, 2H), 4.24 (t, *J* = 6.3 Hz, 2H), 2.40 (q, *J* = 7.0 Hz, 2H), 2.24 (q, *J* = 7.0 Hz, 2H), 2.10–2.01 (m, 2H), 1.97–1.88 (m, 2H). ^13^C-NMR (Chloroform-*d*) *δ* 188.1, 182.1, 164.9, 162.6, 160.5, 137.5, 137.3, 136.5, 136.1, 135.9, 132.6, 125, 123.4, 120.4, 119.5, 119.1, 117.1, 115.7, 115.7, 69.2, 65.6, 30.2, 30, 28.2, 27.8. HRMS (ESI) *m/z*: Calculated for C_25_H_25_O_6_ [M + H]^+^ 421.17; Found 421.1661.

##### 4.4.1.2. General procedure for the synthesis of compound 2 and 8

Compound **1** (for compound **2**) or **7** (for compound **8**) (0.257 mmol) was added to a mixture of THF and KOH (0.309 mmol) in water, and the mixture was stirred at room temperature. After, the completion of the reaction, monitored by TLC (hexane: ethyl acetate = 3:1), THF was removed *in-vacuo*, and residue was neutralised with 1% HCl and extracted by DCM. The solvent was reduced completely *in-vacuo* to afford the shiny-yellow product.

###### 4.4.1.2.1. 4,5-Bis(but-3-en-1-yloxy)-9,10-dioxo-9,10-dihydroanthracene-2-carboxylic acid (**2a**)

Isolated yield: 93%. ^1^H-NMR (DMSO-*d_6_*) *δ* 8.18 (d, *J* = 1.5 Hz, 1H), 7.94–7.87 (m, 1H), 7.83–7.68 (m, 2H), 7.57 (dt, *J* = 8.1, 2.4 Hz, 1H), 6.02–6.07 (m, 2H), 5.21–5.27 (m, 2H), 5.08–5.14 (m, 2H), 4.25 (t, *J* = 6.5 Hz, 2H), 4.19 (t, *J* = 6.5 Hz, 2H), 2.54–2.60 (m, 4H). ^13^C-NMR (DMSO-*d_6_*) *δ* 183.1, 181.0, 166.3, 158.5, 158.5, 135.8, 135.2, 135.1, 134.9, 134.7, 134.4, 126.8, 124.1, 120.8, 119.9, 119.1, 118.8, 117.7, 117.6, 69.1, 68.9, 33.5, 33.4. HRMS (ESI) *m/z*: Calculated for C_23_H_19_O_6_ [M − H]^−^ 391.1195; Found 391.1182.

###### 4.4.1.2.2. 9,10-Dioxo-4,5-bis(pent-4-en-1-yloxy)-9,10-dihydroanthracene-2-carboxylic acid (**2b**)

Isolated yield: 97%. ^1^H-NMR (DMSO-*d_6_*) *δ* 8.22–8.15 (m, 1H), 7.94–7.86 (m, 1H), 7.81–7.67 (m, 2H), 7.55 (dd, *J* = 8.2, 1.5 Hz, 1H), 5.87–5.95 (m, 2H), 5.10 (q, *J* = 1.9 Hz, 1H), 5.06 (p, *J* = 1.8 Hz, 1H), 4.99 (dt, *J* = 10.2, 1.8 Hz, 2H), 4.18 (dt, *J* = 21.9, 6.1 Hz, 4H), 2.39–2.28 (m, 4H), 1.95–1.82 (m, 4H). ^13^C-NMR (DMSO-*d_6_*) *δ* 183.3, 181.1, 166.8, 158.5, 138.5, 134.6, 134.4, 134.4, 126.0, 124.0, 120.3, 119.7, 119.0, 118.6, 115.5, 68.7, 68.6, 29.9, 29.9, 28.2, 28.2. HRMS (ESI) *m/z*: Calculated for C_25_H_13_O_6_ [M − H]^−^ 419.1489; Found 419.1495.

###### 4.4.1.2.3. 4,5-Bis(hex-5-en-1-yloxy)-9,10-dioxo-9,10-dihydroanthracene-2-carboxylic acid (**2c**)

Isolated yield: 91%. ^1^H-NMR (Chloroform-*d*) *δ* 8.50 (s, 1H), 7.94 (s, 1H), 7.82 (d, *J* = 7.6 Hz, 1H), 7.61 (t, *J* = 8.0 Hz, 1H), 7.28 (d, *J* = 8.4 Hz, 1H), 5.85 (tddd, *J* = 13.9, 10.6, 6.9, 3.7 Hz, 2H), 5.11–5.06 (m, 1H), 5.06–5.01 (m, 1H), 4.98 (d, *J* = 10.1 Hz, 2H), 4.22 (t, *J* = 6.3 Hz, 2H), 4.13 (t, *J* = 6.3 Hz, 2H), 2.18 (p, *J* = 6.7 Hz, 4H), 2.01–1.88 (m, 4H), 1.70 (dq, *J* = 15.2, 7.8 Hz, 4H). ^13^C-NMR (Chloroform-*d*) *δ* 183.4, 181.8, 169.9, 159, 159, 138.6, 138.6, 135.1, 134.7, 134.1, 133.8, 127.9, 124.4, 120.4, 119.7, 119.6, 119.1, 114.9, 114.9, 100.1, 69.9, 69.6, 33.4, 33.4, 28.5, 28.5, 25.2, 25.2. HRMS (ESI) *m/z*: Calculated for C_27_H_29_O_6_ [M − H]^−^ 447.515; Found 447.0411.

###### 4.4.1.2.4. 5-Hydroxy-9,10-dioxo-4-(pent-4-en-1-yloxy)-9,10-dihydroanthracene-2-carboxylic acid (**8**)

Isolated yield: 97%. ^1^H-NMR (Chloroform-*d*) *δ* 12.83 (s, 1H), 8.60 (s, 1H), 8.00 (s, 1H), 7.80 (d, *J* = 7.2 Hz, 1H), 7.64 (t, *J* = 7.8 Hz, 1H), 7.32 (d, *J* = 8.3 Hz, 1H), 5.92 (d, *J* = 6.4 Hz, 1H), 5.08 (dd, *J* = 27.6, 13.3 Hz, 2H), 4.28 (s, 2H), 2.42 (d, *J* = 6.9 Hz, 2H), 2.07 (d, *J* = 5.8 Hz, 2H). ^13^C-NMR (DMSO-d_6_) *δ* 187.6, 181.6, 165.7, 161.4, 160, 138, 137.4, 136.4, 135.2, 132.3, 124.4, 122.4, 119.3, 119.2, 118.4, 116.9, 115.3, 68.6, 40.1, 39.9, 39.1, 38.8, 29.5, 27.6. HRMS (ESI) *m/z*: Calculated for C_20_H_17_O_6_ [M + H]^+^ 353.10; Found 353.1014.

##### 4.4.1.3. General procedure for the synthesis of compounds 3 and 9

Compound **2** (for compound **3**) or **8** (for compound **9**) (0.05 mmol) was dissolved in DMF (4 ml). Further, Et_3_N (0.118 mmol) was added to the reaction solution. The solution was stirred at 0 °C for 5 min. Next, DPPA (0.113 mmol) was added, and the solution was stirred continuously at room temperature till starting material was consumed. After completion of the reaction, according to TLC (DCM: methanol= 10:1), about 10 ml of water was added to the mixture, and the light-yellow precipitate was obtained. The precipitate was filtered and washed generously with water.

###### 4.4.1.3.1. 4,5-Bis(but-3-en-1-yloxy)-9,10-dioxo-9,10-dihydroanthracene-2-carbonyl azide (**3a**)

Isolated yield: 89%. ^1^H-NMR (Chloroform-*d*) *δ* 8.55 (s, 1H), 7.98 (s, 1H), 7.87 (d, *J* = 7.6 Hz, 1H), 7.66 (t, *J* = 8.0 Hz, 1H), 7.33 (d, *J* = 8.5 Hz, 1H), 6.04–6.11 (m, 2H), 5.35–5.22 (m, 2H), 5.23–5.12 (m, 2H), 4.29 (t, *J* = 6.6 Hz, 2H), 4.22 (t, *J* = 6.7 Hz, 2H), 2.73 (p, *J* = 7.3 Hz, 4H). ^13^C-NMR (Chloroform-*d*) *δ* 183.2, 181.5, 168.8, 158.7, 158.7, 135.1, 134.6, 134.1, 134.0, 133.4, 128.0, 124.6, 120.6, 119.9, 119.8, 119.2, 117.6, 117.5, 69.5, 69.2, 33.6, 33.5. HRMS (ESI) *m/z*: Calculated for C_23_H_20_N_3_O_5_ [M + H]^+^ 418.1300; Found 418.1300.

###### 4.4.1.3.2. 9,10-Dioxo-4,5-bis(pent-4-en-1-yloxy)-9,10-dihydroanthracene-2-carbonyl azide (**3b**)

Isolated yield: 95%. ^1^H-NMR (Chloroform-*d*) *δ* 8.41 (d, *J* = 1.6 Hz, 1H), 7.88 (d, *J* = 1.6 Hz, 1H), 7.83 (d, *J* = 7.6 Hz, 1H), 7.64 (t, *J* = 8.0 Hz, 1H), 7.31 (d, *J* = 8.3 Hz, 1H), 5.98–5.83 (m, 2H), 5.07–5.15 (m, 2H), 4.99–5.04 (m, 2H), 4.19 (dt, *J* = 21.8, 6.3 Hz, 4H), 2.40 (p, *J* = 6.7 Hz, 4H), 2.05 (dp, *J* = 14.2, 6.7 Hz, 4H). ^13^C-NMR (Chloroform-*d*) *δ* 183.1, 181.3, 171.3, 158.9, 158.8, 137.7, 137.6, 135.1, 134.6, 134.5, 134.0, 128.2, 124.4, 119.6, 119.6, 119.0, 118.6, 115.4, 115.3, 69.1, 68.8, 29.9, 29.9, 28.2, 28.1. HRMS (ESI) *m/z*: Calculated for C_25_H_23_N_3_O_5_Na [M + Na]^+^ 468.1530; Found 468.1535.

###### 4.4.1.3.3. 4,5-Bis(hex-5-en-1-yloxy)-9,10-dioxo-9,10-dihydroanthracene-2-carbonyl azide (**3c**)

Isolated yield: 98%. ^1^H-NMR (Chloroform-*d*) *δ* 8.39 (d, *J* = 1.2 Hz, 1H), 7.85 (d, *J* = 1.0 Hz, 1H), 7.81 (d, *J* = 7.6 Hz, 1H), 7.61 (t, *J* = 8.0 Hz, 1H), 7.28 (d, *J* = 8.3 Hz, 1H), 5.92–5.78 (m, 2H), 5.11–5.02 (m, 2H), 4.98 (d, *J* = 10.2 Hz, 2H), 4.19 (t, *J* = 6.4 Hz, 2H), 4.13 (t, *J* = 6.4 Hz, 2H), 2.16 (t, *J* = 7.0 Hz, 4H), 1.99–1.87 (m, 4H), 1.75–1.63 (m, 4H). ^13^C-NMR (Chloroform-*d*) *δ* 183.2, 181.4, 171.4, 159.1, 159, 138.6, 138.6, 135.2, 134.7, 134.6, 134.1, 128.2, 124.4, 119.7, 119.6, 119, 118.6, 114.9, 114.9, 69.9, 69.6, 33.4, 33.4, 28.5, 28.4, 25.2, 25.2. HRMS (ESI) *m/z*: Calculated for C_27_H_28_N_3_O_5_ [M + H]^+^ 474.20; Found 474.2030.

###### 4.4.1.3.4. 5-Hydroxy-9,10-dioxo-4-(pent-4-en-1-yloxy)-9,10-dihydroanthracene-2-carbonyl azide (**9**)

Isolated yield: 99%. ^1^H-NMR (Chloroform-*d*) *δ* 12.86 (s, 1H), 8.51 (d, *J* = 1.0 Hz, 1H), 7.93 (s, 1H), 7.80 (d, *J* = 7.5 Hz, 1H), 7.65 (t, *J* = 7.9 Hz, 1H), 7.32 (d, *J* = 8.4 Hz, 1H), 5.90 (ddt, *J* = 17.0, 10.2, 6.7 Hz, 1H), 5.08 (dd, *J* = 25.9, 13.6 Hz, 2H), 4.26 (t, *J* = 6.3 Hz, 2H), 2.41 (q, *J* = 7.0 Hz, 2H), 2.08 (dd, *J* = 13.9, 6.7 Hz, 2H). ^13^C-NMR (Chloroform-*d*) *δ* 188, 1, 171.2, 162.7, 160.7, 137.5, 136.3, 136.2, 132.5, 125.2, 124.4, 120.4, 120.1, 119.3, 118.7, 117.2, 115.8, 69.4, 30, 28.2. HRMS (ESI) *m/z*: Calculated for C_20_H_16_N_3_O_5_ [M + H]^+^ 378.11; Found 378.1095.

##### 4.4.1.4. General procedure for the synthesis of compounds 4 and 10

Compound **3** (for compound **4**) or **9** (for compound **10**) (0.05 mmol) was refluxed in dry dioxane (3.5 ml) for 1 h under an argon atmosphere at a temperature of 130 °C. TLC analysis TLC (hexane: ethylacetate= 2:1) showed completion of the reaction. Next, the reaction mixture was diluted with water (10 ml) and heated at 50 °C for another 30 min, and then cooled to room temperature. The precipitate was filtered off and dried *in-vacuo* to afford a deep-red solid product.

###### 4.4.1.4.1. 3-Amino-1,8-bis(but-3-en-1-yloxy)anthracene-9,10-dione (**4a**)

Isolated yield: 59%. ^1^H-NMR (Chloroform-*d*) *δ* 7.78 (dt, *J* = 7.7, 1.6 Hz, 1H), 7.53 (td, *J* = 8.0, 2.8 Hz, 1H), 7.31–7.22 (m, 1H), 7.07 (d, *J* = 2.2 Hz, 1H), 6.48 (d, *J* = 2.2 Hz, 1H), 5.94–6.07 (m, 2H), 5.17–5.25 (m, 2H), 5.07–5.14 (m, 2H), 4.42 (s, 2H), 4.15 (t, *J* = 6.8 Hz, 2H), 4.04 (t, *J* = 6.8 Hz, 2H), 2.72–2.58 (m, 4H). ^13^C-NMR (Chloroform-*d*) *δ* 184.5, 180.9, 161.2, 158.7, 151.3, 136.2, 134.8, 134.3, 134.3, 134.3, 133.0, 132.9, 129.4, 124.8, 120.4, 119.2, 117.2, 117.2, 115.9, 105.1, 104.5, 69.4, 69.4, 69.0, 33.6, 33.6, 33.5. HRMS (ESI) *m/z*: Calculated for C_22_H_22_NO_4_Na [M + Na]^+^ 386.1352; Found 386.1368.

###### 4.4.1.4.2. 3-Amino-1,8-bis(pent-4-en-1-yloxy)anthracene-9,10-dione (**4b**)

Isolated yield: 77%. ^1^H-NMR (Chloroform-*d*) *δ* 7.79 (d, *J* = 7.6 Hz, 1H), 7.55 (t, *J* = 8.0 Hz, 1H), 7.35–7.24 (m, 1H), 7.07 (d, *J* = 2.2 Hz, 1H), 6.47 (d, *J* = 2.3 Hz, 1H), 5.85–5.94 (m, 2H), 5.09 (dt, *J* = 17.2, 1.8 Hz, 2H), 5.00 (dd, *J* = 10.1, 1.9 Hz, 2H), 4.14 (q, *J* = 6.2 Hz, 2H), 4.12–3.98 (m, 2H), 2.43–2.32 (m, 4H), 2.09–1.96 (m, 4H). ^13^C-NMR (Chloroform-*d*) *δ* 184.7, 180.8, 161.3, 158.9, 151.3, 138.0, 137.9, 136.3, 134.8, 132.8, 124.8, 120.1, 118.9, 116.0, 115.1, 104.6, 104.0, 77.3, 77.0, 76.7, 69.1, 68.7, 30.0, 30.0, 28.3, 28.2. HRMS (ESI) *m/z*: Calculated for C_24_H_25_NO_5_Na [M + Na]^+^ 414.1700; Found 414.1681.

###### 4.4.1.4.3. 3-Amino-1,8-bis(hex-5-en-1-yloxy)anthracene-9,10-dione (**4c**)

Isolated yield: 64%. ^1^H-NMR (Chloroform-*d*) *δ* 7.80 (d, *J* = 7.6 Hz, 1H), 7.55 (t, *J* = 8.0 Hz, 1H), 7.28 (s, 1H), 7.06 (d, *J* = 2.2 Hz, 1H), 6.47 (d, *J* = 2.1 Hz, 1H), 5.87 (ddt, *J* = 16.9, 10.1, 6.6 Hz, 2H), 5.07 (d, *J* = 17.1 Hz, 2H), 4.99 (d, *J* = 10.1 Hz, 2H), 4.31 (s, 2H), 4.13 (t, *J* = 6.5 Hz, 2H), 4.06 (t, *J* = 6.5 Hz, 2H), 2.18 (q, *J* = 7.0 Hz, 4H), 2.02–1.87 (m, 4H), 1.75–1.65 (m, 4H). ^13^C-NMR (Chloroform-*d*) *δ* 184.9, 181, 161.5, 159, 151.4, 138.8, 136.4, 134.9, 132.9, 124.8, 120.1, 119, 116.1, 114.8, 104.6, 104.1, 69.8, 69.4, 33.5, 33.5, 28.7, 28.6, 25.3. HRMS (ESI) *m/z*: Calculated for C_26_H_30_NO_4_ [M + H]^+^ 420.22; Found 420.2177.

###### 4.4.1.4.4. 3-Amino-8-hydroxy-1-(pent-4-en-1-yloxy)anthracene-9,10-dione (**10**)

Isolated yield: 56%. ^1^H-NMR (Chloroform-*d*) *δ* 13.55 (s, 1H), 7.70 (d, *J* = 7.3 Hz, 1H), 7.53 (t, *J* = 7.9 Hz, 1H), 7.25 (d, *J* = 8.7 Hz, 1H), 7.16 (d, *J* = 1.8 Hz, 1H), 6.42 (d, *J* = 1.7 Hz, 1H), 5.89 (ddt, *J* = 13.2, 10.0, 6.6 Hz, 1H), 5.06 (dd, *J* = 28.4, 13.3 Hz, 2H), 4.11 (t, *J* = 6.5 Hz, 2H), 2.38 (dd, *J* = 14.1, 7.0 Hz, 2H), 2.09–1.99 (m, 2H). ^13^C-NMR (Chloroform-*d*) *δ* 187, 183.5, 163.2, 162.5, 153.1, 151.5, 137.7, 137.5, 134.8, 132.8, 124.8, 118.5, 117.3, 115.6, 112.4, 106.5, 102.5, 68.7, 30, 29.8, 28.2. HRMS (ESI) *m/z*: Calculated for C_19_H_18_NO_4_ [M + H]^+^ 324.12; Found 324.1226.

##### 4.4.1.5. General procedure for the synthesis of compound 5 and BW-AQ-336

Compound **4** (for compound **5**) or compound **11** (for **BW-AQ-336**) (0.094 mmol) was dissolved in dry dioxane along with Et_3_N (0.141 mmol). The solution was cooled down to 0 °C. Further, chloroacetyl chloride (0.141 mmol) was added dropwise and stirred till the completion of the reaction, TLC (Hexane: ethyl acetate = 2:1). Then, the reaction mixture was diluted with DCM (20 ml), washed with water (3 × 10 ml), brine (3 × 10 ml) and dried over anhydrous Na_2_SO_4_. Further, the solvent was reduced completely *in-vacuo* and purified by silica gel column chromatography, yielding desired yellow solid product.

###### 4.4.1.5.1. N-(4,5-bis(but-3-en-1-yloxy)-9,10-dioxo-9,10-dihydroanthracen-2-yl)-2-chloroacetamide (**5a**)

Isolated yield: 92%. ^1^H-NMR (Chloroform-*d*) *δ* 8.52 (s, 1H), 8.15 (d, *J* = 2.2 Hz, 1H), 7.85 (dd, *J* = 7.7, 1.1 Hz, 1H), 7.62 (dd, *J* = 8.3, 7.7 Hz, 1H), 7.57 (d, *J* = 2.1 Hz, 1H), 7.32 (dd, *J* = 8.4, 1.1 Hz, 1H), 6.01–6.11 (m, 2H), 5.32–5.21 (m, 2H), 5.12–5.19 (m, 2H), 4.28–4.14 (m, 6H), 2.77–2.66 (m, 4H). ^13^C-NMR (Chloroform-*d*) *δ* 183.6, 180.9, 164.4, 160.3, 158.8, 141.5, 135.4, 134.6, 134.2, 134.1, 133.5, 124.5, 121.0, 120.2, 119.2, 117.5, 117.4, 109.8, 109.0, 69.3, 69.3, 42.9, 33.6, 33.5. HRMS (ESI) *m/z*: Calculated for C_24_H_22_NClO_5_Na [M + Na]^+^ 462.1079; Found 462.1084.

###### 4.4.1.5.2. 2-Chloro-N-(9,10-dioxo-4,5-bis(pent-4-en-1-yloxy)-9,10-dihydroanthracen-2-yl)acetamide (**5b**)

Isolated yield: 96%. ^1^H-NMR (Chloroform-*d*) *δ* 8.52 (s, 1H), 8.13 (d, *J* = 2.1 Hz, 1H), 7.84 (dd, *J* = 7.8, 1.1 Hz, 1H), 7.61 (t, *J* = 8.0 Hz, 1H), 7.57 (d, *J* = 2.1 Hz, 1H), 7.31 (dd, *J* = 8.4, 1.1 Hz, 1H), 5.92 (m, 2H), 5.14 (q, *J* = 1.8 Hz, 1H), 5.15–5.06 (m, 1H), 5.02 (dq, *J* = 10.2, 1.5 Hz, 2H), 4.25 (s, 2H), 4.18 (dt, *J* = 12.5, 6.3 Hz, 4H), 2.40, *J* = 8.0, 6.7, 1.5 Hz, 4H), 2.05 (dtd, *J* = 15.1, 6.5, 3.3 Hz, 4H). ^13^C-NMR (Chloroform-*d*) *δ* 183.7, 180.9, 164.2, 160.4, 159.0, 141.4, 137.8, 137.8, 135.5, 134.6, 133.5, 124.5, 121.0, 119.9, 119.0, 115.2, 109.6, 108.8, 69.0, 68.9, 42.8, 30.0, 29.9, 28.3, 28.1. HRMS (ESI) *m/z*: Calculated for C_26_H_26_NClO_5_Na [M + Na]^+^ 490.1421; Found 490.1397.

###### 4.4.1.5.3. N-(4,5-bis(hex-5-en-1-yloxy)-9,10-dioxo-9,10-dihydroanthracen-2-yl)-2-chloroacetamide (**5c**)

Isolated yield: 86%. ^1^H-NMR (Chloroform-*d*) *δ* 8.68 (s, 1H), 8.11 (d, *J* = 1.8 Hz, 1H), 7.79 (d, *J* = 7.6 Hz, 1H), 7.60–7.53 (m, 2H), 7.27 (d, *J* = 8.5 Hz, 1H), 5.84 (ddt, *J* = 16.9, 10.2, 6.6 Hz, 2H), 5.04 (dd, *J* = 17.1, 1.5 Hz, 2H), 4.96 (d, *J* = 10.2 Hz, 2H), 4.23 (s, 2H), 4.13 (dd, *J* = 13.9, 6.7 Hz, 4H), 2.15 (q, *J* = 7.1 Hz, 4H), 1.97–1.86 (m, 4H), 1.72–1.61 (m, 4H). ^13^C-NMR (Chloroform-*d*) *δ* 183.9, 181.1, 164.5, 160.6, 159.1, 141.7, 138.7, 135.5, 134.7, 133.6, 124.4, 120.9, 119.9, 119, 114.9, 114.8, 109.7, 108.9, 69.7, 69.7, 43, 33.5, 33.4, 28.6, 28.5, 25.2, 25.2. HRMS (ESI) *m/z*: Calculated for C_28_H_31_ClNO_5_ [M + H]^+^ 496.19; Found 496.1891.

###### 4.4.1.5.4. 2-Chloro-N-(5-hydroxy-9,10-dioxo-4-((4-oxopentyl)oxy)-9,10-dihydroanthracen-2-yl)acetamide (**BW-AQ-336**)

Isolated yield: 77%. ^1^H-NMR (Chloroform-*d*) *δ* 13.17 (s, 1H), 8.58 (s, 1H), 8.20 (s, 1H), 7.77 (d, *J* = 7.3 Hz, 1H), 7.62 (dd, *J* = 15.3, 7.2 Hz, 2H), 7.30 (d, *J* = 8.2 Hz, 1H), 4.25 (d, *J* = 6.3 Hz, 4H), 2.89 (t, *J* = 6.8 Hz, 2H), 2.22 (d, *J* = 6.5 Hz, 5H). ^13^C-NMR (Chloroform-*d*) *δ* 208.5, 187.5, 182.4, 164.6, 162.6, 162, 143.3, 136.6, 135.7, 132.6, 125.1, 77, 119, 117.4, 117.1, 110.3, 109, 68.7, 42.9, 39.7, 30.3, 29.8, 23, 22.8. HRMS (ESI) *m/z*: Calculated for C_21_H_19_ClNO_6_ [M + H]^+^ 416.09; Found 416.0887.

##### 4.4.1.6. General procedure for the synthesis of compounds 6, 11, BW-AQ-260, 295, and 345

Into a 20 ml vial, compound **4** (for compound **6**) or **5** (for **BW-AQ-260**, **−295**, and **−345**) or **10** (for compound **11**) (0.03 mmol) was added followed by 3 ml DMF/Water (1:1). Then PdCl_2_ (0.002 mmol) and CuCl (0.06 mmol) was added quickly, and a balloon of oxygen was plugged into the reaction mixture. Then the reaction was stirred overnight at room temperature. After TLC showed consumption of the starting material, the reaction mixture was diluted with water and extracted with DCM (5 ml, ×3). The combined organic layer was washed with water (50 ml) and brine (50 ml) successively and dried over anhydrous sodium sulphate. Then the solvent was removed *in-vacuo*, and the crude product was purified by silica-gel column chromatography (DCM/MeOH, 200/1). The product was isolated as a yellow solid.

###### 4.4.1.6.1. 3-Amino-1,8-bis((4-oxopentyl)oxy)anthracene-9,10-dione (**6**)

Isolated yield: 56%. ^1^H-NMR (Methanol-*d_4_*) *δ* 7.73 (d, *J* = 6.9 Hz, 1H), 7.61 (t, *J* = 8.0 Hz, 1H), 7.43 (d, *J* = 8.4 Hz, 1H), 7.00 (d, *J* = 2.1 Hz, 1H), 6.57 (d, *J* = 2.1 Hz, 1H), 4.15 (t, *J* = 6.1 Hz, 2H), 4.09 (t, *J* = 6.1 Hz, 2H), 2.86 (t, *J* = 7.1 Hz, 4H), 2.20 (s, 6H), 2.14–2.07 (m, 4H). ^13^C-NMR (Dichloromethane-*d_2_*) *δ* 208.7, 208.6, 184.7, 181, 161.4, 158.9, 152.3, 136.6, 135.2, 133.2, 124.9, 120.1, 119, 115.6, 104.6, 103.9, 68.8, 68.3, 39.9, 39.8, 30.2, 23.7, 23.6. HRMS (ESI) *m/z*: Calculated for C_24_H_26_NO_6_ [M + H]^+^ 424.465; Found 424.1769.

###### 4.4.1.6.2. 3-Amino-8-hydroxy-1-((4-oxopentyl)oxy)anthracene-9,10-dione (**11**)

Isolated yield: 58%. ^1^H-NMR (Chloroform-*d*) *δ* 13.56 (s, 1H), 7.69 (d, *J* = 7.0 Hz, 1H), 7.53 (t, *J* = 7.9 Hz, 1H), 7.24 (d, *J* = 8.3 Hz, 1H), 7.15 (d, *J* = 2.1 Hz, 1H), 6.44 (d, *J* = 2.0 Hz, 1H), 4.13 (t, *J* = 6.0 Hz, 2H), 2.88 (t, *J* = 6.7 Hz, 2H), 2.21 (s, 3H), 2.19–2.12 (m, 2H). ^13^C-NMR (Chloroform-*d*) *δ* 208.9, 187, 183.4, 163, 162.5, 153.3, 137.5, 134.8, 132.8, 124.8, 118.5, 117.3, 112.3, 106.5, 102.4, 68.1, 39.5, 30.3, 23. HRMS (ESI) *m/z*: Calculated for C_19_H_18_NO_5_ [M + H]^+^ 340.12; Found 340.1175.

###### 4.4.1.6.3. 2-Chloro-N-(9,10-dioxo-4,5-bis(3-oxobutoxy)-9,10-dihydroanthracen-2-yl)acetamide (**BW-AQ-260**)

Isolated yield: 90%. ^1^H-NMR (Chloroform-*d*) *δ* 8.54 (s, 1H), 8.11 (d, *J* = 2.1 Hz, 1H), 7.86 (dd, *J* = 7.7, 1.1 Hz, 1H), 7.71–7.59 (m, 2H), 7.38–7.31 (m, 1H), 4.38–4.46 (m, 4H), 4.25 (s, 2H), 3.07 (td, *J* = 6.2, 2.4 Hz, 4H), 2.39 (d, *J* = 3.5 Hz, 6H). ^13^C-NMR (Chloroform-*d*) *δ* 206.8, 206.6, 183.3, 164.3, 159.9, 158.5, 141.6, 137.4, 135.5, 135.4, 134.5, 133.7, 129.0, 120.3, 119.7, 109.9, 109.4, 65.3, 65.2, 42.8, 42.7, 42.5, 31.0. HRMS (ESI) *m/z*: Calculated for C_24_H_22_NClO_7_Na [M + Na]^+^ 494.0988; Found 494.0982.

###### 4.4.1.6.4. 2-Chloro-N-(9,10-dioxo-4,5-bis((4-oxopentyl)oxy)-9,10-dihydroanthracen-2-yl)acetamide (**BW-AQ-295**)

Isolated yield: 87%. ^1^H-NMR (Chloroform-*d*) *δ* 8.53 (s, 1H), 8.11 (d, *J* = 2.1 Hz, 1H), 7.85 (dd, *J* = 7.7, 1.1 Hz, 1H), 7.67–7.57 (m, 2H), 7.31 (dd, *J* = 8.4, 1.1 Hz, 1H), 4.27–4.15 (m, 6H), 2.91 (td, *J* = 6.9, 3.3 Hz, 4H), 2.30–2.11 (m, 10H). ^13^C-NMR (Chloroform-*d*) *δ* 208.6, 208.4, 183.5, 181.0, 164.3, 160.2, 158.7, 141.5, 135.5, 134.6, 133.7, 124.2, 120.7, 119.7, 119.2, 109.5, 109.0, 77.3, 77.0, 76.7, 68.4, 68.2, 42.8, 39.7, 39.5, 30.1, 30.1, 23.2, 23.1. HRMS (ESI) *m/z*: Calculated for C_26_H_26_NClO_7_Na [M + Na]^+^ 522.1287; Found 522.1295.

###### 4.4.1.6.5. 2-Chloro-N-(9,10-dioxo-4,5-bis((5-oxohexyl)oxy)-9,10-dihydroanthracen-2-yl)acetamide (**BW-AQ-345**)

Isolated yield: 39%. ^1^H-NMR (Chloroform-*d*) *δ* 8.68 (s, 1H), 8.08 (d, *J* = 1.9 Hz, 1H), 7.79 (d, *J* = 7.5 Hz, 1H), 7.61–7.53 (m, 2H), 7.29–7.24 (m, 1H), 4.22 (s, 2H), 4.13 (q, *J* = 6.0 Hz, 4H), 2.60 (t, *J* = 6.7 Hz, 4H), 2.18 (s, 6H), 1.88 (tt, *J* = 6.3, 4.0 Hz, 8H). ^13^C-NMR (Chloroform-*d*) *δ* 209.3, 209.2, 183.6, 181.1, 164.8, 160.3, 158.9, 142, 135.4, 134.6, 133.6, 124.1, 120.5, 119.8, 119.1, 109.6, 109.1, 69.4, 69.3, 43.2, 43.1, 29.9, 29.9, 28.5, 28.3, 20.4. HRMS (ESI) *m/z*: Calculated for C_28_H_31_ClNO_7_ [M + H]^+^ 528.18; Found 528.1793.

##### 4.4.1.7. Procedure for the synthesis of BW-AQ-350

Compound **6** (0.025 mmol), propiolic acid (0.031), and DMAP (0.038) were mixed in dry DCM and stirred at an ice-bath temperature under argon protection. After 5 min, EDC (0.038) was added to the above reaction mixture. The reaction was monitored by TLC (DCM: MeOH = 20:1). After the completion of the reaction, DCM (10 ml) was added to the reaction solution and washed with water and brine. Next, the organic layer was dried over dry Na_2_SO_4_ and concentrated *in-vacuo*. The crude product was purified by silica gel column chromatography to yield a yellow product.

###### 4.4.1.7.1. N-(9,10-dioxo-4,5-bis((4-oxopentyl)oxy)-9,10-dihydroanthracen-2-yl)propiolamide (**BW-AQ-350**)

Isolated yield: 82%. ^1^H-NMR (Chloroform-*d*) *δ* 8.28 (s, 1H), 8.05 (s, 1H), 7.79 (d, *J* = 7.6 Hz, 1H), 7.57 (dd, *J* = 17.1, 9.2 Hz, 2H), 7.28 (d, *J* = 8.6 Hz, 1H), 4.17 (t, *J* = 5.9 Hz, 4H), 3.03 (s, 1H), 2.88 (dd, *J* = 11.2, 6.8 Hz, 4H), 2.23 (s, 6H), 2.17 (td, *J* = 6.3, 3.2 Hz, 4H). ^13^C-NMR (Chloroform-*d*) *δ* 208.9, 208.9, 208.8, 208.5, 183.6, 181.5, 181.1, 179, 160.3, 160, 158.8, 156.2, 149.9, 149.5, 142, 136.7, 135.5, 134.7, 134.6, 133.9, 133.8, 131.8, 129.2, 124.2, 122.3, 120.6, 120, 119.8, 119.5, 119.3, 118.6, 110, 109.7, 108.9, 103.5, 100.1, 75.4, 68.5, 68.3, 39.9, 39.7, 30.3, 30.2, 29.8, 23.3, 23.2. HRMS (ESI) *m/z*: Calculated for C_27_H_25_NO_7_ [M + Na]^+^ 498.1535; Found 498.1543.

##### 4.4.1.8. Procedure for the synthesis of BW-AQ-349 and BW-AQ-353

Compound **6** (0.094 mmol) was dissolved in dry dioxane along with Et_3_N (0.141 mmol). The solution was cooled down to 0 °C. Acetyl chloride (for **BW-AQ-349**)/acryloyl chloride (for **BW-AQ-353**) (0.141 mmol) was added dropwise and stirred till the completion of the reaction, TLC (Hexane: ethyl acetate= 2:1). After completion of the reaction, the reaction was diluted with water (10 ml) and extracted by DCM (3 × 10 ml). The combined organic phase was washed by brine (3 × 10 ml) and dried over anhydrous Na_2_SO_4_. Next, the solvent was completely removed *in-vacuo* and purified by silica gel column chromatography, yielding desired yellow solid product.

###### 4.4.1.8.1. N-(9,10-dioxo-4,5-bis((4-oxopentyl)oxy)-9,10-dihydroanthracen-2-yl)acetamide (**BW-AQ-349**)

Isolated yield: 73%. ^1^H-NMR (Methanol-*d_4_*) *δ* 7.84 (dd, *J* = 14.1, 4.9 Hz, 1H), 7.73 (dt, *J* = 17.8, 5.9 Hz, 2H), 7.61 (dd, *J* = 16.3, 8.2 Hz, 1H), 7.39 (dd, *J* = 17.5, 8.5 Hz, 1H), 4.18–4.07 (m, 4H), 2.85 (td, *J* = 7.0, 3.2 Hz, 4H), 2.21 (d, *J* = 1.2 Hz, 6H), 2.12 (dt, *J* = 11.5, 4.4 Hz, 7H). ^13^C-NMR (Chloroform-*d*) *δ* 209, 209, 183.9, 181.1, 169.2, 160.3, 158.8, 143.3, 135.2, 134.7, 133.6, 124.2, 119.7, 119.1, 109.5, 108.7, 77.4, 77.3, 77.1, 76.8, 68.4, 68.3, 40, 39.7, 30.3, 30.2, 24.9, 23.3. HRMS (ESI) *m/z*: Calculated for C_26_H_27_NO_7_ [M + H]^+^ 464.1788; Found 464.1036.

###### 4.4.1.8.2. N-(9,10-dioxo-4,5-bis((4-oxopentyl)oxy)-9,10-dihydroanthracen-2-yl)acrylamide (**BW-AQ-353**)

Isolated yield: 44%. ^1^H-NMR (Chloroform-*d*) *δ* 8.61 (s, 1H), 8.28 (s, 1H), 7.71 (d, *J* = 7.5 Hz, 1H), 7.53 (t, *J* = 8.0 Hz, 1H), 7.45 (d, *J* = 1.4 Hz, 1H), 7.23 (d, *J* = 8.3 Hz, 1H), 6.39 (dt, *J* = 16.8, 13.2 Hz, 2H), 5.80 (d, *J* = 10.3 Hz, 1H), 4.15 (dd, *J* = 11.1, 5.6 Hz, 4H), 2.87 (dd, *J* = 12.6, 6.6 Hz, 4H), 2.24 (s, 6H), 2.16 (dd, *J* = 11.3, 5.8 Hz, 4H). ^13^C-NMR (Chloroform-*d*) *δ* 209.1, 209, 183.8, 181.1, 164.1, 160.3, 158.8, 143.1, 135.2, 134.6, 133.7, 130.8, 129.2, 124.2, 119.8, 119.7, 119.1, 109.6, 108.9, 68.4, 68.3, 40, 39.8, 30.3, 30.2, 29.8, 23.3. HRMS (ESI) *m/z*: Calculated for C_27_H_27_NO_7_ [M + H]^+^= 478.5130; Found 478.188.

##### 4.4.1.9. Procedure for the synthesis of BW-AQ-354

Compound **6** (0.118 mmol) and Et_3_N (0.354 mmol) was stirred in dry DCM at room temperature. Next, 2-chloroethanesulfonyl chloride (0.014 mmol) was added dropwise to the reaction mixture and stirred till the completion of the reaction. Further, the reaction mixture was diluted with water (10 ml), extracted with DCM (3 × 10 ml), dried over dry sodium sulphate, and concentrated *in-vacuo*. After purification by silica gel column chromatography, the intermediate compound (0.103 mmol) was further treated with TBAF (0.113 mmol) in THF (3 ml) and monitored by TLC till completion of the reaction. Next, the reaction was quenched by adding water (10 ml), extracted with DCM (3 × 10 ml), and the combined organic phase was further dried over dry sodium sulphate. After complete removal of the solvent *in-vacuo*, the crude product was purified by silica-gel column chromatography to yield desired yellow-solid product.

###### 4.4.1.9.1. N-(9,10-dioxo-4,5-bis((4-oxopentyl)oxy)-9,10-dihydroanthracen-2-yl)ethenesulfonamide (**BW-AQ-354**)

Isolated yield: 77%. ^1^H-NMR (Chloroform-*d*) *δ* 7.83 (d, *J* = 7.2 Hz, 1H), 7.60 (t, *J* = 8.0 Hz, 1H), 7.48 (d, *J* = 1.9 Hz, 1H), 7.38 (s, 1H), 7.29 (d, *J* = 7.9 Hz, 1H), 7.22 (d, *J* = 2.1 Hz, 1H), 6.62 (dd, *J* = 16.5, 9.8 Hz, 1H), 6.44 (s, 1H), 6.40 (s, 1H), 6.06 (d, *J* = 9.8 Hz, 1H), 4.15 (dt, *J* = 8.7, 6.0 Hz, 4H), 2.88 (t, *J* = 6.7 Hz, 4H), 2.25–2.12 (m, 10H). ^13^C-NMR (Chloroform-*d*) *δ* 208.8, 208.7, 183.8, 181.1, 160.4, 158.8, 141.7, 136, 135.1, 134.5, 133.9, 129.6, 124.1, 120.6, 119.9, 119.4, 108.8, 108.5, 68.5, 68.4, 39.7, 39.6, 30.2, 29.8, 23.3, 23.1. HRMS (ESI) *m/z*: Calculated for C_26_H_27_NO_8_S [M + Na]^+^ 536.5610; Found 536.134.

## Supplementary Material

Supplemental Material

## References

[1] Turcotte LM, Hocutt C, Messinger YH, et al. Cost of pediatric acute lymphoblastic leukemia care in the current treatment era. Blood 2021;138:663.

[2] Tyner JW, Jemal AM, Thayer M, et al. Targeting survivin and p53 in pediatric acute lymphoblastic leukemia. Leukemia 2012;26:623–32. 21960246 10.1038/leu.2011.249 PMC3364442

[3] Yuan S, Wang X, Hou S, et al. PHF6 and JAK3 mutations cooperate to drive T-cell acute lymphoblastic leukemia progression. Leukemia 2022;36:370–82. 34465864 10.1038/s41375-021-01392-1 PMC8807395

[4] Amaral JD, Xavier JM, Steer CJ, Rodrigues CM. The role of p53 in apoptosis. Discov Med 2010;9:145–52. 20193641

[5] Wade M, Li Y-C, Wahl GM. MDM2, MDMX and p53 in oncogenesis and cancer therapy. Nat Rev Cancer 2013;13:83–96. 23303139 10.1038/nrc3430 PMC4161369

[6] Piette J, Neel H, Maréchal V. Mdm2: keeping p53 under control. Oncogene 1997;15:1001–10. 9285554 10.1038/sj.onc.1201432

[7] Urso L, Calabrese F, Favaretto A, et al. Critical review about MDM2 in cancer: possible role in malignant mesothelioma and implications for treatment. Crit Rev Oncol Hematol 2016;97:220–30. 26358421 10.1016/j.critrevonc.2015.08.019

[8] Watanabe T, Ichikawa A, Saito H, Hotta T. Overexpression of the MDM2 oncogene in leukemia and lymphoma. Leuk Lymphoma 1996;21:391–7, color plates XVI following 5. 9172803 10.3109/10428199609093436

[9] Kojima K, Ishizawa J, Andreeff M. Pharmacological activation of wild-type p53 in the therapy of leukemia. Exp Hematol 2016;44:791–8. 27327543 10.1016/j.exphem.2016.05.014 PMC5062953

[10] Gu L, Zhu N, Findley HW, Zhou M. MDM2 antagonist nutlin-3 is a potent inducer of apoptosis in pediatric acute lymphoblastic leukemia cells with wild-type p53 and overexpression of MDM2. Leukemia 2008;22:730–9. 18273046 10.1038/leu.2008.11 PMC3477706

[11] Trino S, De Luca L, Laurenzana I, et al. P53-MDM2 pathway: evidences for a new targeted therapeutic approach in B-acute lymphoblastic leukemia. Front Pharmacol 2016;7:491. 28018226 10.3389/fphar.2016.00491 PMC5159974

[12] Han X, Garcia-Manero G, McDonnell TJ, et al. HDM4 (HDMX) is widely expressed in adult pre-B acute lymphoblastic leukemia and is a potential therapeutic target. Mod Pathol 2007;20:54–62. 17143258 10.1038/modpathol.3800727

[13] Anifowose A, Agbowuro AA, Yang X, Wang B. Anticancer strategies by upregulating p53 through inhibition of its ubiquitination by MDM2. Med Chem Res 2020;29:1105–21.

[14] Shangary S, Wang S. Small-molecule inhibitors of the MDM2-p53 protein-protein interaction to reactivate p53 function: a novel approach for cancer therapy. Annu Rev Pharmacol Toxicol 2009;49:223–41. 18834305 10.1146/annurev.pharmtox.48.113006.094723 PMC2676449

[15] Zhu N, Gu L, Li F, Zhou M. Inhibition of the Akt/survivin pathway synergizes the antileukemia effect of nutlin-3 in acute lymphoblastic leukemia cells. Mol Cancer Ther 2008;7:1101–9. 18483299 10.1158/1535-7163.MCT-08-0179

[16] Trino S, Iacobucci I, Erriquez D, et al. Targeting the p53-MDM2 interaction by the small-molecule MDM2 antagonist Nutlin-3a: a new challenged target therapy in adult Philadelphia positive acute lymphoblastic leukemia patients. Oncotarget 2016;7:12951–61. 26887044 10.18632/oncotarget.7339 PMC4914334

[17] Ravandi F, Gojo I, Patnaik MM, et al. A phase I trial of the human double minute 2 inhibitor (MK-8242) in patients with refractory/recurrent acute myelogenous leukemia (AML). Leuk Res 2016;48:92–100. 27544076 10.1016/j.leukres.2016.07.004 PMC5408350

[18] Draganov AB, Yang X, Anifowose A, et al. Upregulation of p53 through induction of MDM2 degradation: anthraquinone analogs. Bioorg Med Chem 2019;27:3860–3865. 31324563 10.1016/j.bmc.2019.07.019

[19] Bernal F, Tyler AF, Korsmeyer SJ, et al. Reactivation of the p53 tumor suppressor pathway by a stapled p53 peptide. J Am Chem Soc 2007;129:2456–2457. 17284038 10.1021/ja0693587 PMC6333086

[20] Bernal F, Wade M, Godes M, et al. A stapled p53 helix overcomes HDMX-mediated suppression of p53. Cancer Cell 2010;18:411–22. 21075307 10.1016/j.ccr.2010.10.024 PMC3050021

[21] Zhang H, Gu L, Liu T, et al. Inhibition of MDM2 by nilotinib contributes to cytotoxicity in both Philadelphia-positive and negative acute lymphoblastic leukemia. PLOS One 2014;9:e100960. 24968304 10.1371/journal.pone.0100960 PMC4072773

[22] Huang M, Zhang H, Liu T, et al. Triptolide inhibits MDM2 and induces apoptosis in acute lymphoblastic leukemia cells through a p53-independent pathway. Mol Cancer Ther 2013;12:184–94. 23243057 10.1158/1535-7163.MCT-12-0425 PMC3570632

[23] Li Y, Yang J, Aguilar A, et al. Discovery of MD-224 as a first-in-class, highly potent, and efficacious proteolysis targeting chimera murine double minute 2 degrader capable of achieving complete and durable tumor regression. J Med Chem 2019;62:448–66. 30525597 10.1021/acs.jmedchem.8b00909 PMC6545112

[24] He S, Ma J, Fang Y, et al. Homo-PROTAC mediated suicide of MDM2 to treat non-small cell lung cancer. Acta Pharm Sin B 2021;11:1617–28. 34221872 10.1016/j.apsb.2020.11.022 PMC8245912

[25] Anifowose A, Yuan Z, Yang X, et al. Upregulation of p53 through induction of MDM2 degradation: amino acid prodrugs of anthraquinone analogs. Bioorg Med Chem Lett 2020;30:126786. 31753697 10.1016/j.bmcl.2019.126786 PMC6942214

[26] Anifowose A, Agbowuro AA, Tripathi R, et al. Inducing apoptosis through upregulation of p53: structure-activity exploration of anthraquinone analogs. Med Chem Res 2020;29:1199–210. 32719577 10.1007/s00044-020-02563-y PMC7384666

[27] Yang X, Sun G, Yang C, Wang B. Novel rhein analogues as potential anticancer agents. ChemMedChem 2011;6:2294–301. 21954017 10.1002/cmdc.201100384

[28] Gu L, Zhang H, Liu T, et al. Inhibition of MDM2 by a rhein-derived compound AQ-101 suppresses cancer development in SCID mice. Mol Cancer Ther 2018;17:497–507. 29282301 10.1158/1535-7163.MCT-17-0566 PMC6054458

[29] Malik MS, Alsantali RI, Jassas RS, et al. Journey of anthraquinones as anticancer agents – a systematic review of recent literature. RSC Adv 2021;11:35806–27. 35492773 10.1039/d1ra05686g PMC9043427

[30] Damiani RM, Moura DJ, Viau CM, et al. Pathways of cardiac toxicity: comparison between chemotherapeutic drugs doxorubicin and mitoxantrone. Arch Toxicol 2016;90:2063–76. 27342245 10.1007/s00204-016-1759-y

[31] Paul F, Dörr J, Würfel J, et al. Early mitoxantrone-induced cardiotoxicity in secondary progressive multiple sclerosis. J Neurol Neurosurg Psychiatry 2007;78:198–200. 17229751 10.1136/jnnp.2006.091033 PMC2077678

[32] Menna P, Salvatorelli E, Giampietro R, et al. Doxorubicin-dependent reduction of ferrylmyoglobin and inhibition of lipid peroxidation: implications for cardiotoxicity of anticancer anthracyclines. Chem Res Toxicol 2002;15:1179–89. 12230412 10.1021/tx020055+

[33] Singal PK, Li T, Kumar D, et al. Adriamycin-induced heart failure: mechanism and modulation. Mol Cell Biochem 2000;207:77–86. 10888230 10.1023/a:1007094214460

[34] Liu J, Mao W, Ding B, Liang C-s. ERKs/p53 signal transduction pathway is involved in doxorubicin-induced apoptosis in H9c2 cells and cardiomyocytes. Am J Physiol Heart Circ Physiol 2008;295:H1956–65. 18775851 10.1152/ajpheart.00407.2008 PMC2614569

[35] Guin PS, Das S. Exploration of electrochemical intermediates of the anticancer drug doxorubicin hydrochloride using cyclic voltammetry and simulation studies with an evaluation for its interaction with DNA. Int J Electrochem 2014;2014:1–8.

[36] Fei J, Peng Y, Tan H, et al. Study on the electrochemical behavior and differential pulse voltammetric determination of rhein using a nanoparticle composite film-modified electrode. Bioelectrochemistry 2007;70:369–74. 16820331 10.1016/j.bioelechem.2006.05.007

[37] Pan L, Yang K, Li G, Ge H. Palladium-catalyzed site-selective arylation of aliphatic ketones enabled by a transient ligand. Chem Commun 2018;54:2759–62. 10.1039/c8cc00980e 29480302

[38] Younis IR, Lakota EA, Volpe DA, et al. Drug-drug interaction studies of methadone and antiviral drugs: lessons learned. J Clin Pharmacol 2019;59:1035–43. 30973652 10.1002/jcph.1405

[39] Gehringer M, Laufer SA. Emerging and re-emerging warheads for targeted covalent inhibitors: applications in medicinal chemistry and chemical biology. J Med Chem 2019;62:5673–5724. 30565923 10.1021/acs.jmedchem.8b01153

[40] Cully M. Novel chemistry for covalent inhibitors. Nat Rev Drug Discov 2020;19:754. 32913211 10.1038/d41573-020-00161-6

[41] Singh J, Petter RC, Baillie TA, Whitty A. The resurgence of covalent drugs. Nat Rev Drug Discov 2011;10:307–17. 21455239 10.1038/nrd3410

[42] Sutanto F, Konstantinidou M, Dömling A. Covalent inhibitors: a rational approach to drug discovery. RSC Med Chem 2020;11:876–84. 33479682 10.1039/d0md00154f PMC7557570

[43] Mons E, Jansen IDC, Loboda J, et al. The alkyne moiety as a latent electrophile in irreversible covalent small molecule inhibitors of cathepsin K. J Am Chem Soc 2019;141:3507–14. 30689386 10.1021/jacs.8b11027 PMC6396318

[44] Amishiro N, Nagamura S, Kobayashi E, et al. Synthesis and antitumor activity of duocarmycin derivatives: A-ring pyrrole compounds bearing β-(5′,6′,7′-trimethoxy-2′-indolyl)acryloyl group. Bioorg Med Chem 2000;8:1637–43. 10976511 10.1016/s0968-0896(00)00086-9

[45] Dimmock JR, Padmanilayam MP, Puthucode RN, et al. A conformational and structure-activity relationship study of cytotoxic 3,5-bis(arylidene)-4-piperidones and related N-acryloyl analogues. J Med Chem 2001;44:586–93. 11170648 10.1021/jm0002580

[46] Van Herck N, Maes D, Unal K, et al. Covalent adaptable networks with tunable exchange rates based on reversible thiol-yne cross-linking. Angew Chem Int Ed Engl 2020;59:3609–17. 31846194 10.1002/anie.201912902

[47] Henise JC, Taunton J. Irreversible Nek2 kinase inhibitors with cellular activity. J Med Chem 2011;54:4133–46. 21627121 10.1021/jm200222m PMC3663048

[48] Harada H, Kazami J, Watanuki S, et al. Ethenesulfonamide and ethanesulfonamide derivatives, a novel class of orally active endothelin-A receptor antagonists. Bioorg Med Chem 2001;9:2955–68. 11597477 10.1016/s0968-0896(01)00187-0

[49] Flanagan ME, Abramite JA, Anderson DP, et al. Chemical and computational methods for the characterization of covalent reactive groups for the prospective design of irreversible inhibitors. J Med Chem 2014;57:10072–9. 25375838 10.1021/jm501412a

[50] Petri L, Ábrányi-Balogh P, Varga PR, et al. Comparative reactivity analysis of small-molecule thiol surrogates. Bioorg Med Chem 2020;28:115357. 32081630 10.1016/j.bmc.2020.115357

[51] Costa AM, Bosch L, Petit E, Vilarrasa J. Computational study of the addition of methanethiol to 40+ Michael acceptors as a model for the bioconjugation of cysteines. J Org Chem 2021;86:7107–18. 33914532 10.1021/acs.joc.1c00349 PMC8631706

[52] Kobayashi T, Hoppmann C, Yang B, Wang L. Using protein-confined proximity to determine chemical reactivity. J Am Chem Soc 2016;138:14832–5. 27797495 10.1021/jacs.6b08656 PMC5310709

[53] Li Q, Chen Q, Klauser PC, et al. Developing covalent protein drugs via proximity-enabled reactive therapeutics. Cell 2020;182:85–97.e16. 32579975 10.1016/j.cell.2020.05.028

[54] Alkhalaf M, El-Mowafy AM. Overexpression of wild-type p53 gene renders MCF-7 breast cancer cells more sensitive to the antiproliferative effect of progesterone. J Endocrinol 2003;179:55–62. 14529565 10.1677/joe.0.1790055

[55] Zhao Y, Yu S, Sun W, et al. A potent small-molecule inhibitor of the MDM2-p53 interaction (MI-888) achieved complete and durable tumor regression in mice. J Med Chem 2013;56:5553–61. 23786219 10.1021/jm4005708 PMC3880646

[56] Hoppe-Seyler F, Butz K. Repression of endogenous p53 transactivation function in HeLa cervical carcinoma cells by human papillomavirus type 16 E6, human mdm-2, and mutant p53. J Virol 1993;67:3111–7. 8388491 10.1128/jvi.67.6.3111-3117.1993 PMC237648

[57] Xie W, Zhang W, Sun M, et al. Deacetylmycoepoxydiene is an agonist of Rac1, and simultaneously induces autophagy and apoptosis. Appl Microbiol Biotechnol 2018;102:5965–75. 29740674 10.1007/s00253-018-9058-6

[58] Watkins SJ, Borthwick GM, Arthur HM. The H9C2 cell line and primary neonatal cardiomyocyte cells show similar hypertrophic responses *in vitro*. In Vitro Cell Dev Biol Anim 2011;47:125–31. 21082279 10.1007/s11626-010-9368-1

[59] Lin Y-C, Boone M, Meuris L, et al. Genome dynamics of the human embryonic kidney 293 lineage in response to cell biology manipulations. Nat Commun 2014;5:4767. 25182477 10.1038/ncomms5767 PMC4166678

[60] Belzile J-P, Karatzas A, Shiu H-Y, et al. Increased resistance to nitrogen mustards and antifolates following *in vitro* selection of murine fibroblasts and primary hematopoietic cells transduced with a bicistronic retroviral vector expressing the rat glutathione S-transferase A3 and a mutant dihydrofolate reductase. Cancer Gene Ther 2003;10:637–46. 12872145 10.1038/sj.cgt.7700619

[61] Holbeck SL, Collins JM, Doroshow JH. Analysis of Food and Drug Administration-approved anticancer agents in the NCI60 panel of human tumor cell lines. Mol Cancer Ther 2010;9:1451–60. 20442306 10.1158/1535-7163.MCT-10-0106 PMC2868078

[62] Chen Y, Jia Y, Song W, Zhang L. Therapeutic potential of nitrogen mustard based hybrid molecules. Front Pharmacol 2018;9:1453. 30618747 10.3389/fphar.2018.01453 PMC6304445

[63] Lipinski CA, Lombardo F, Dominy BW, Feeney PJ. Experimental and computational approaches to estimate solubility and permeability in drug discovery and development settings. Adv Drug Deliv Rev 2001;46:3–26. 11259830 10.1016/s0169-409x(00)00129-0

