# Supplementary material for: Upregulation of p53 through induction of MDM2 degradation: improved potency through the introduction of an alkylketone sidechain on the anthraquinone core
Source: J Enzyme Inhib Med Chem. 2022 Aug 31;37(1):2370–81. doi: 10.1080/14756366.2022.2116699 (PMC9448394; doi:10.1080/14756366.2022.2116699)

Supporting Information

Upregulation of p53 through Induction of MDM2 Degradation: Improved Potency through the Introduction of an Alkylketone Sidechain on the Anthraquinone Core

Ravi Tripathi, Abiodun Anifowose, Wen Lu, Xiaoxiao Yang*, and Binghe Wang*

Department of Chemistry and Center for Diagnostics and Therapeutics, Georgia State University, Atlanta, GA 30303, United States

*Corresponding authors: X.Y.: xyang20@gsu.edu; B.W.: wang@gsu.edu, +1(404)-413-5544

Table of Contents

1. Supplemental Figures……………………..…….………………………………………2-5

3. NMR spectra…………………………………………………..…………….………………6-34

Information Classification: General

1. Supplemental Figures

Figure S1. Cytotoxicity study results in EU-1 cell and other cell lines.

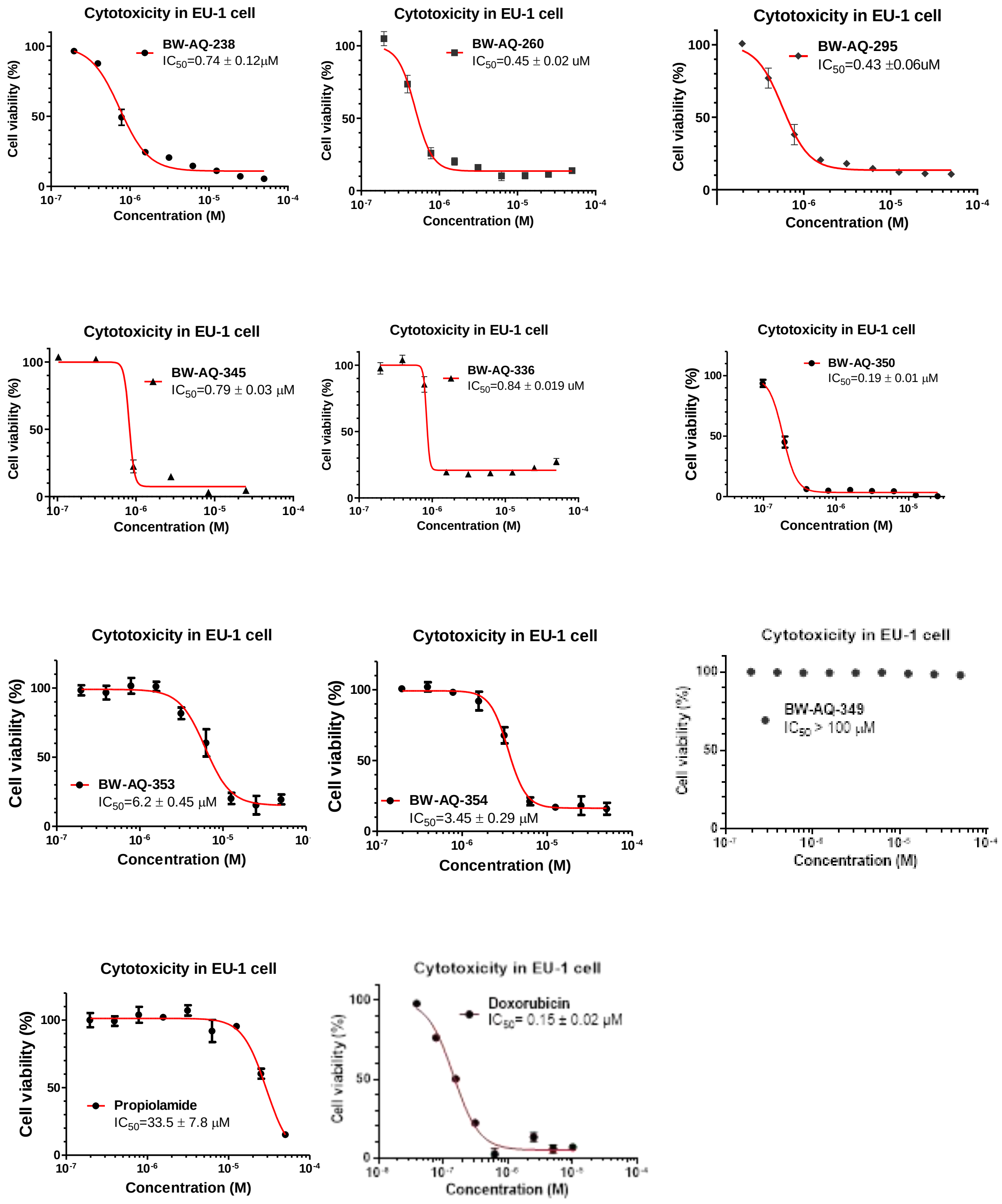

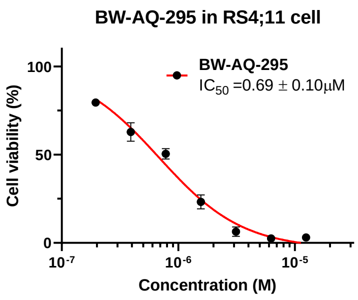
BW-AQ-295 in RS4;11 cell
BW-AQ-295
IC50 =0.69 ± 0.10μM
Cell viability (%)
Concentration (M)

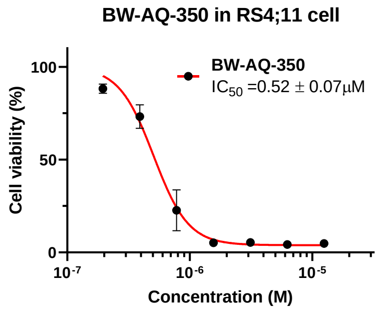
BW-AQ-350 in RS4;11 cell
BW-AQ-350
IC50 =0.52 ± 0.07μM
Cell viability (%)
Concentration (M)

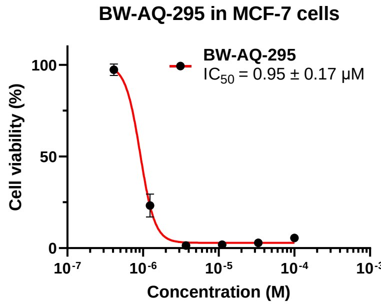
BW-AQ-295 in MCF-7 cells
BW-AQ-295
IC50 = 0.95 ± 0.17 μM
Cell viability (%)
Concentration (M)

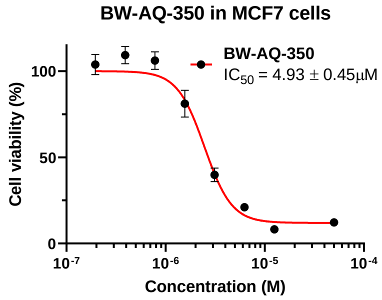
BW-AQ-350 in MCF7 cells
BW-AQ-350
IC50 = 4.93 ± 0.45μM
Cell viability (%)
Concentration (M)

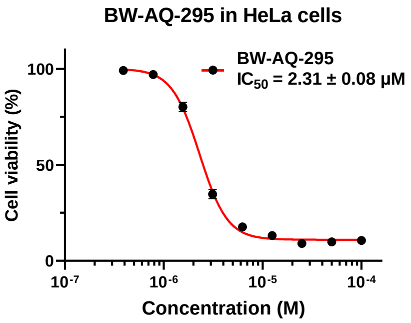
BW-AQ-295 in HeLa cells
BW-AQ-295
IC50 = 2.31 ± 0.08 μM
Cell viability (%)
Concentration (M)

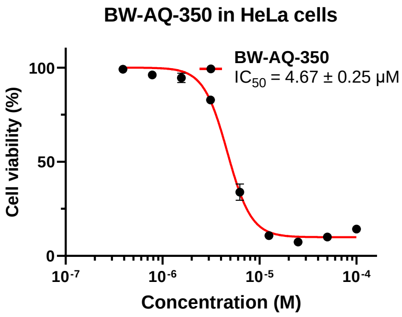
BW-AQ-350 in HeLa cells
BW-AQ-350
IC50 = 4.67 ± 0.25 μM
Cell viability (%)
Concentration (M)

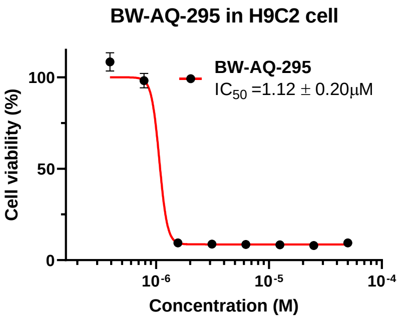
BW-AQ-295 in H9C2 cell
BW-AQ-295
IC50 =1.12 ± 0.20μM
Cell viability (%)
Concentration (M)

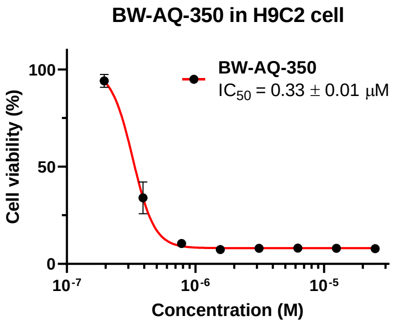
BW-AQ-350 in H9C2 cell
BW-AQ-350
IC50 = 0.33 ± 0.01 μM
Cell viability (%)
Concentration (M)

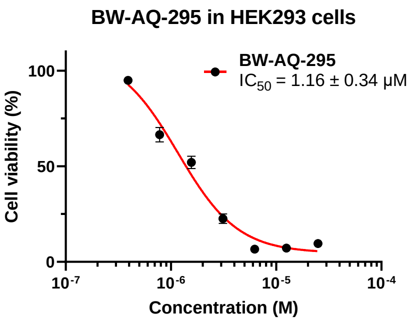
BW-AQ-295 in HEK293 cells
BW-AQ-295
IC50 = 1.16 ± 0.34 μM
Cell viability (%)
Concentration (M)

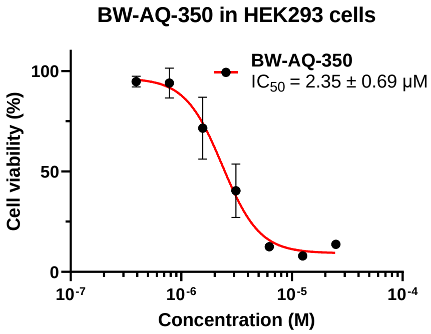
BW-AQ-350 in HEK293 cells
BW-AQ-350
IC50 = 2.35 ± 0.69 μM
Cell viability (%)
Concentration (M)

Figure S2. Relative quantification of Western-blot of EU-1 cells treated with **BW-AQ-295** at the dosage of 0.8 μM. Data is shown as the fold change compared to the control group (time = 0) after normalization by GAPDH.

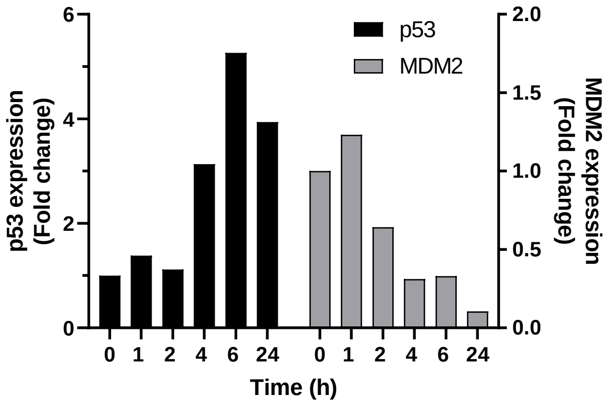

Figure S3. Relative quantification of Western-blot of EU-1 cells treated with **BW-AQ-350** at the dosage of 1 μM. Data is shown as sfold change compared to the control group (time = 0) after normalization by GAPDH.

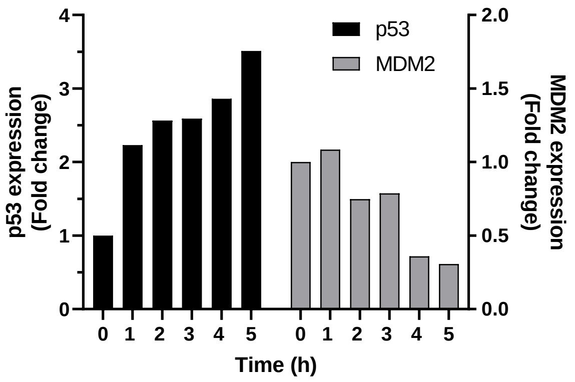

Figure S4. HMBC 2D-NMR of **BW-AQ-336**.

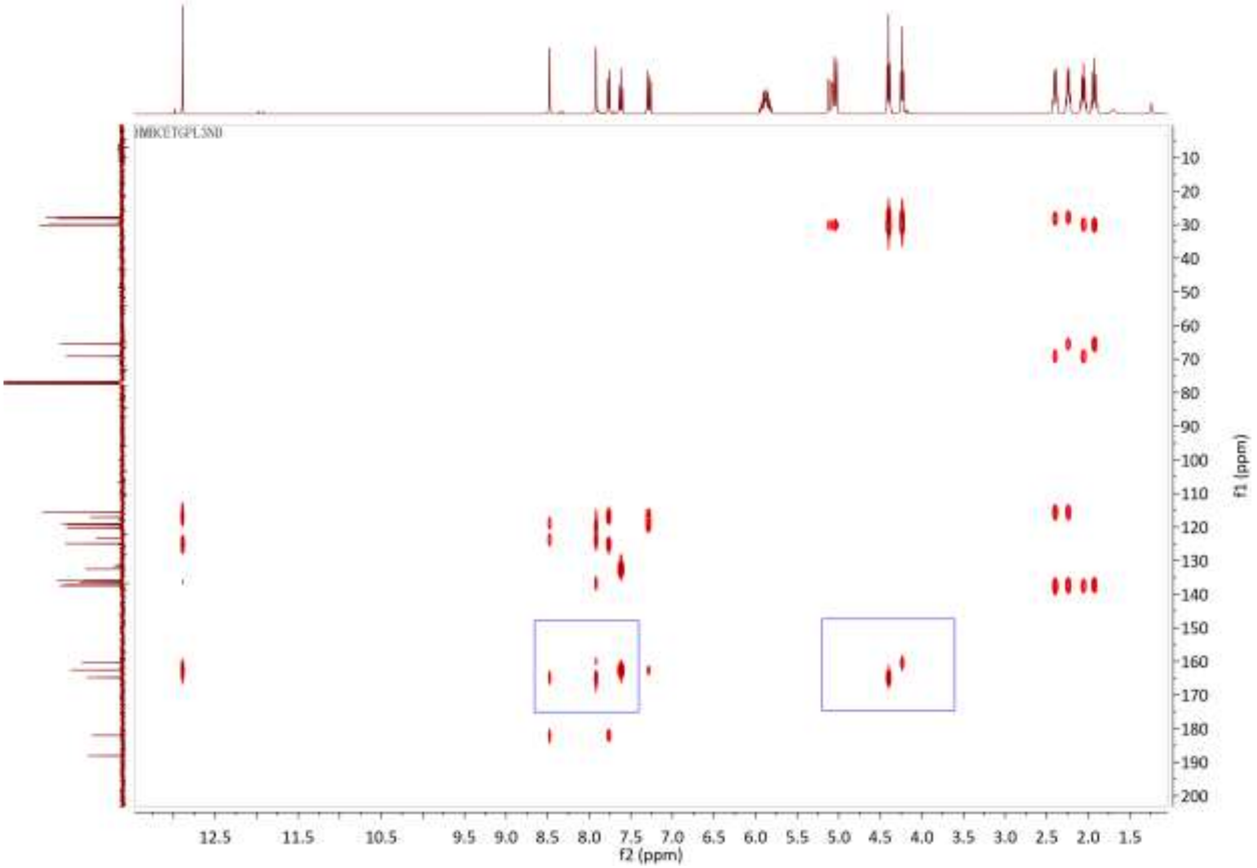

Figure S5. Time plot of NAC-reactivity of the AQ analogs at 37 °C in PBS.

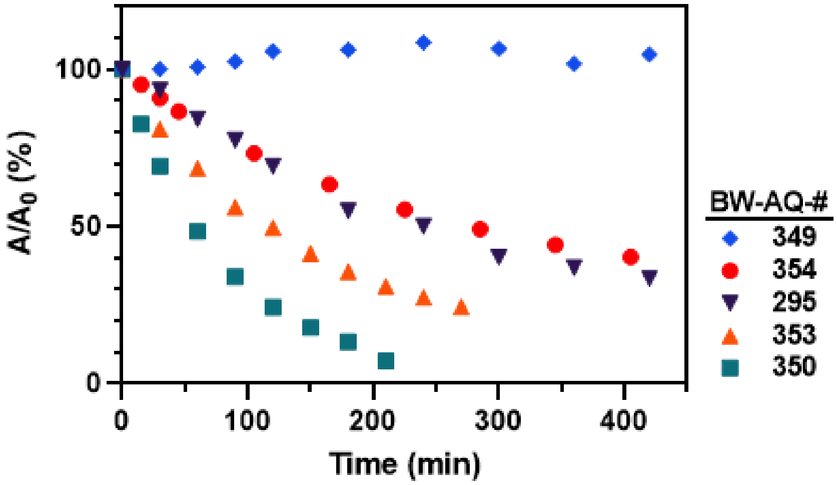

NMR spectra:

Compound **1a:**

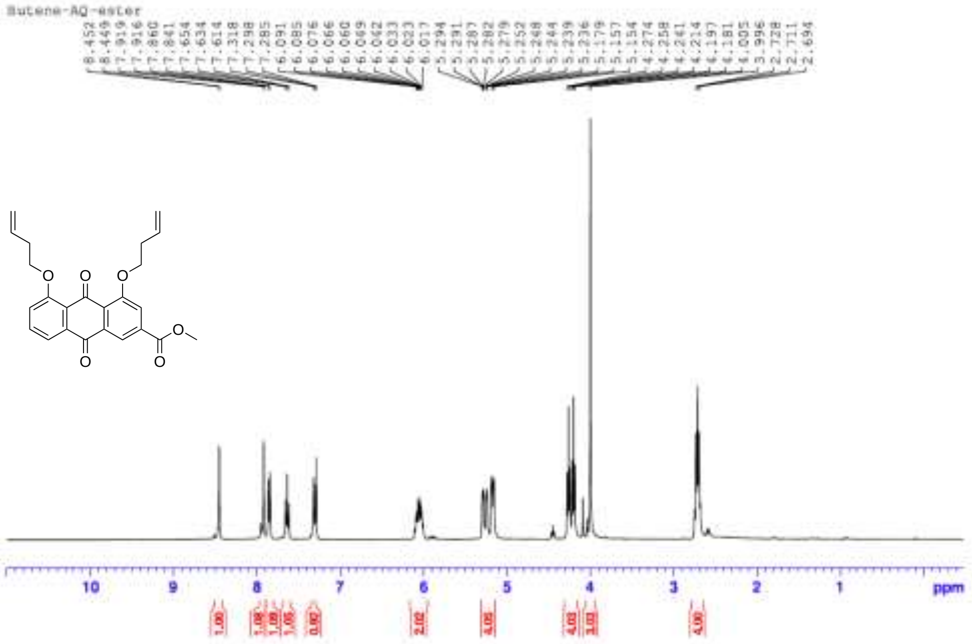

Compound **1a:**

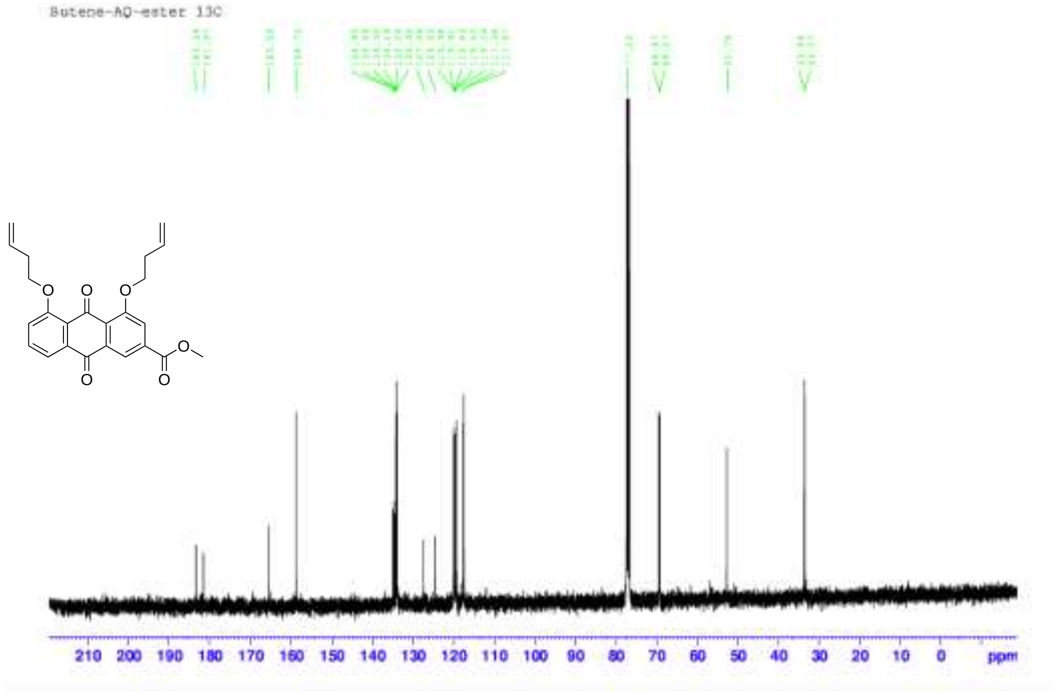

Compound **2a:**

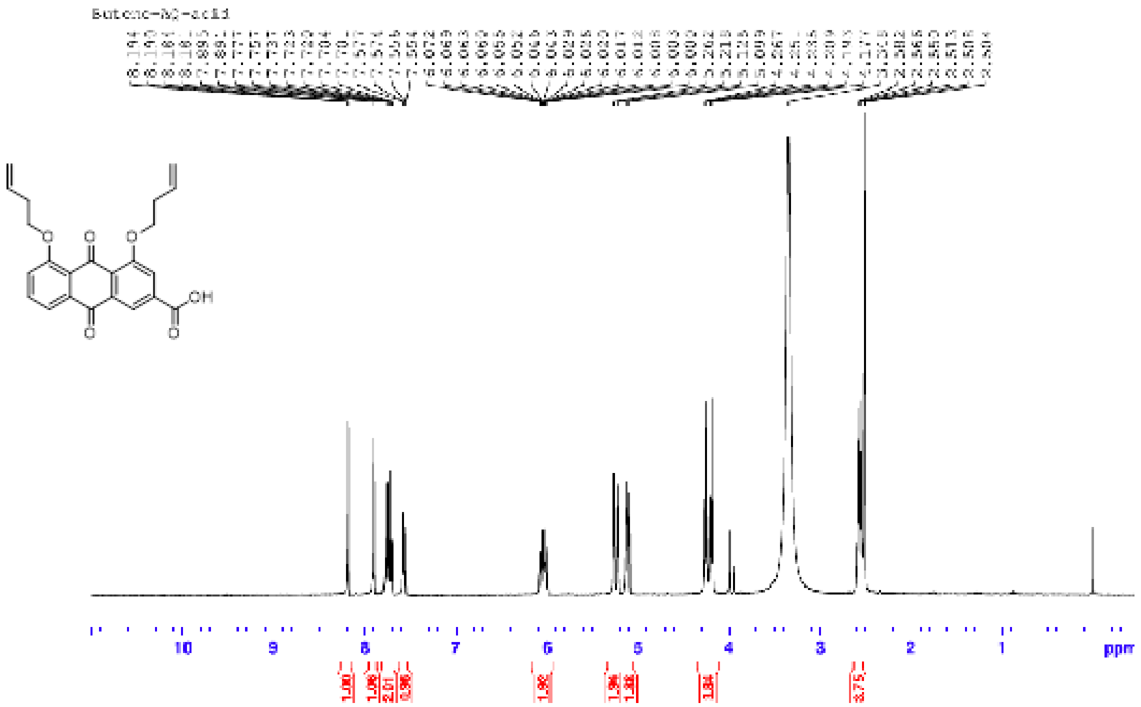

Compound **2a:**

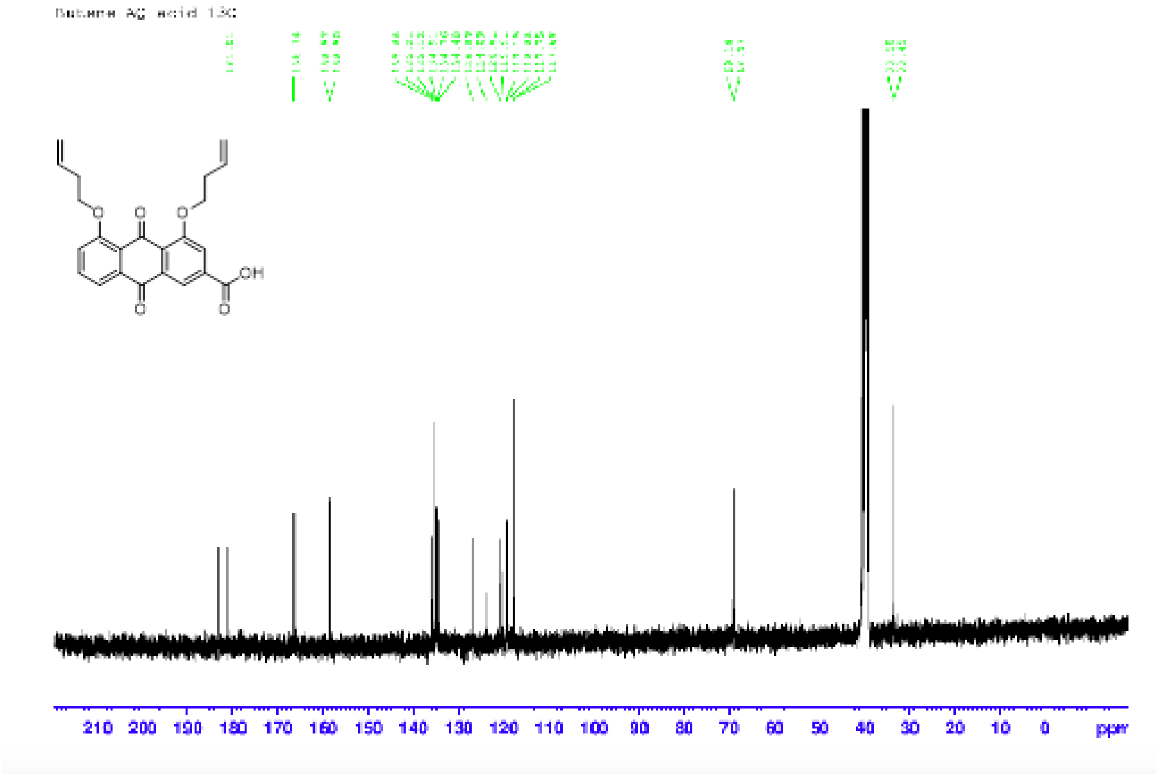

Compound **3a:**

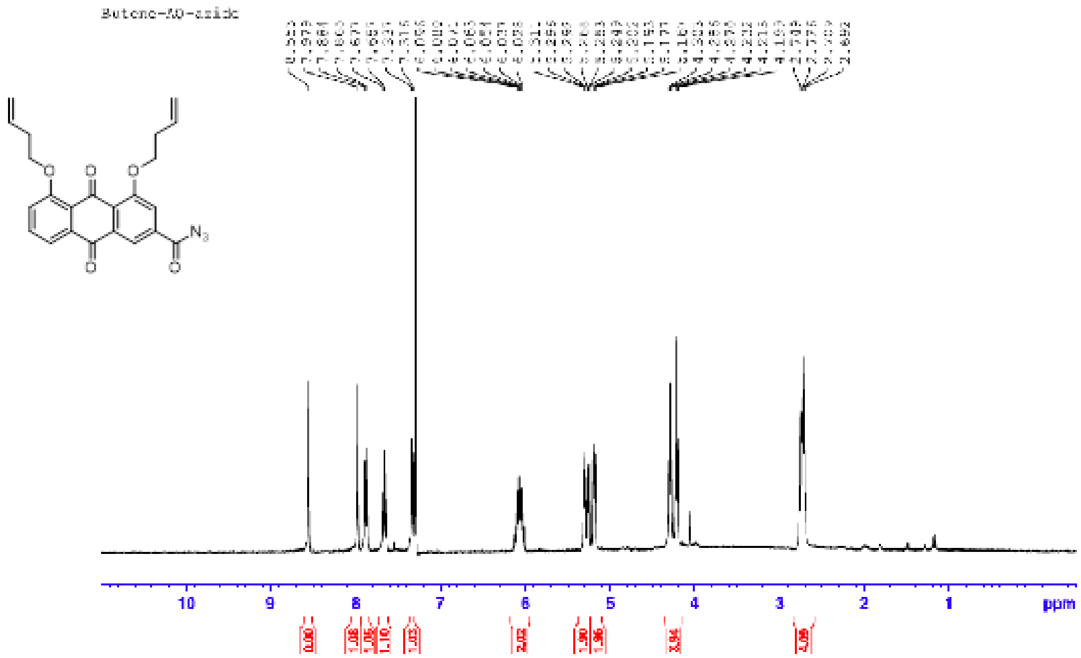

Compound **3a:**

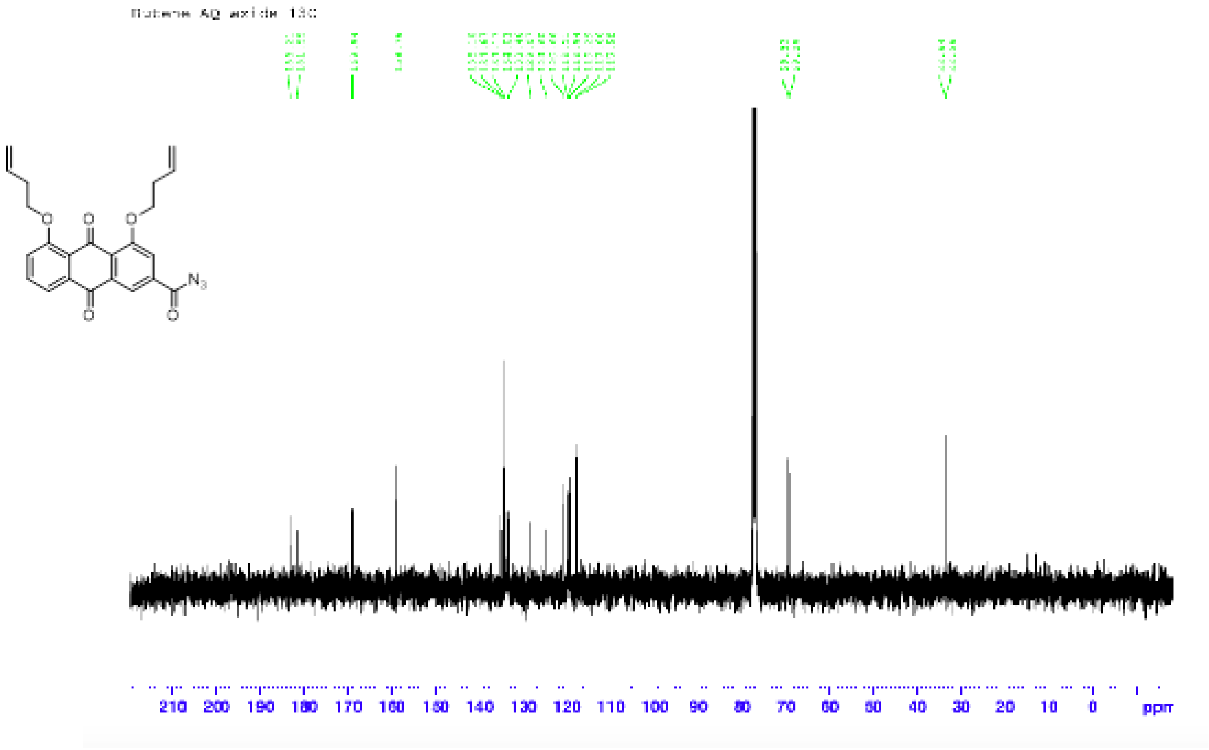

Compound **4a:**

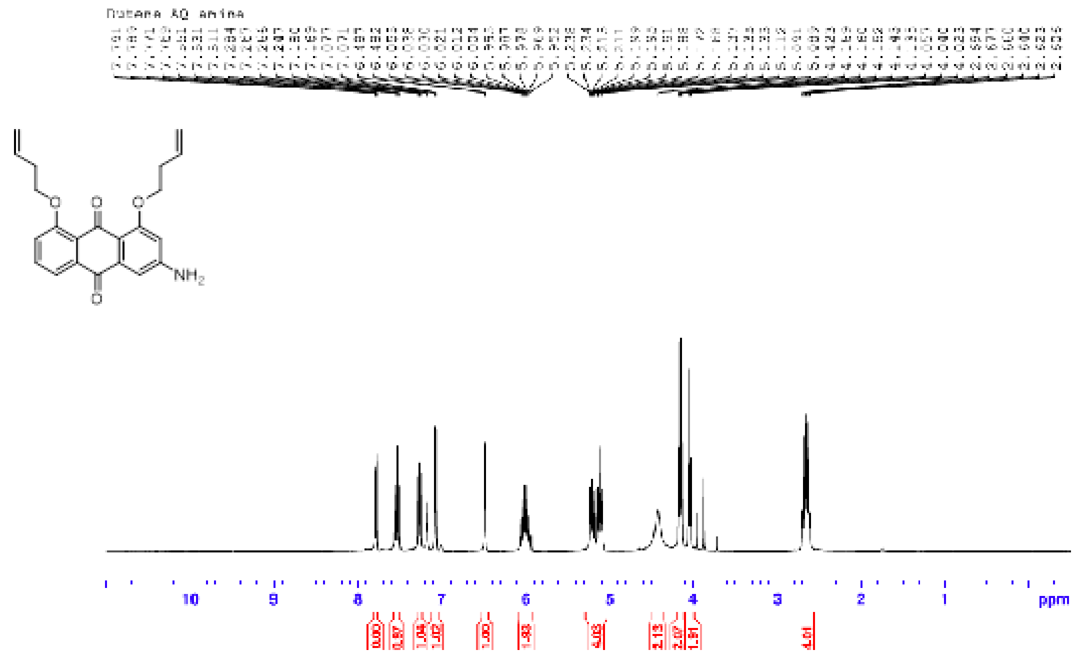

Compound **4a:**

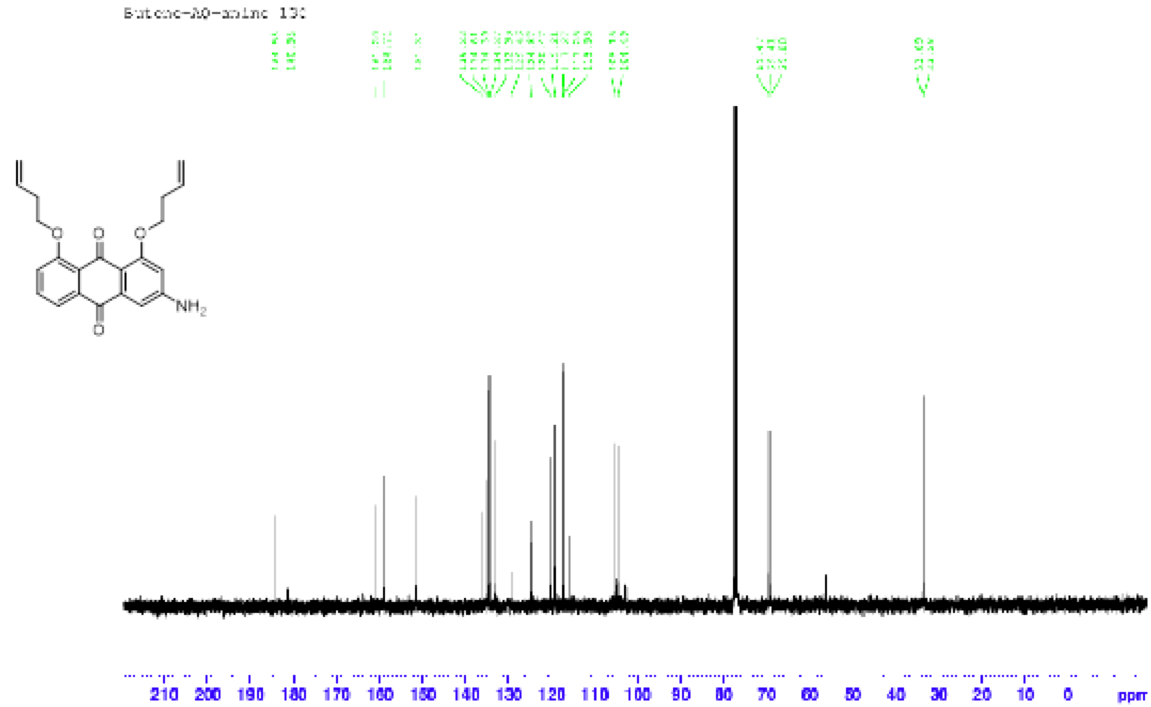

Compound **5a:**

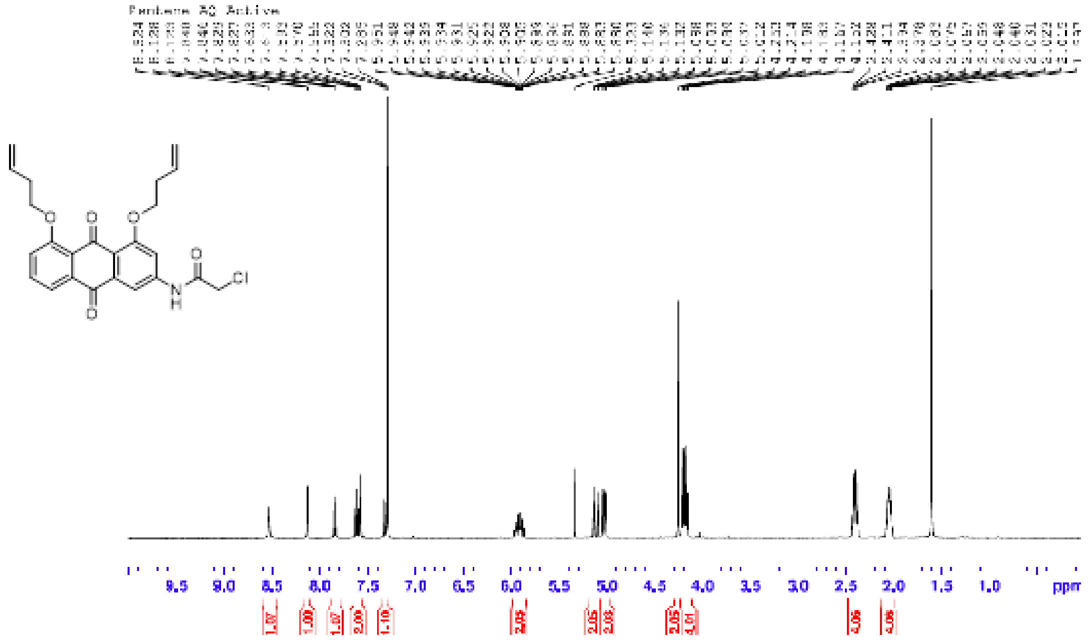

Compound **5a:**

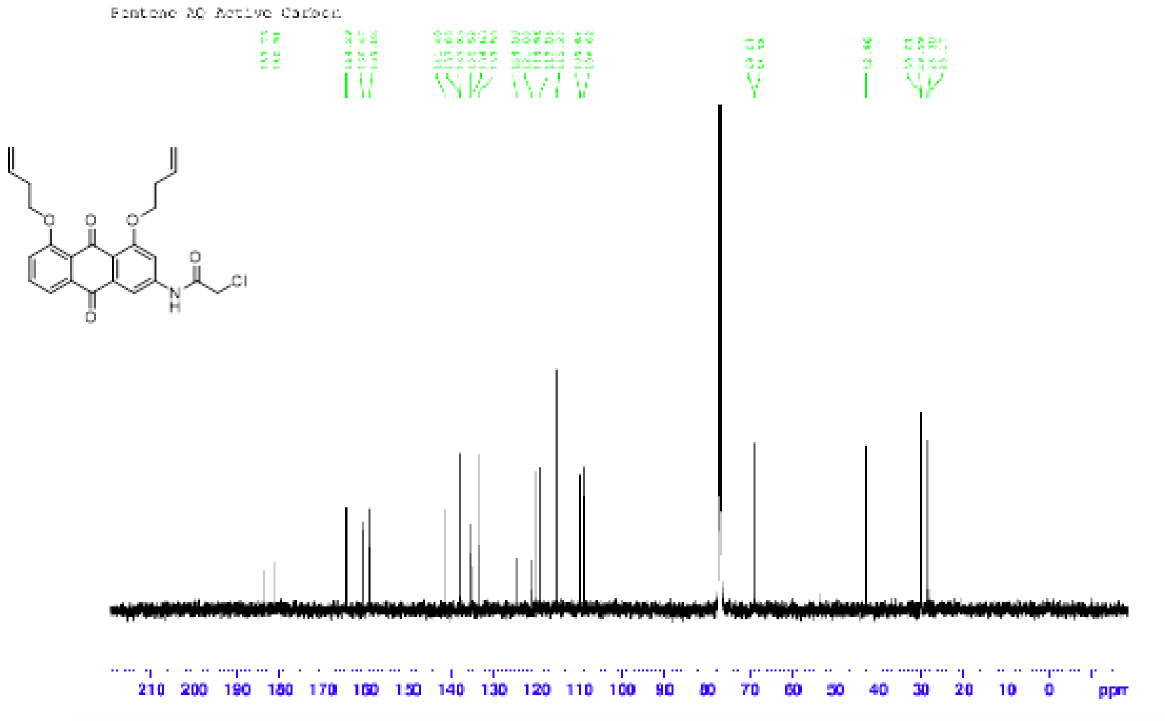

BW-AQ-260:

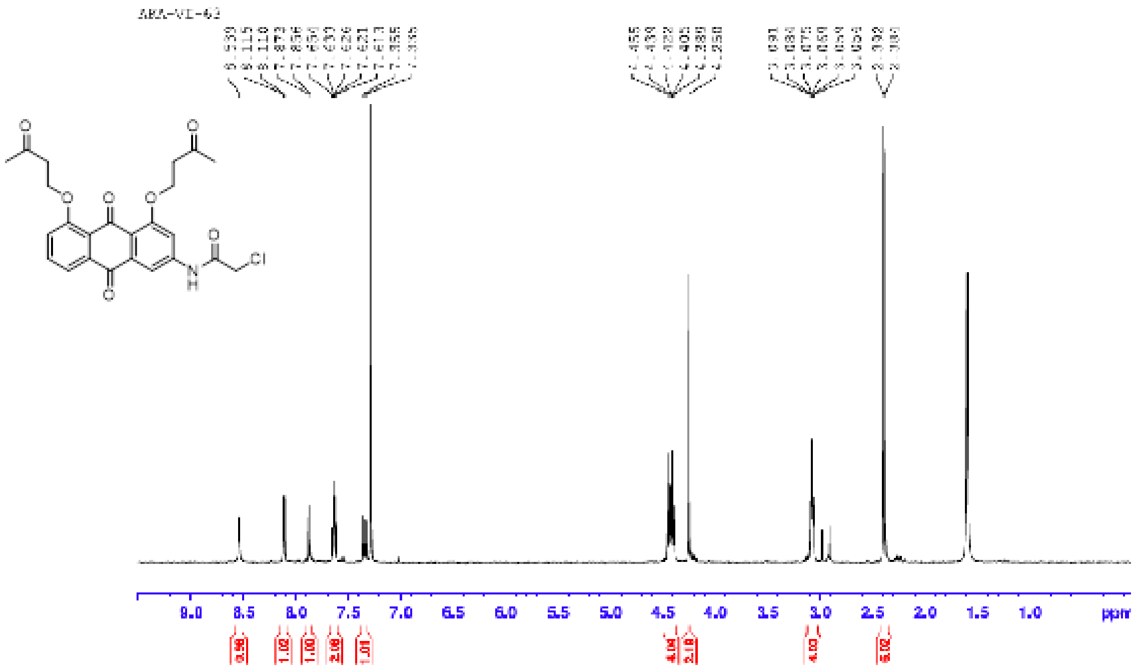

BW-AQ-260:

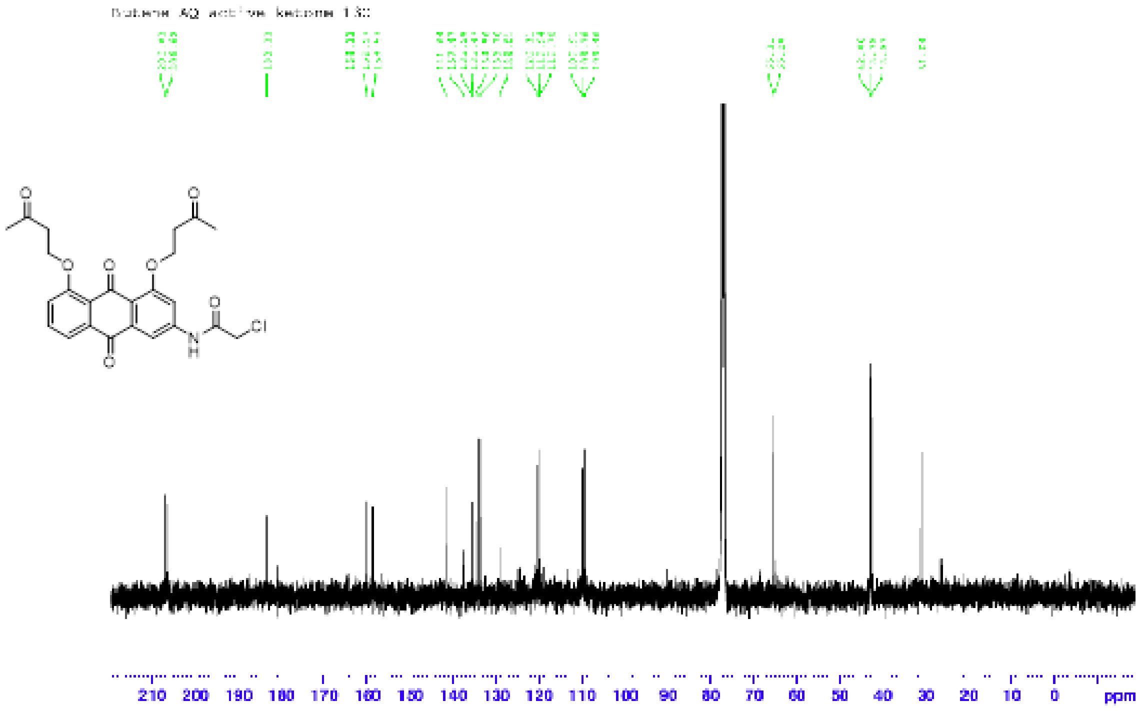

Compound 1**b:**

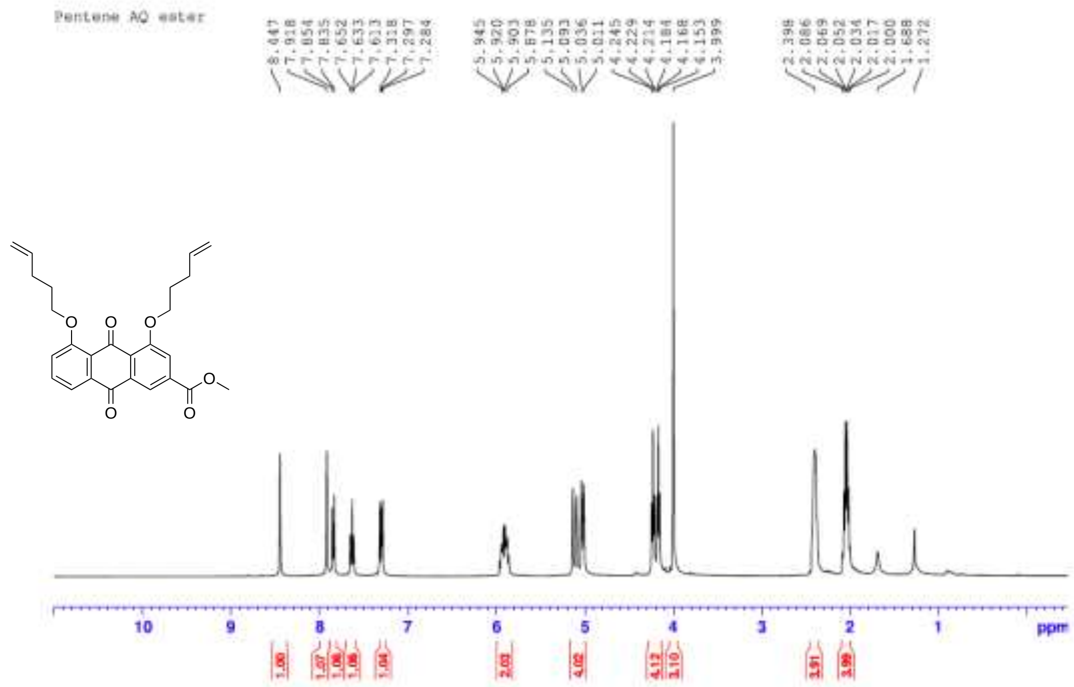

Compound 1**b:**

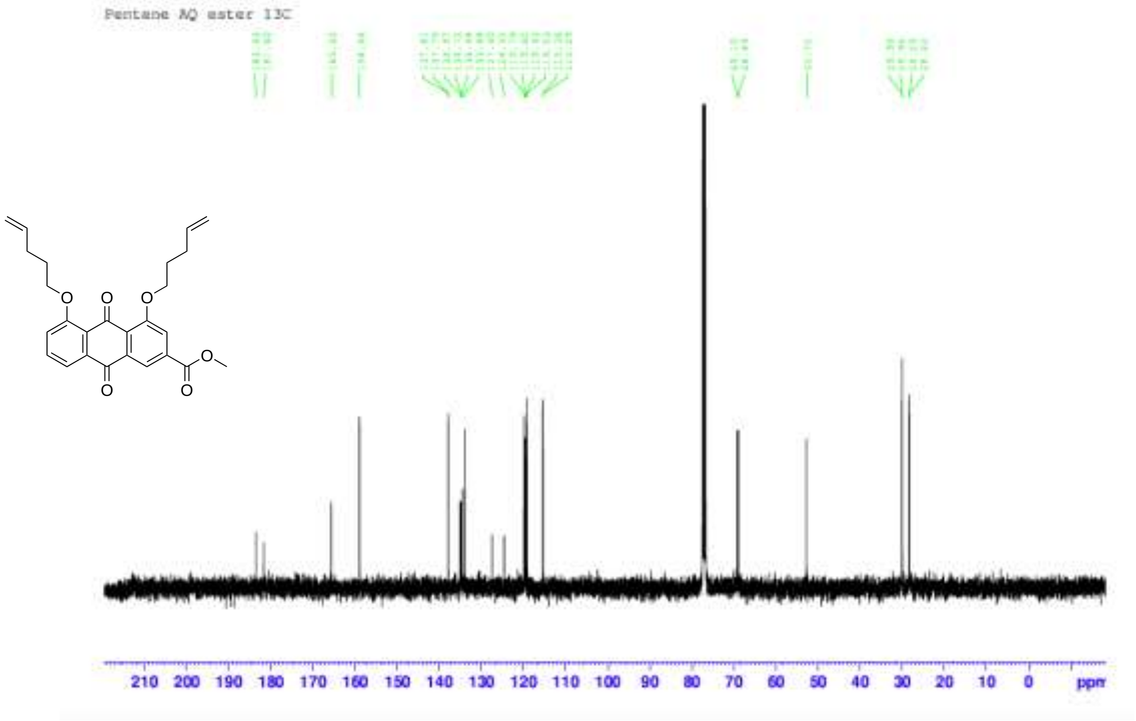

Compound 2**b:**

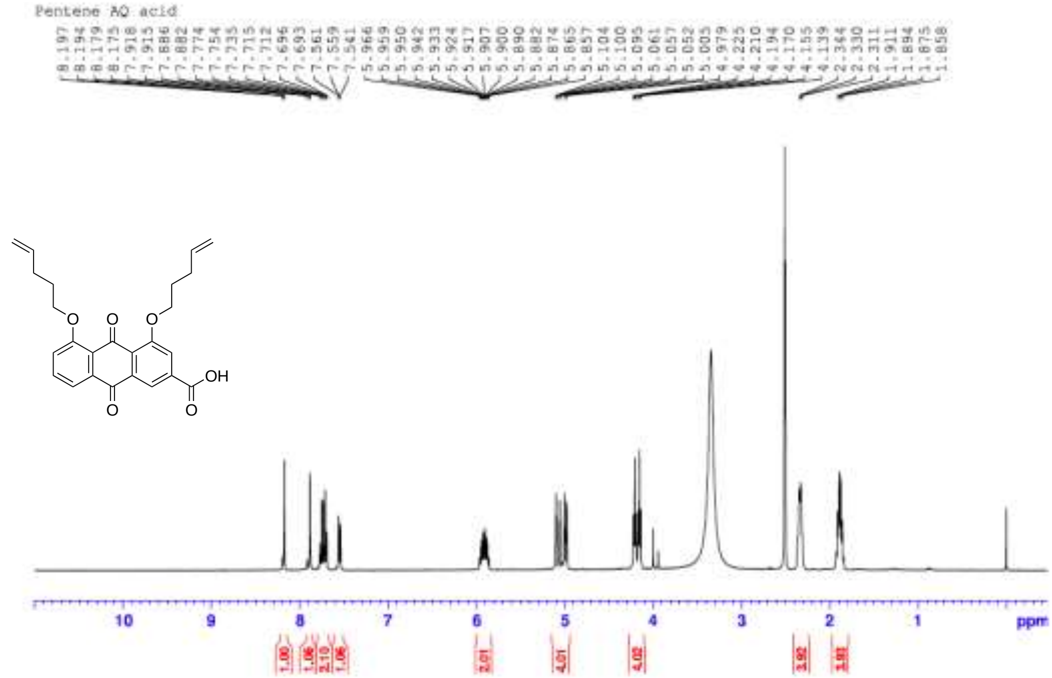

Compound 2**b:**

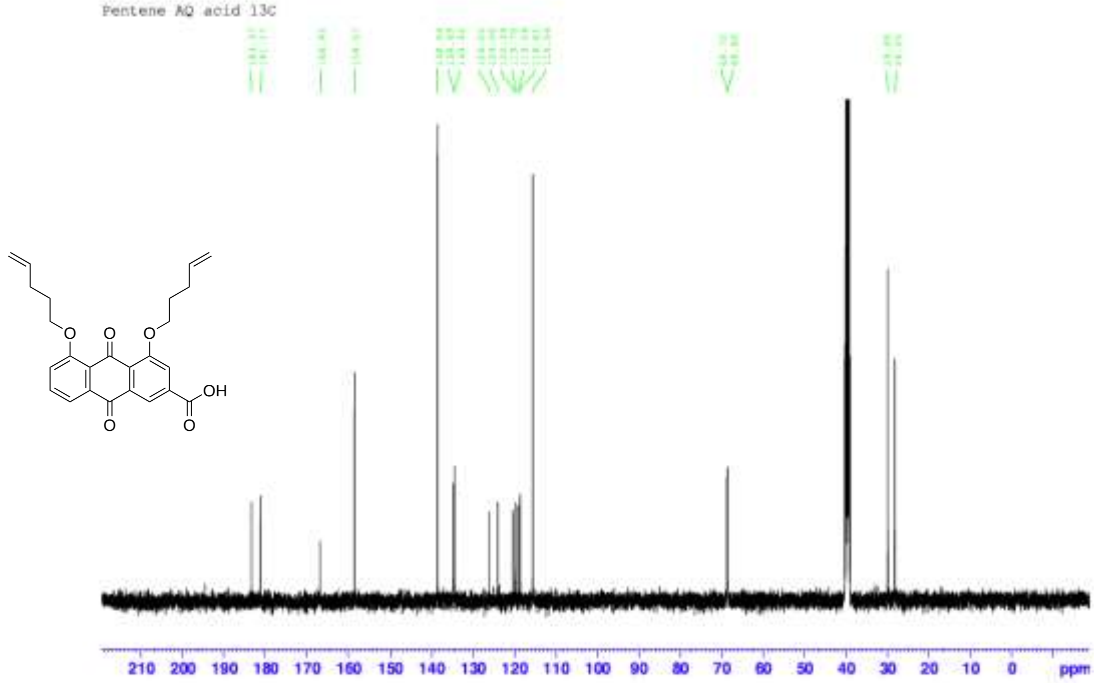

Compound **3b:**

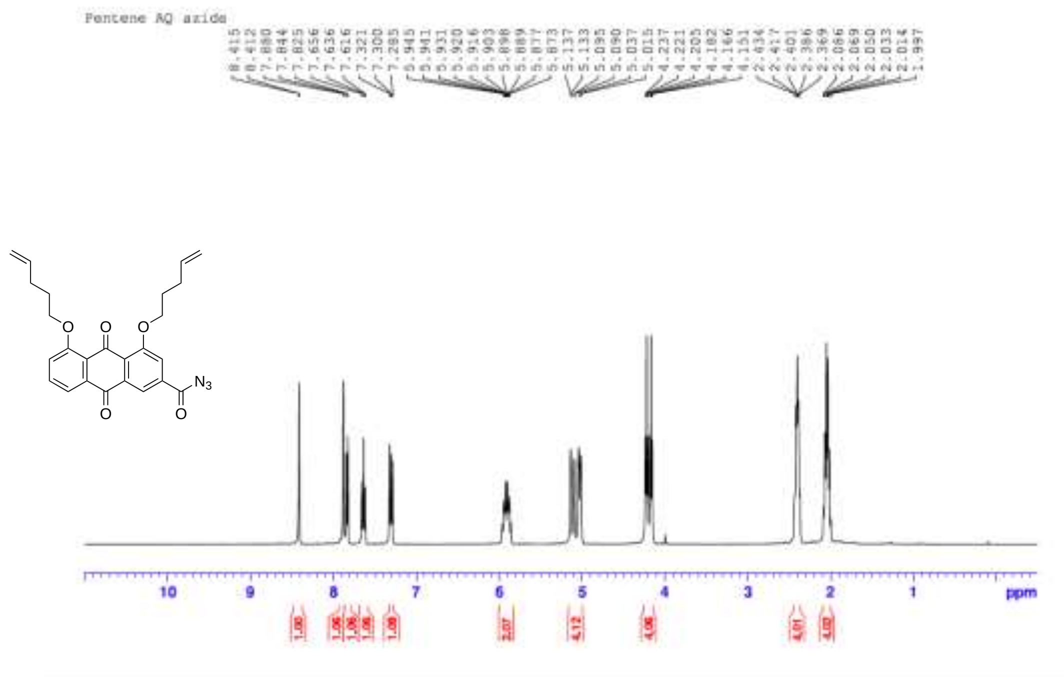

Compound **3b:**

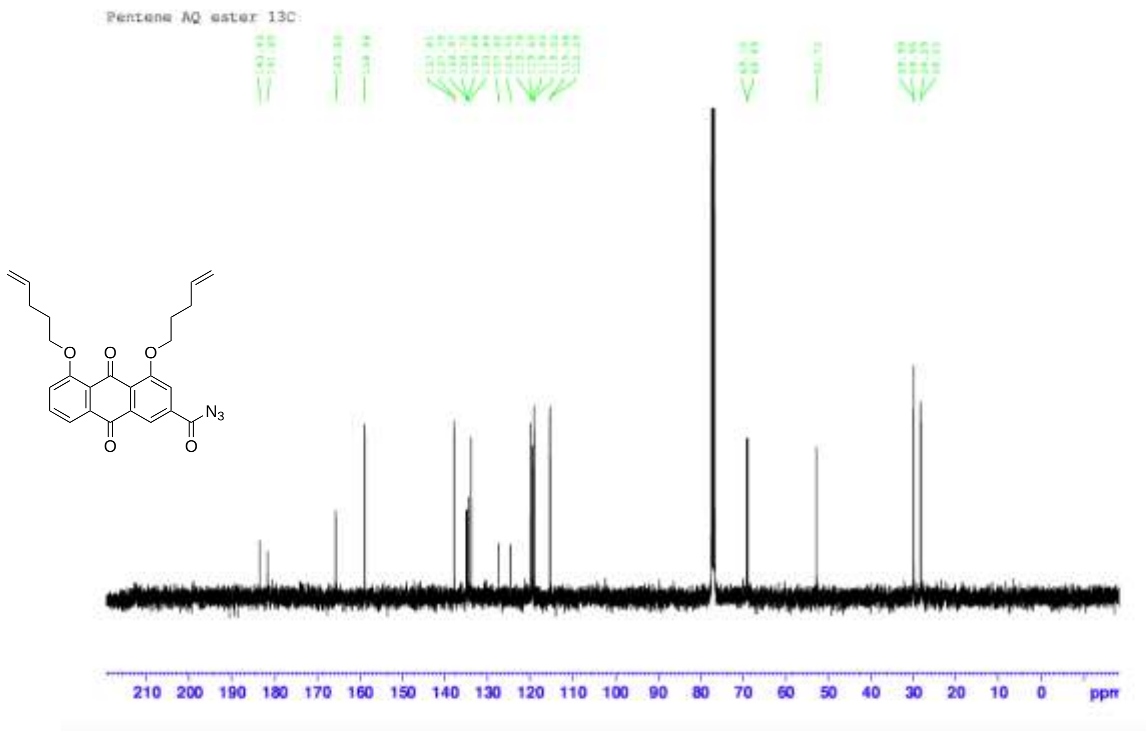

Information Classification: General

Compound **4b:**

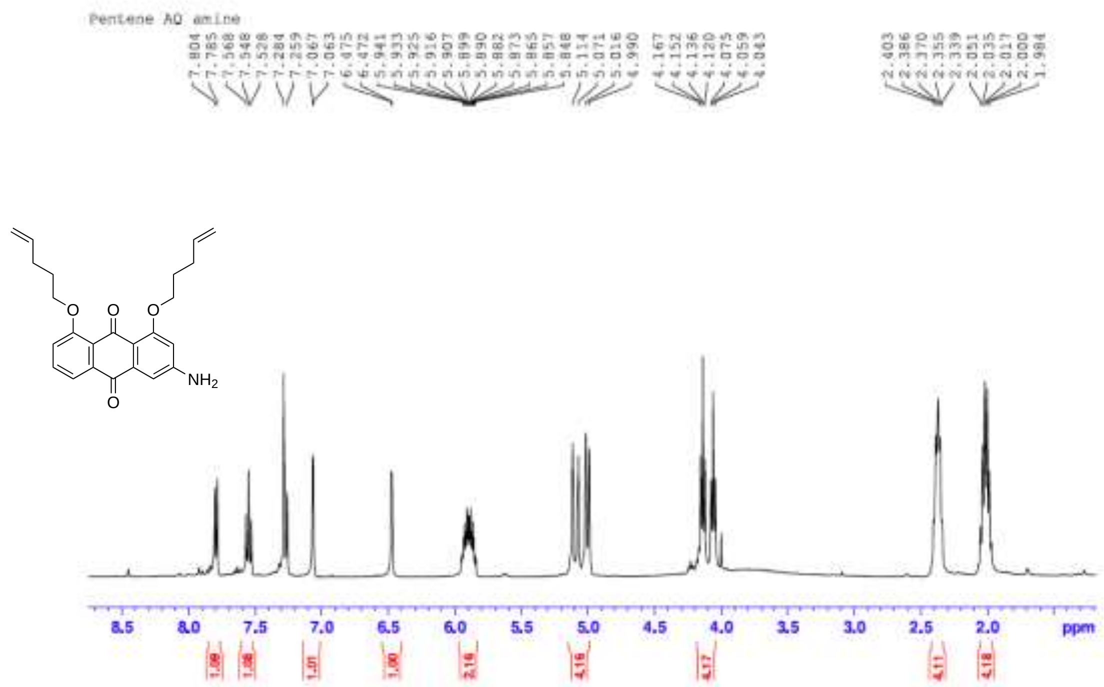

Compound **4b**

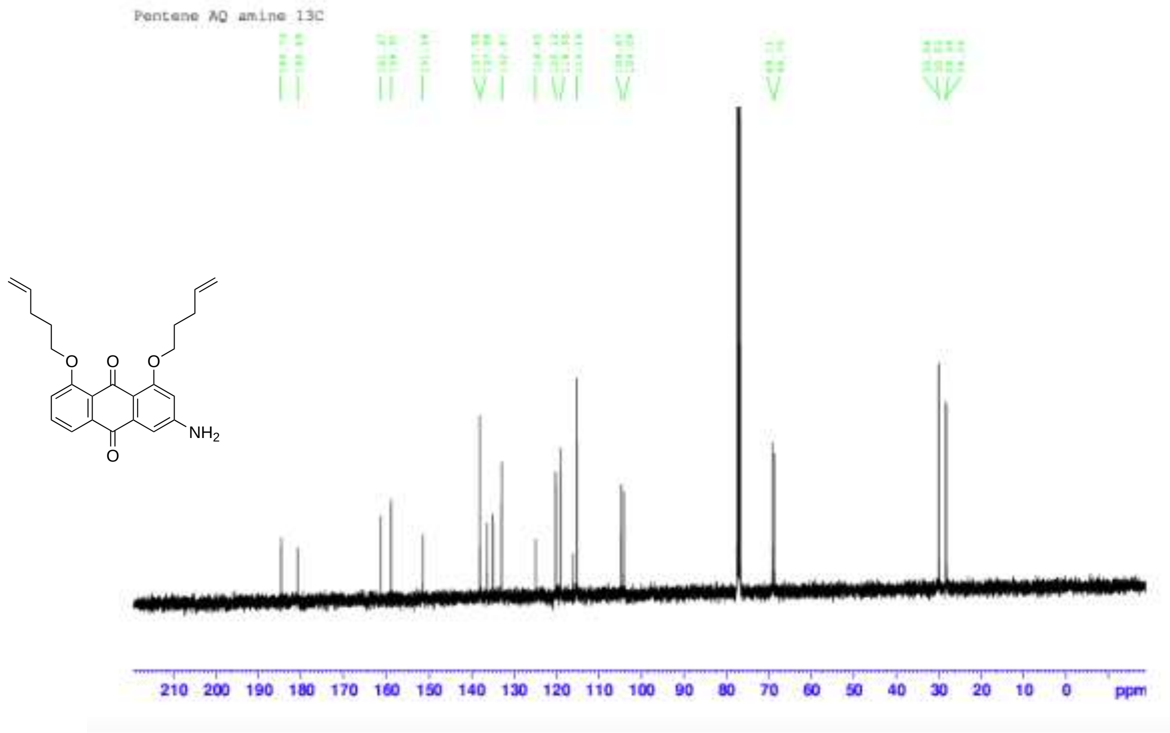

Compound **5b:**

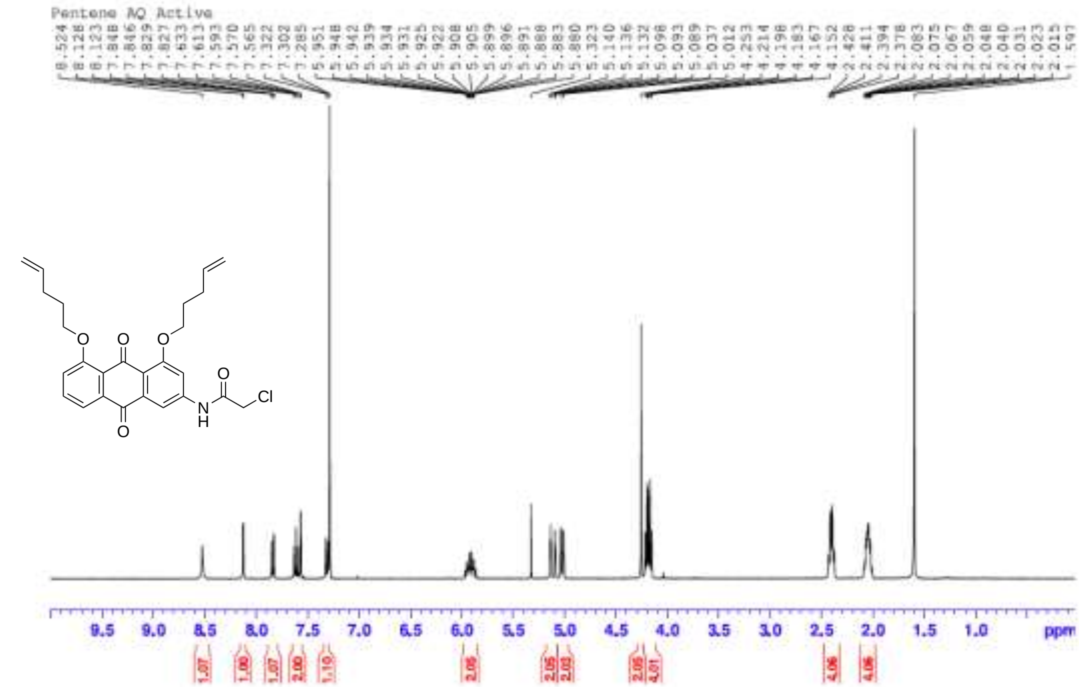

Compound **5b:**

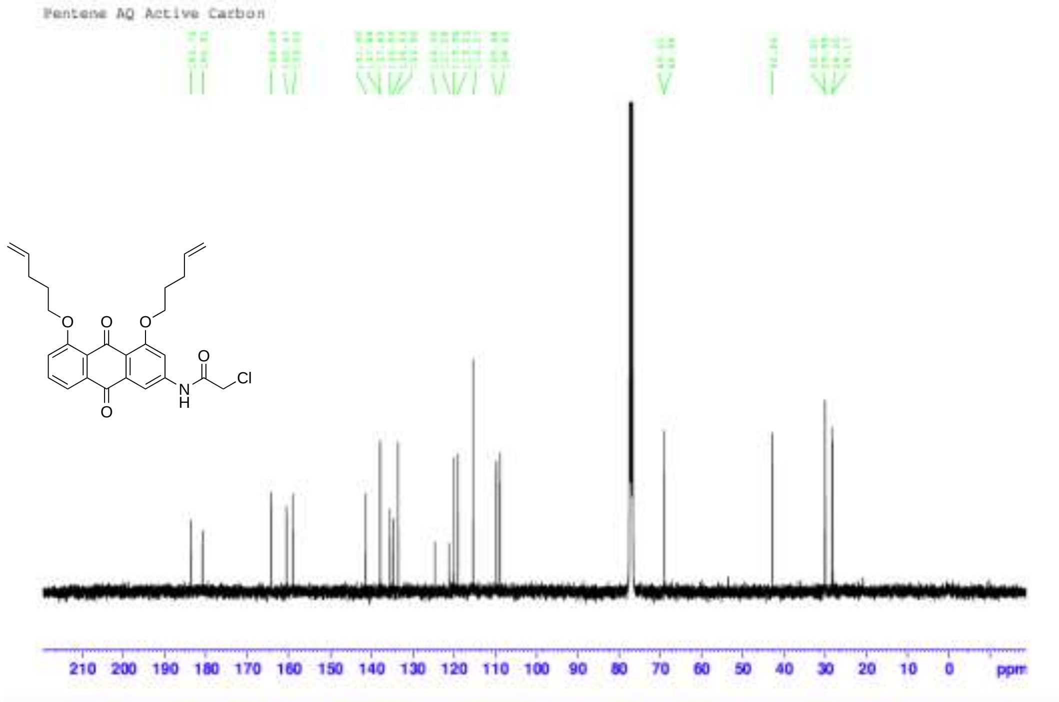

BW-AQ-295:

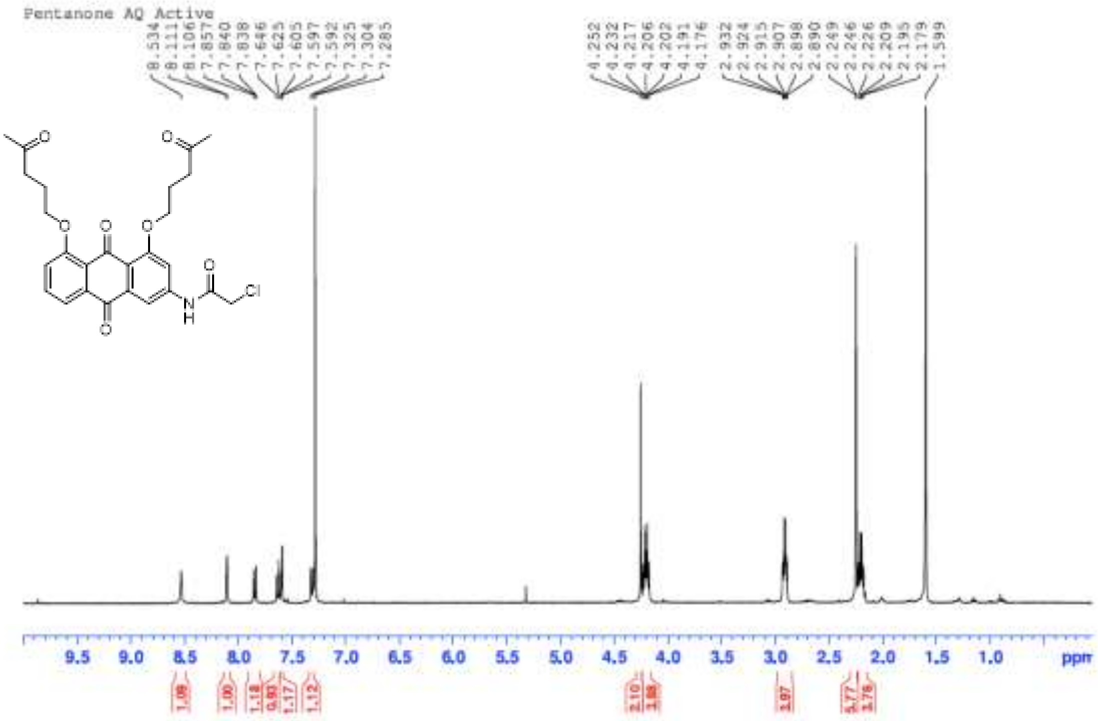

BW-AQ-295:

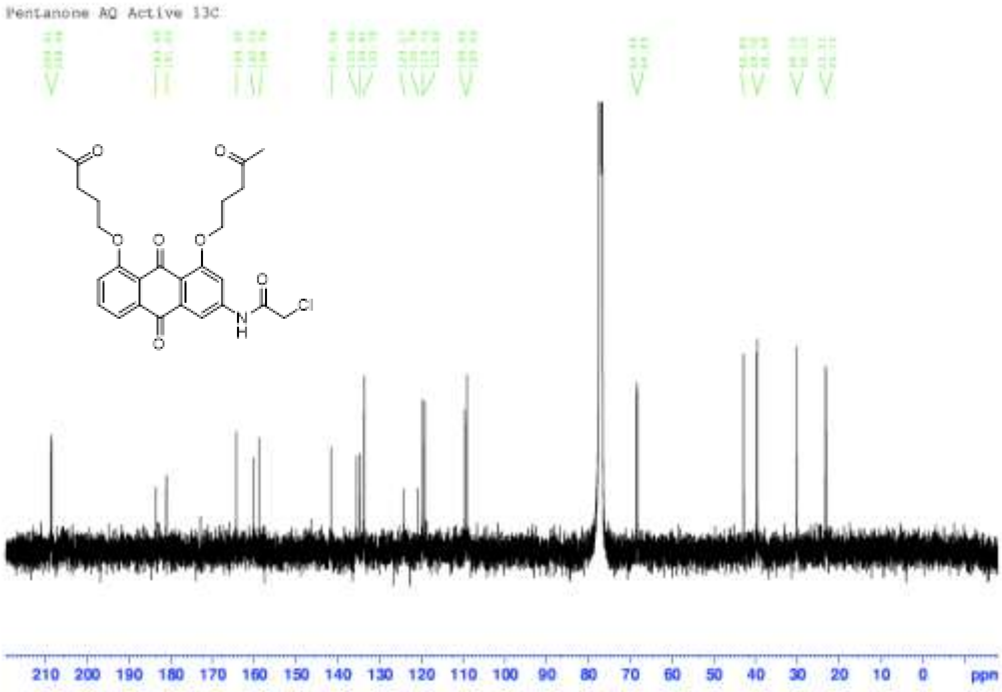

Compound **1c:**

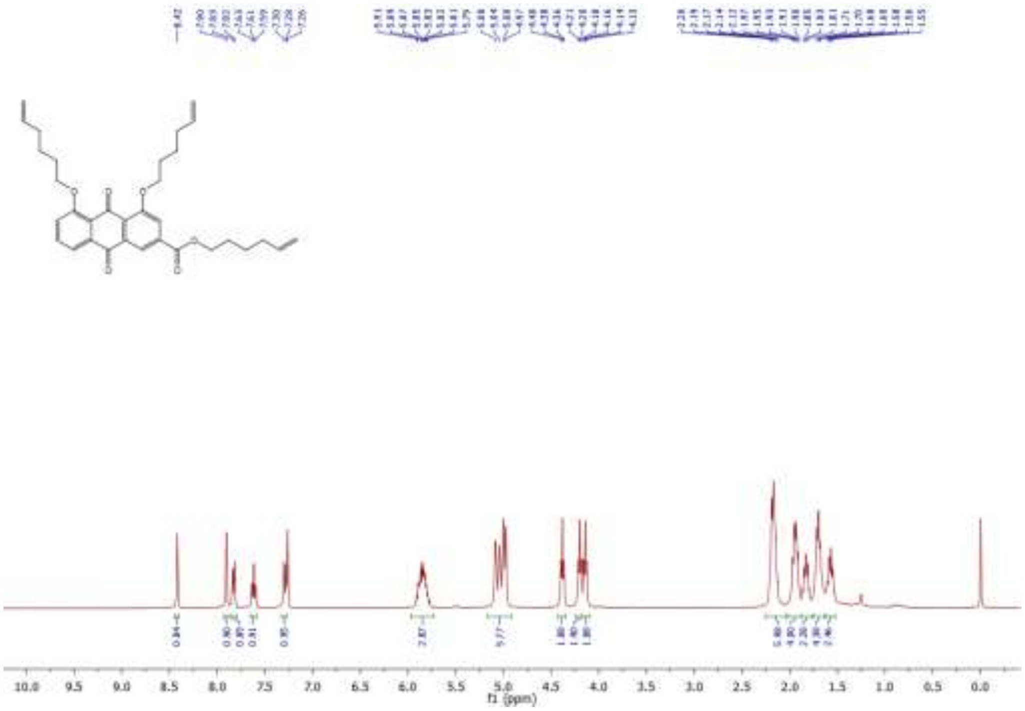

Compound **1c:**

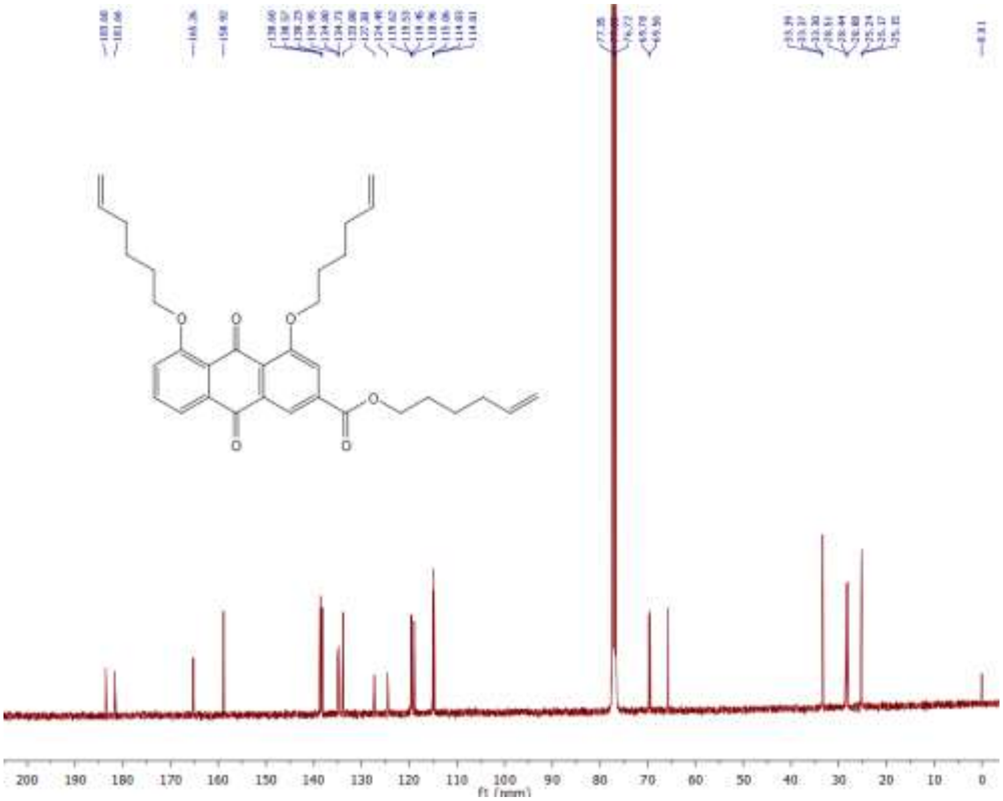

Compound **2c:**

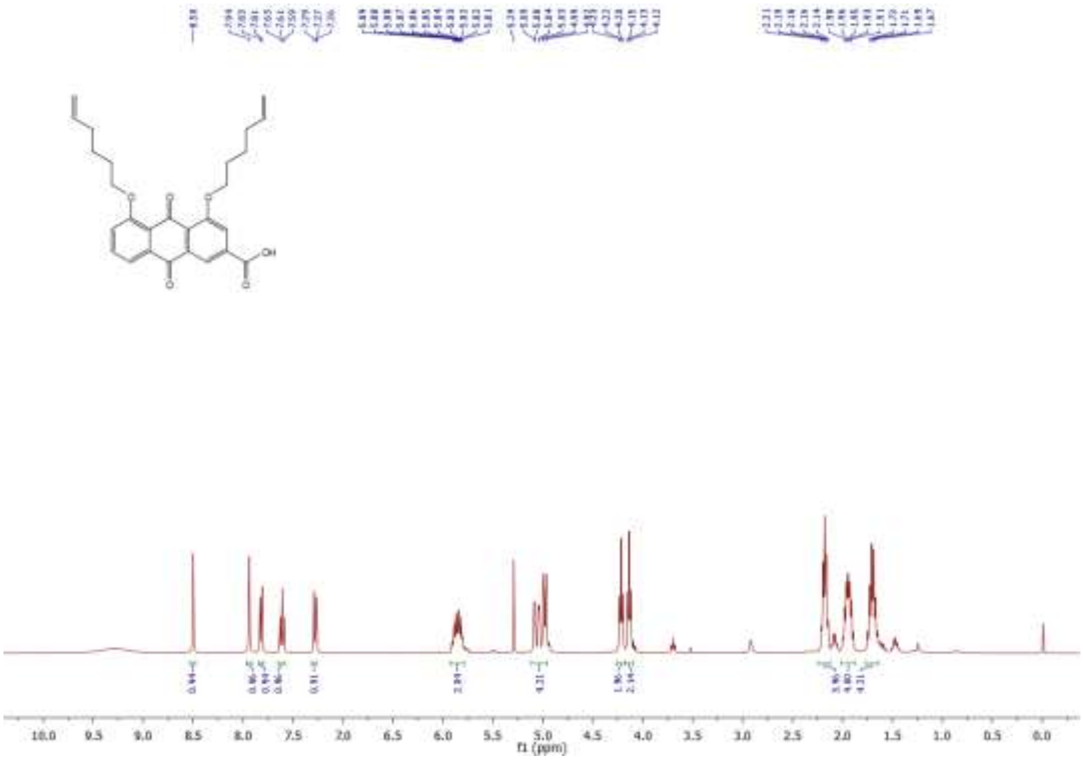

Compound **2c:**

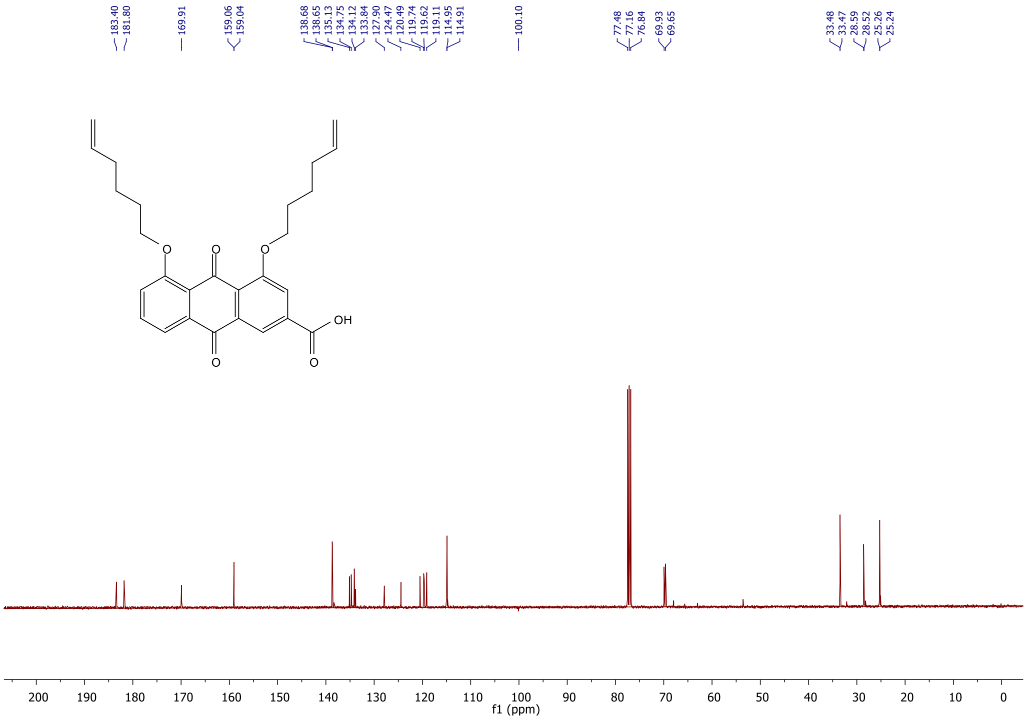

Compound **3c:**

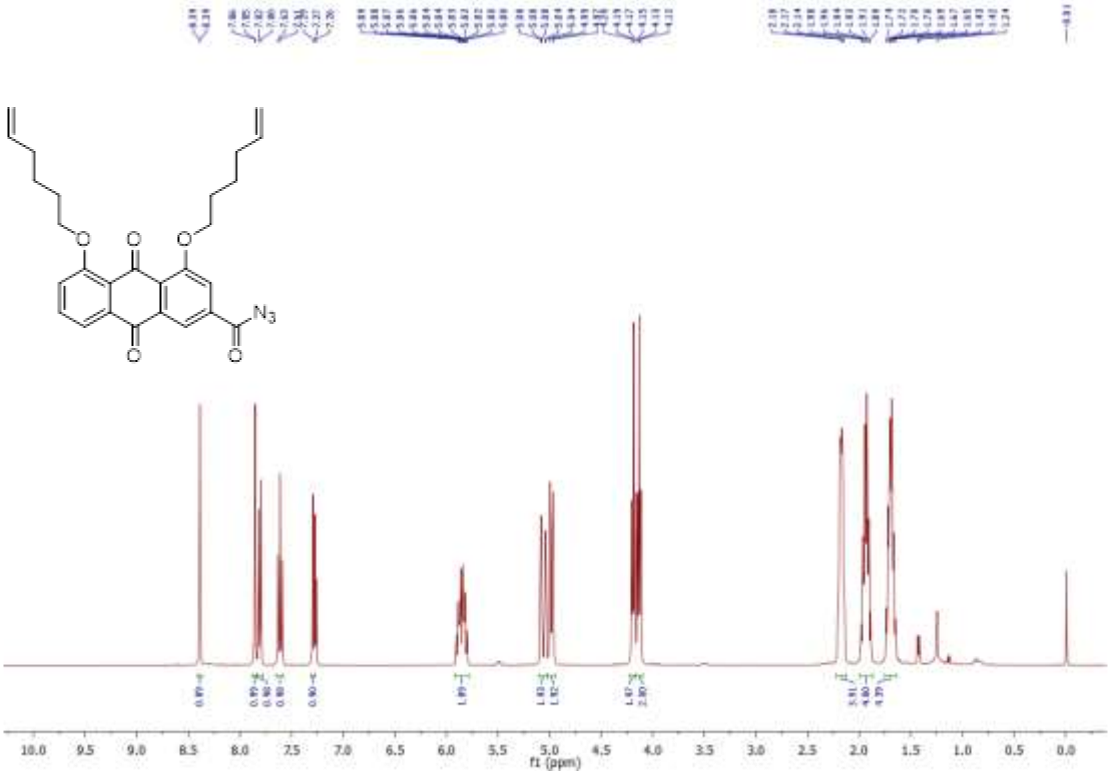

Compound **3c:**

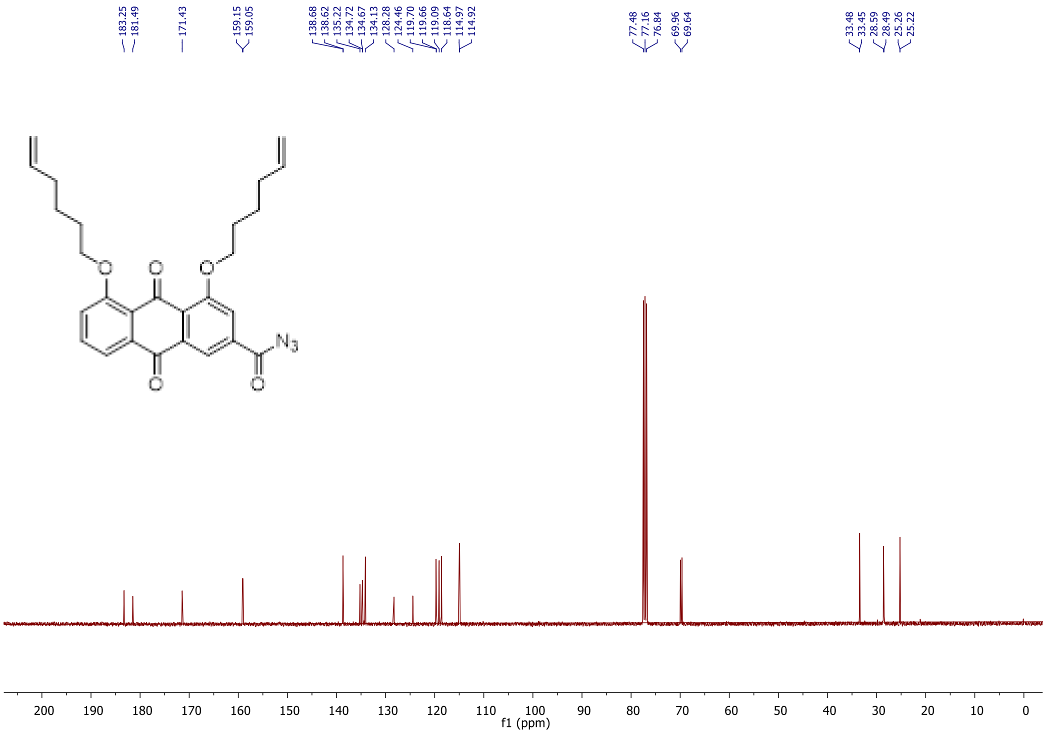

Compound **4c:**

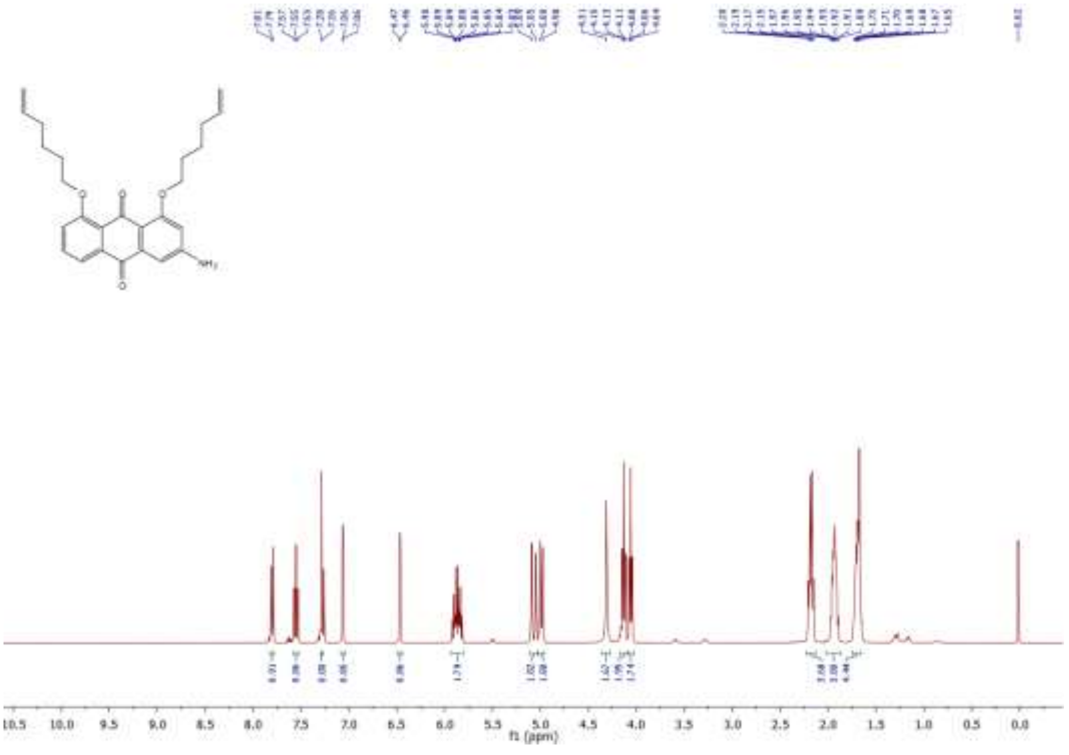

Compound **4c:**

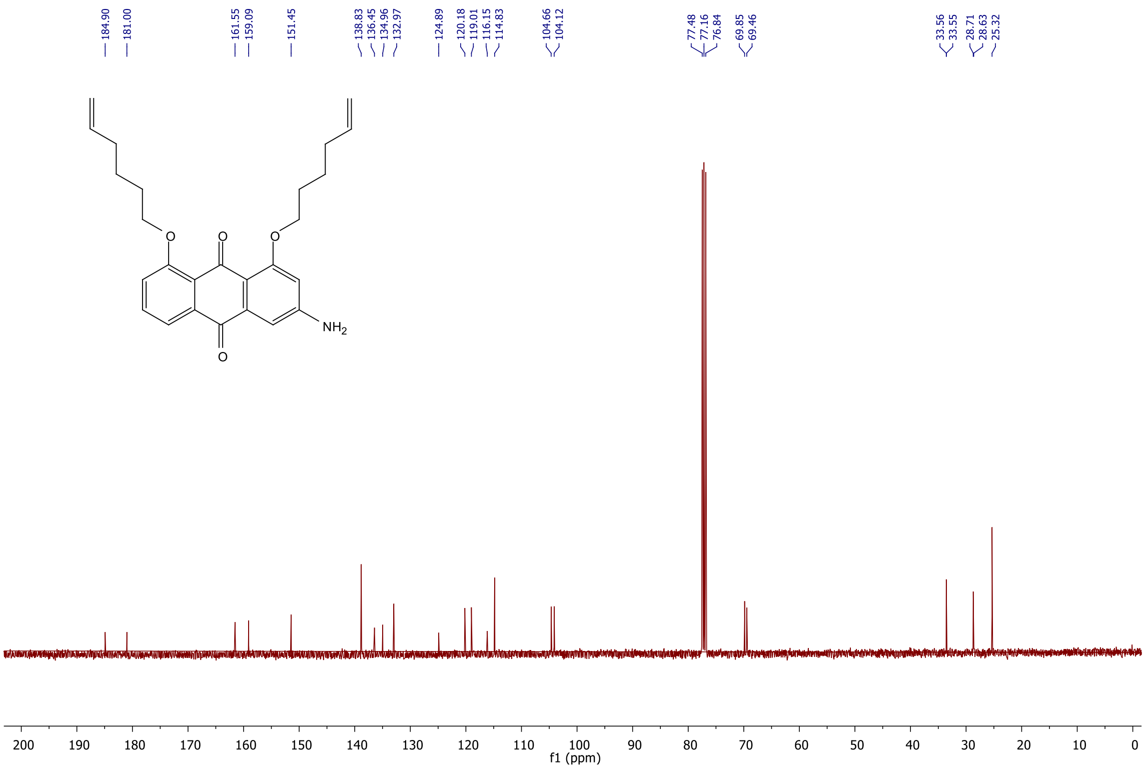

Compound **5c:**

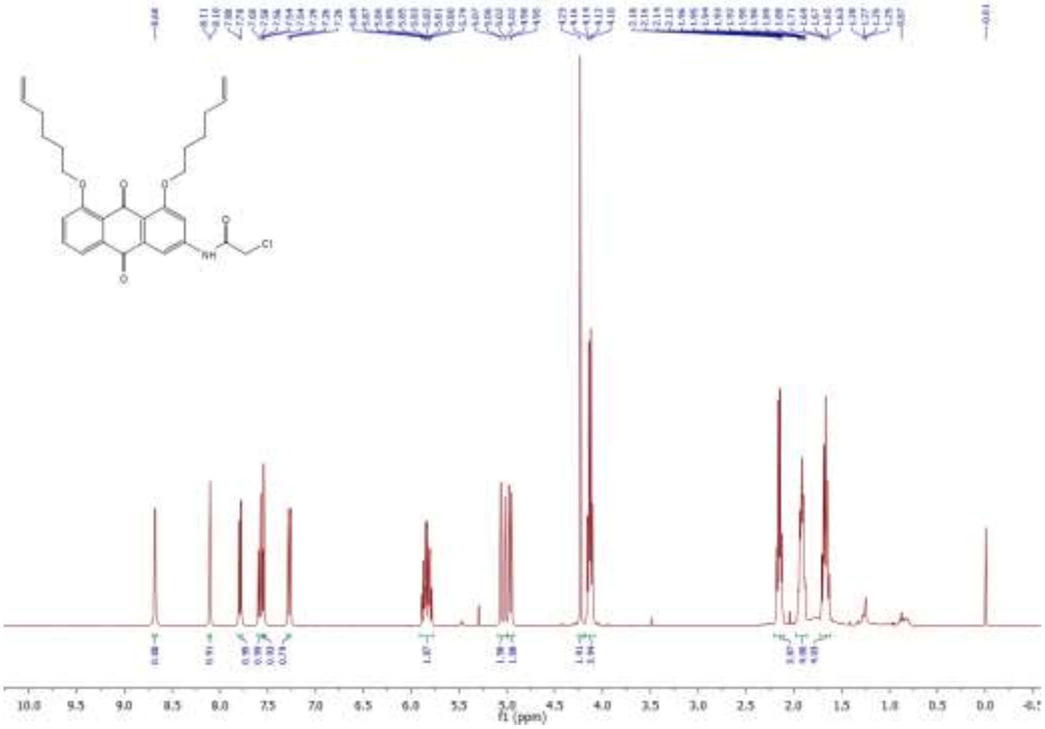

Compound **5c:**

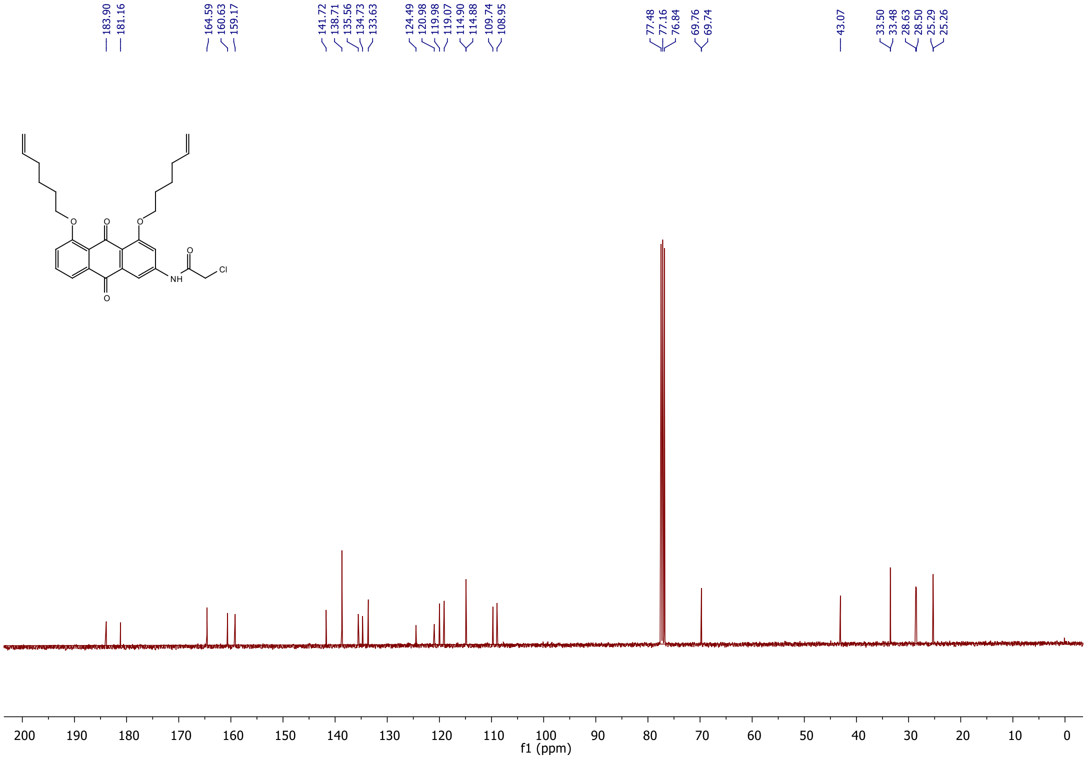

BW-AQ-345:

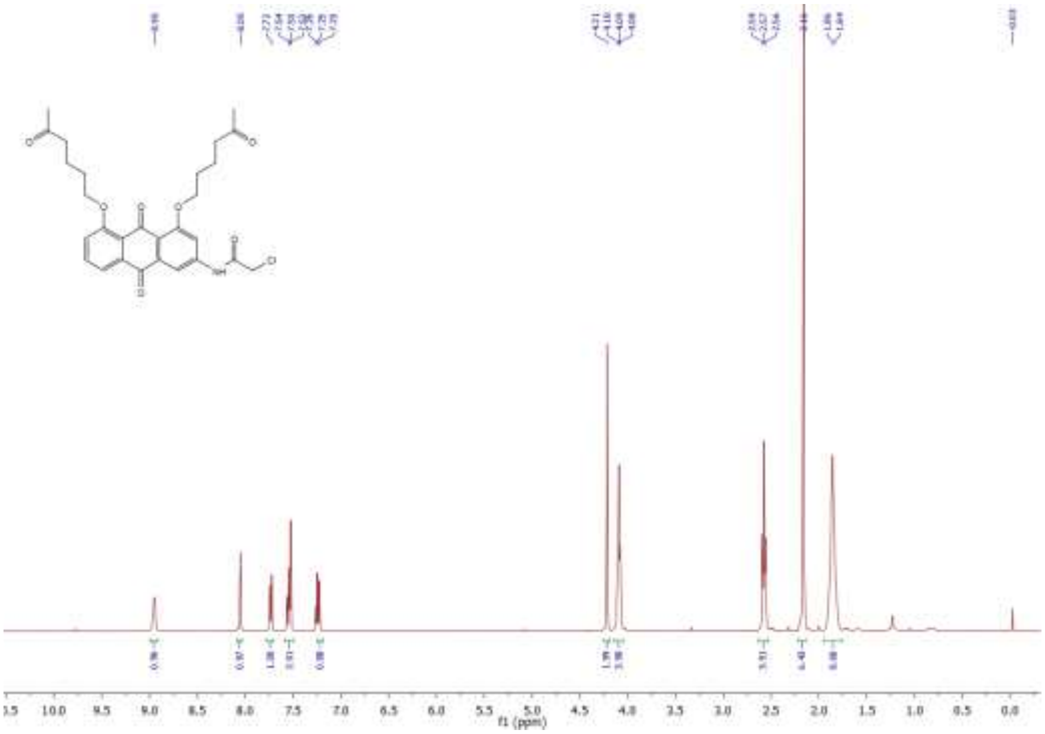

BW-AQ-345:

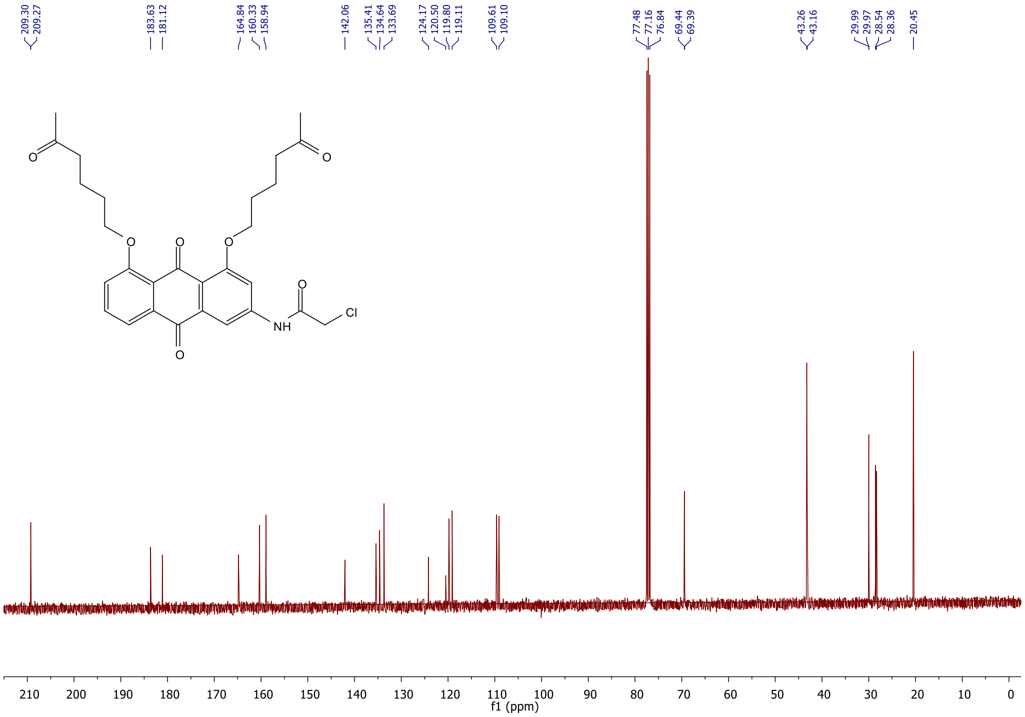

Compound **6**:

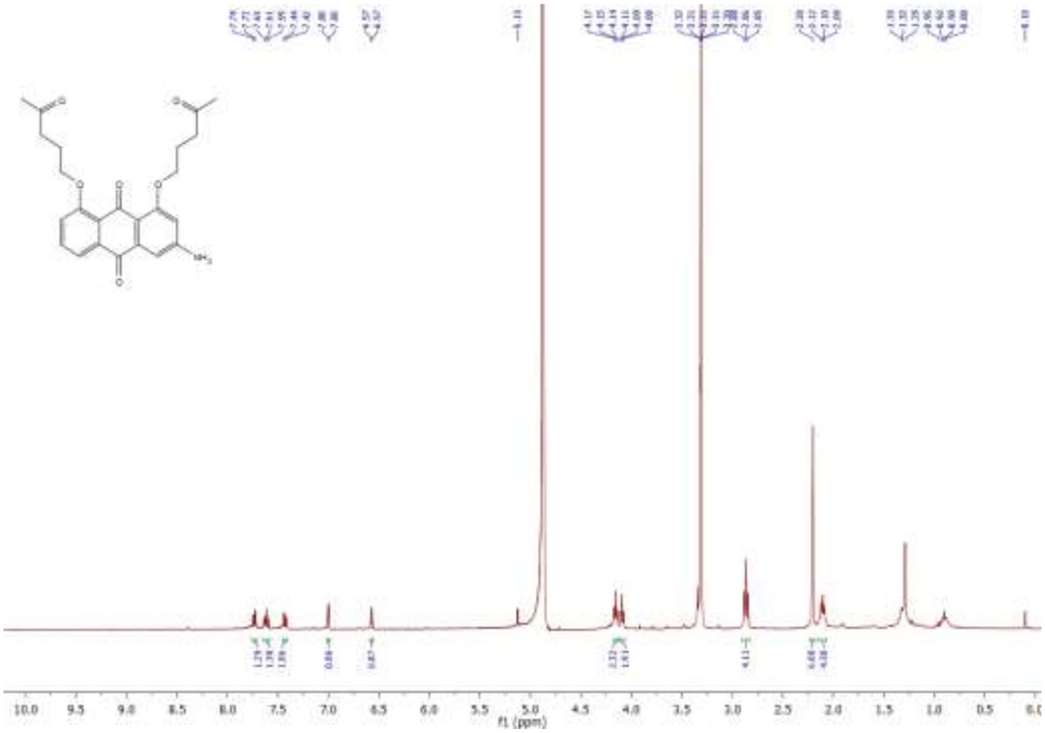

Compound **6**:

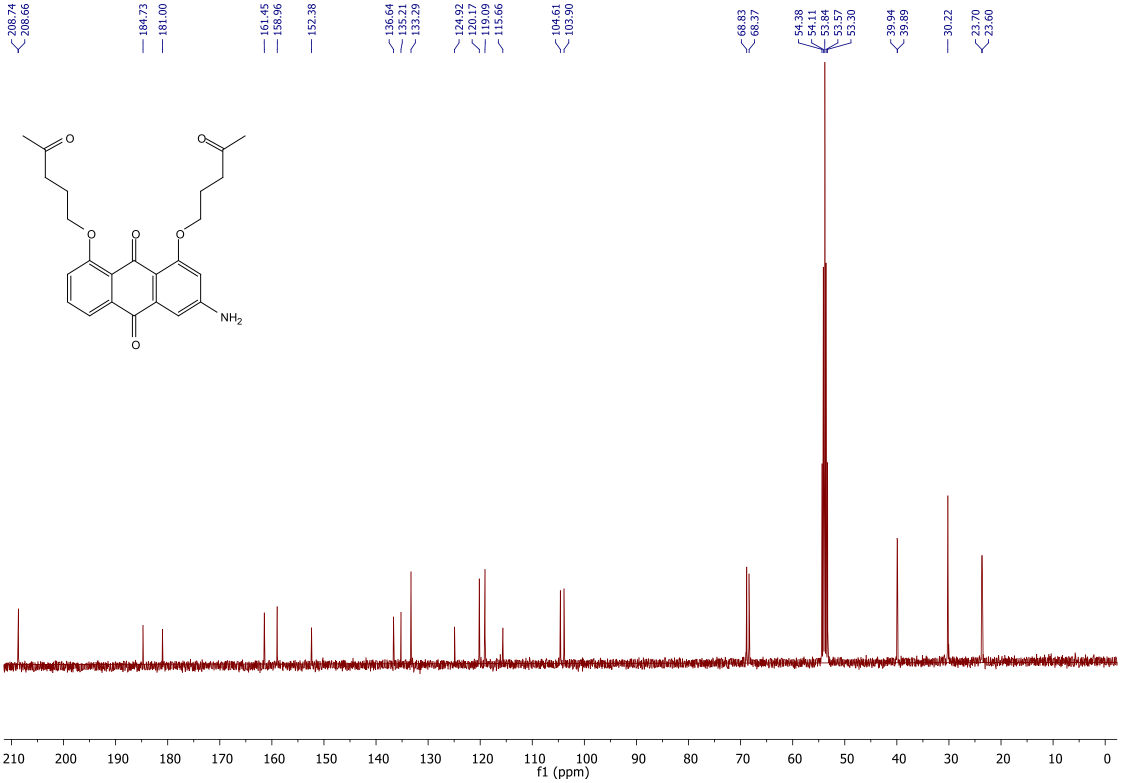

Compound **7**:

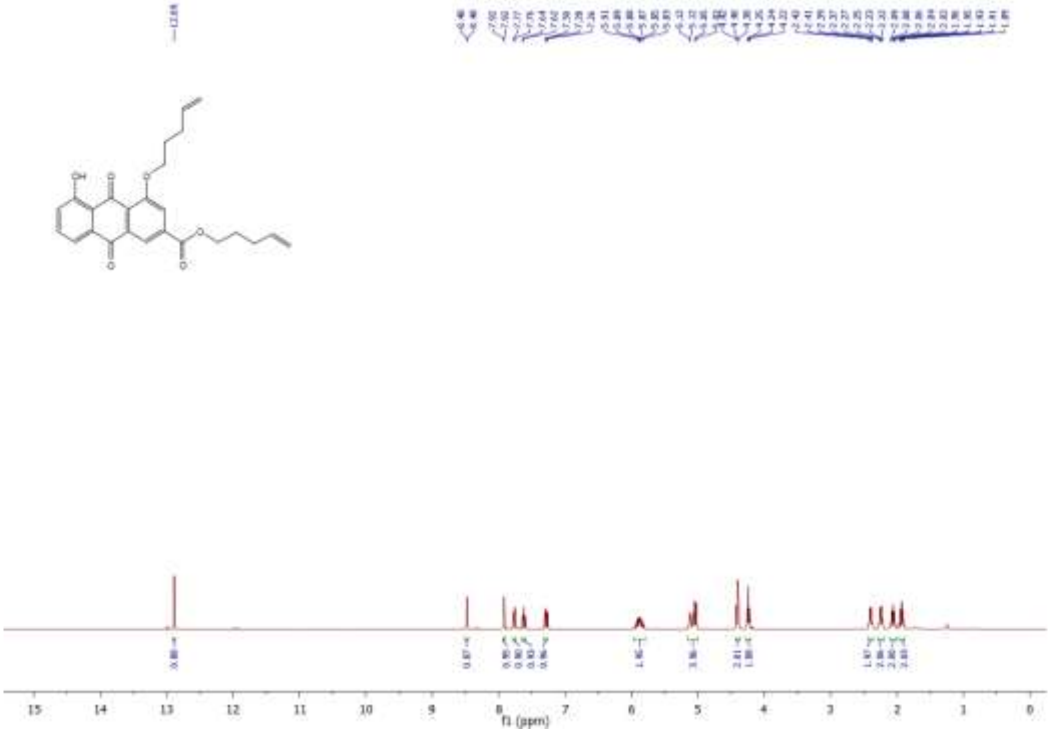

Compound **7**:

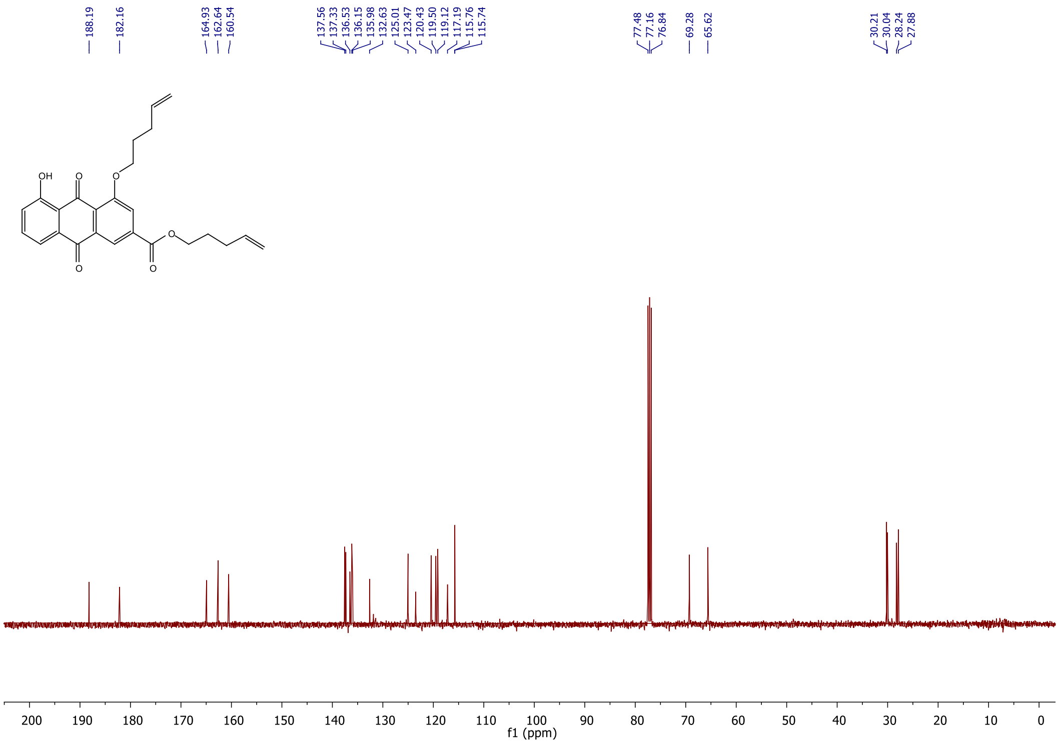

Compound **8**:

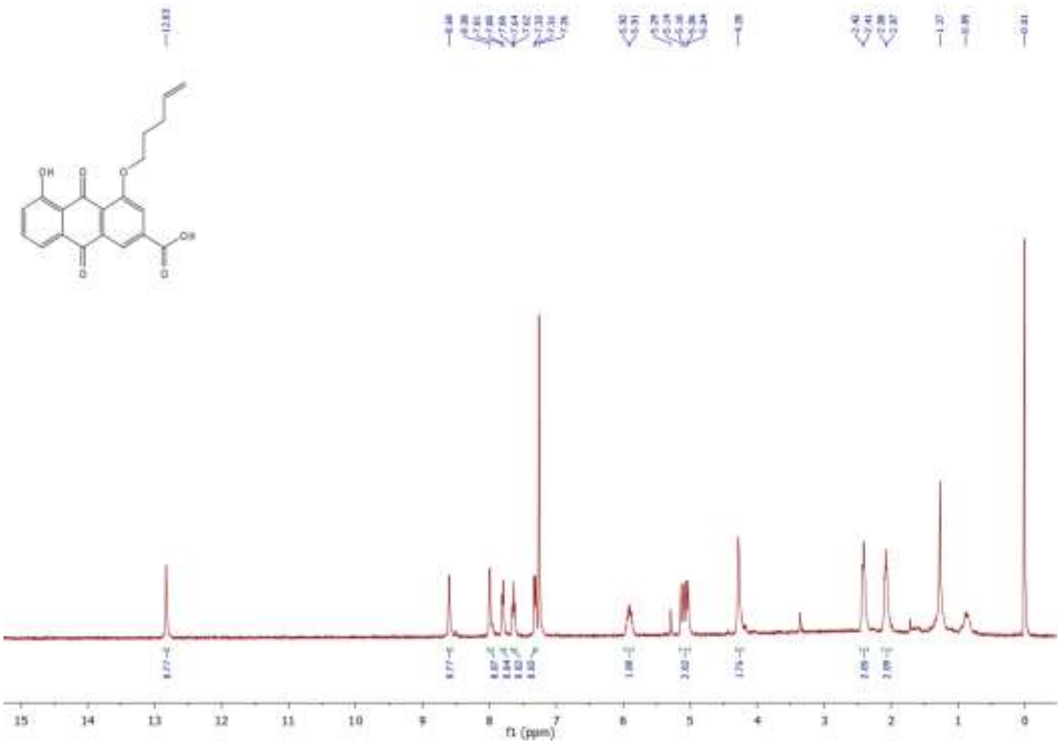

Compound **8**:

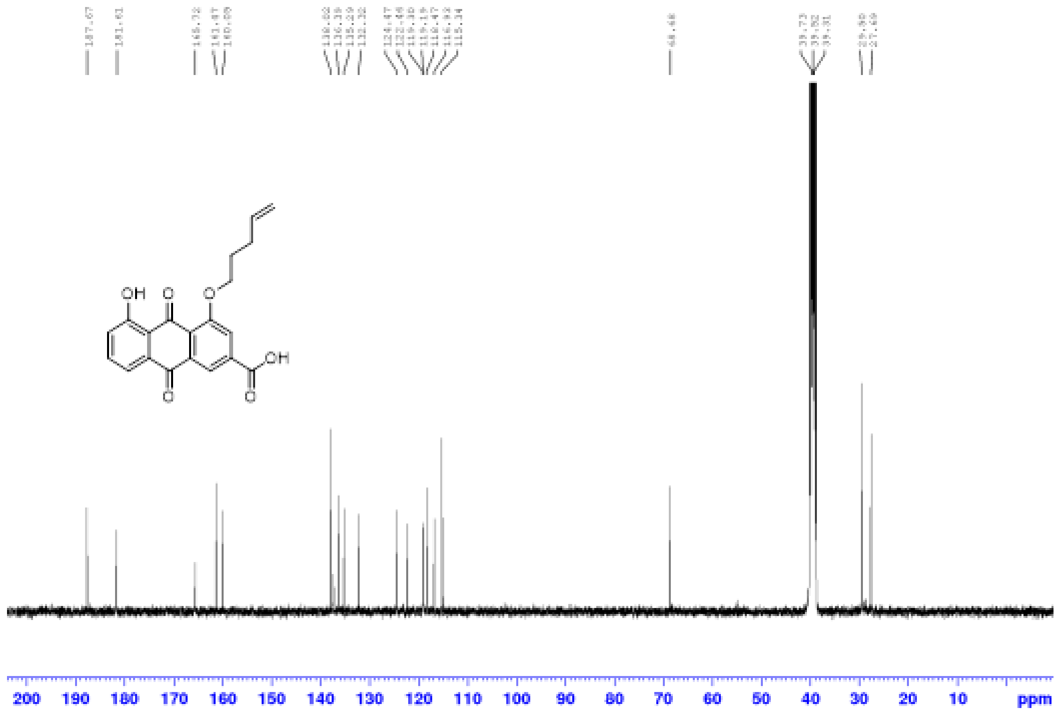

Compound **9**:

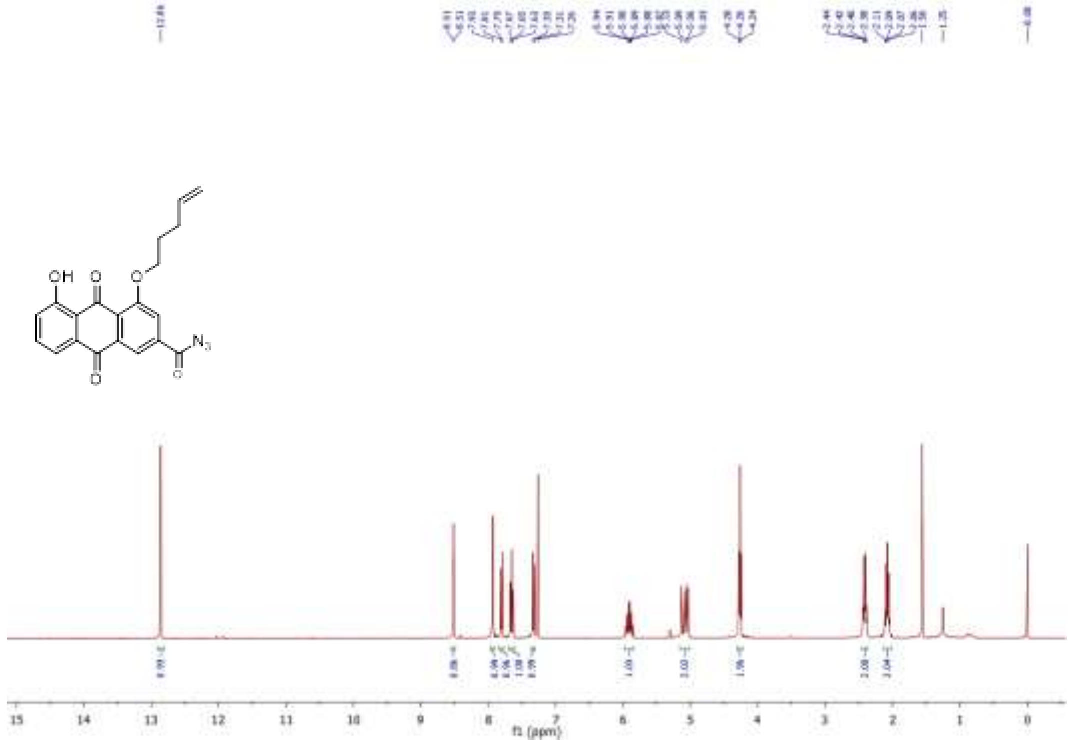

Compound **9**:

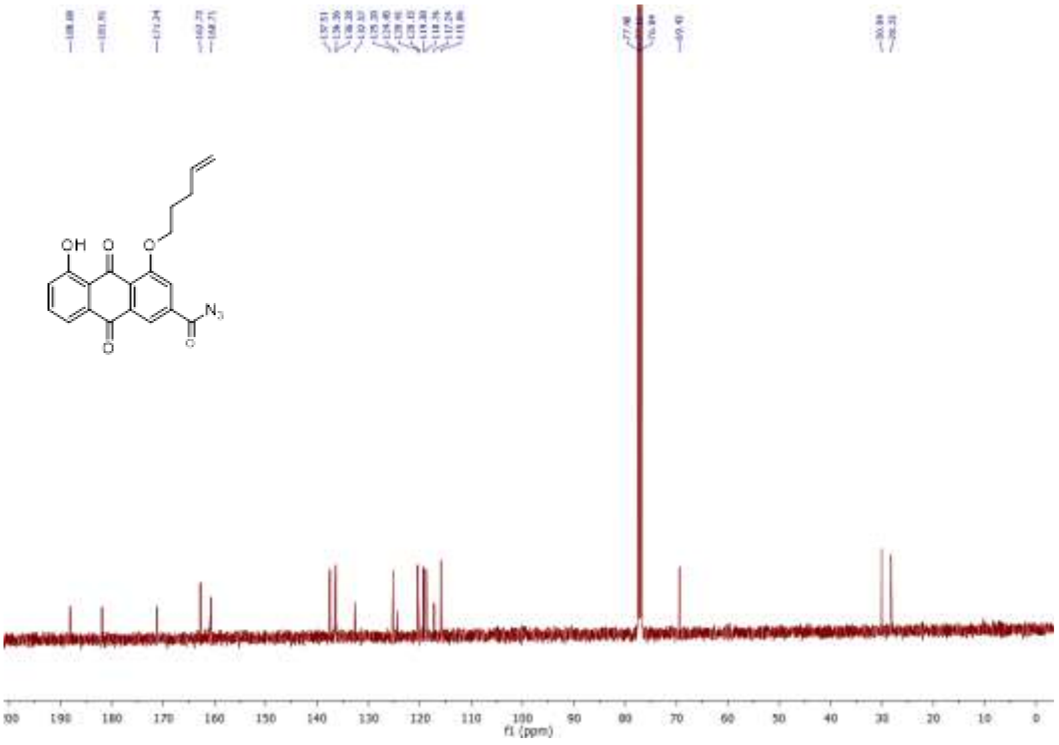

Information Classification: General

Compound **10**:

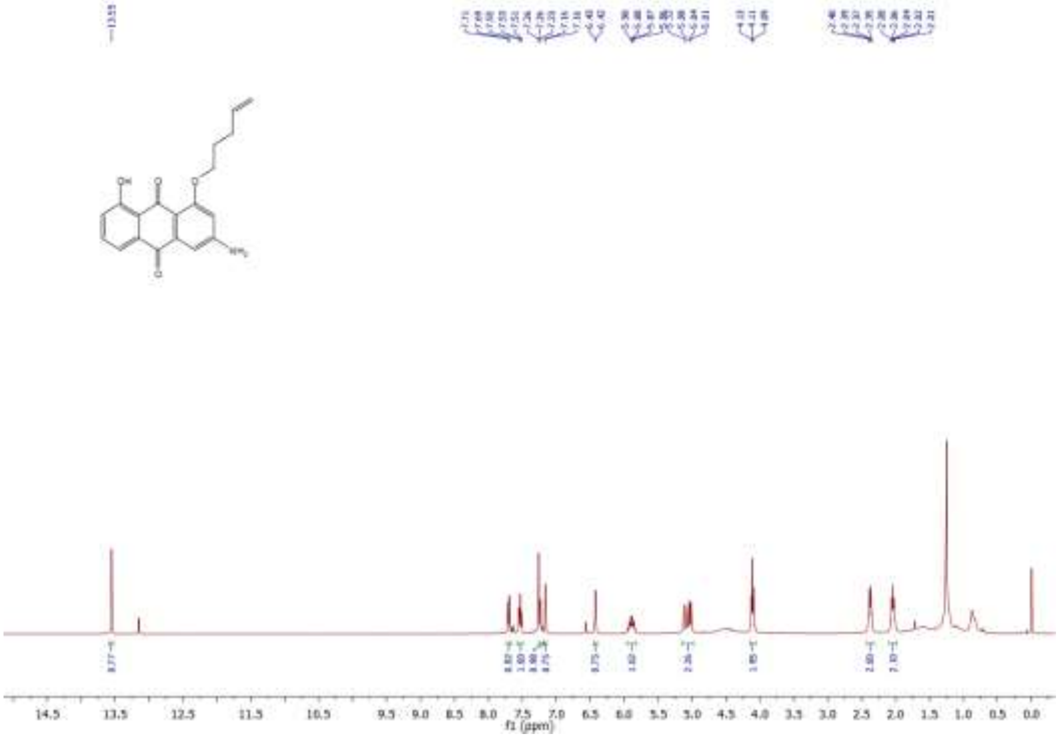

Compound **10**:

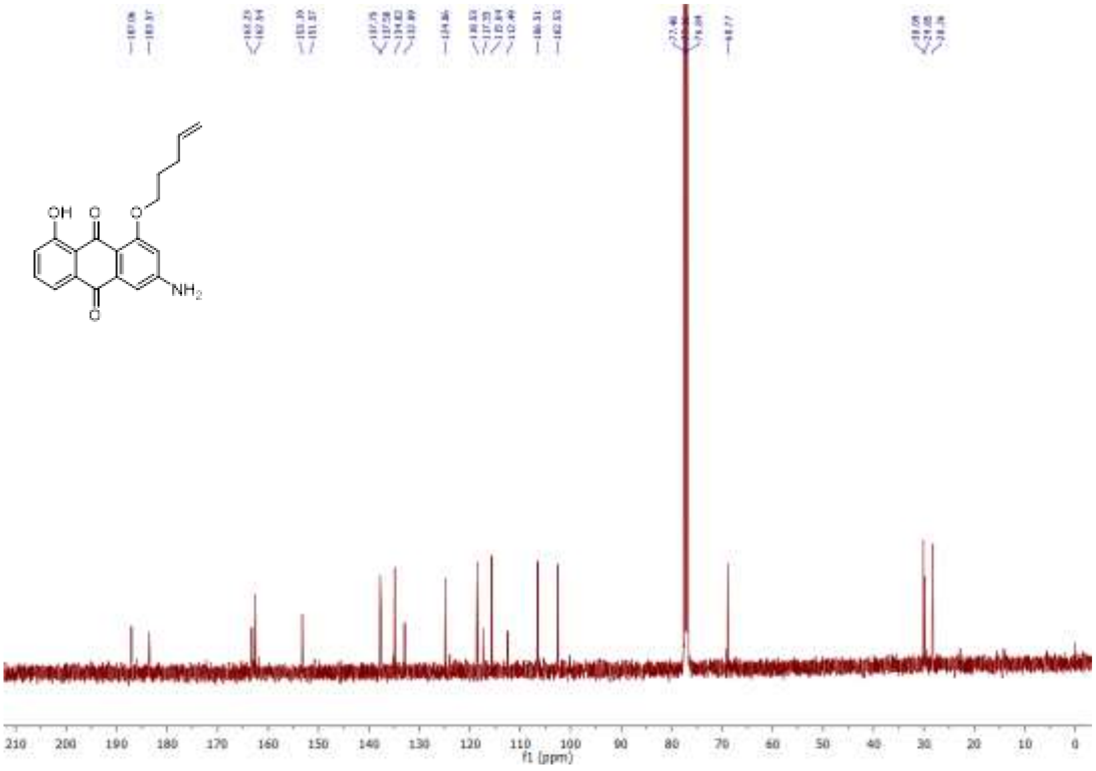

Compound **11**:

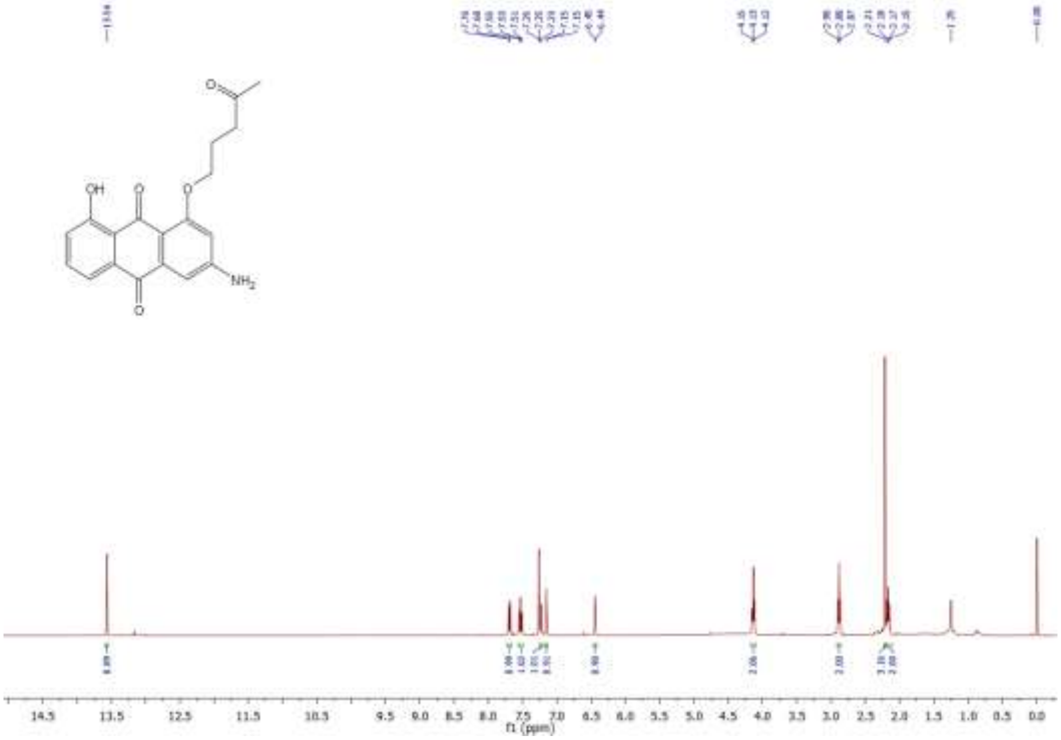

Compound **11**:

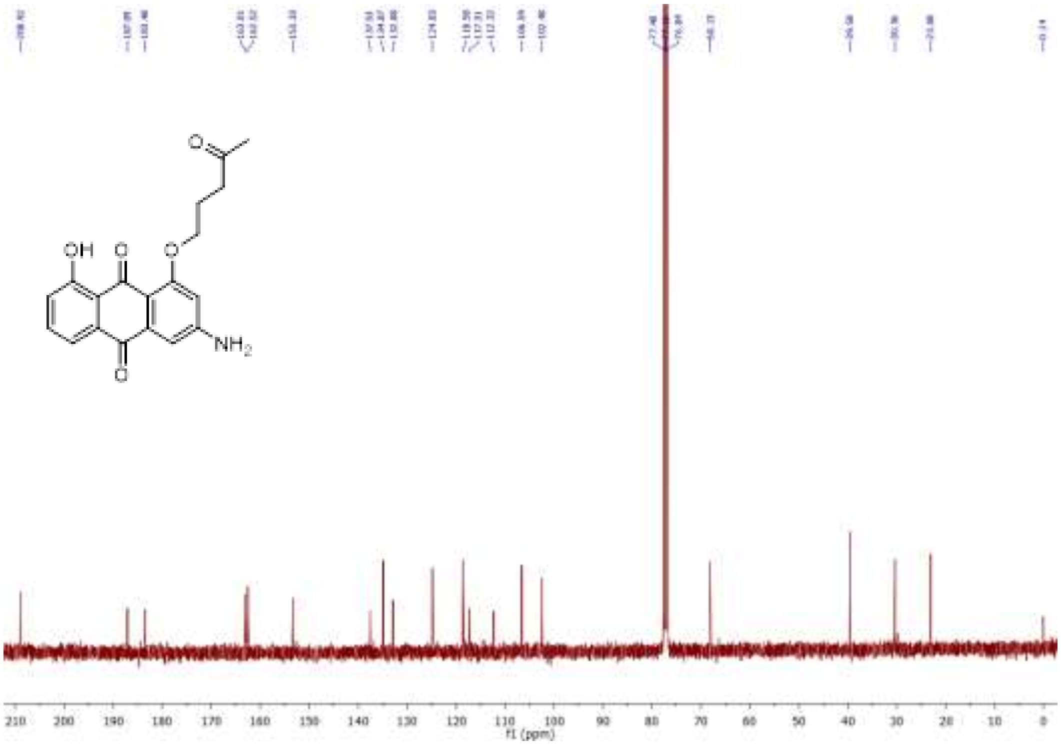

BW-AQ-336:

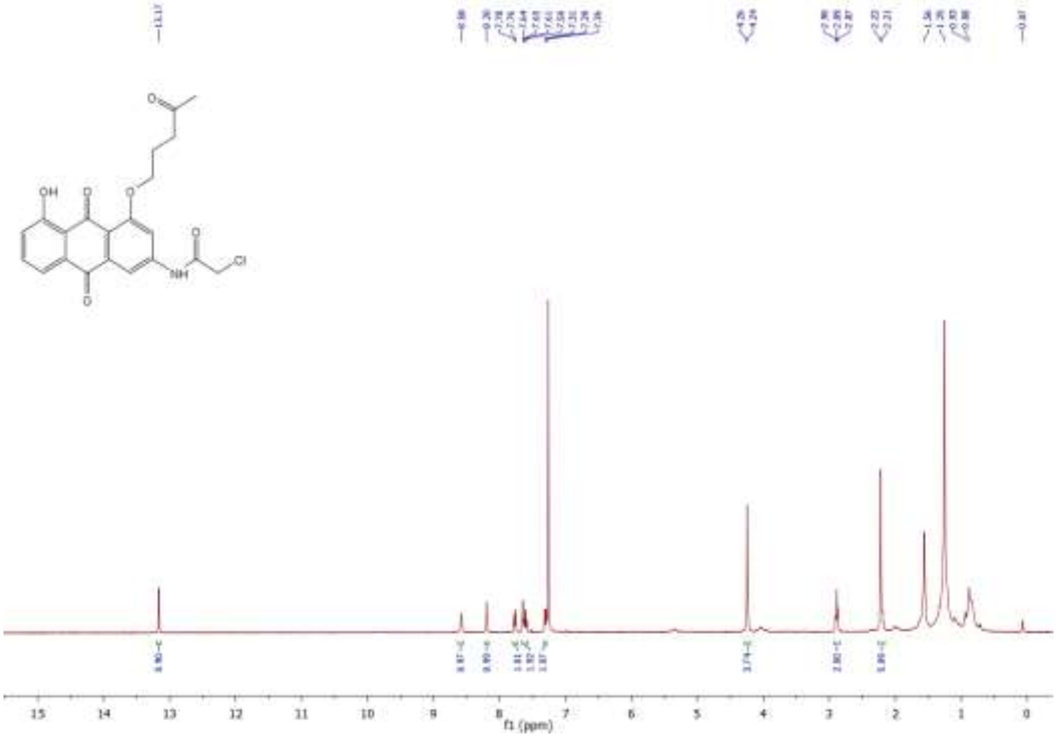

BW-AQ-336:

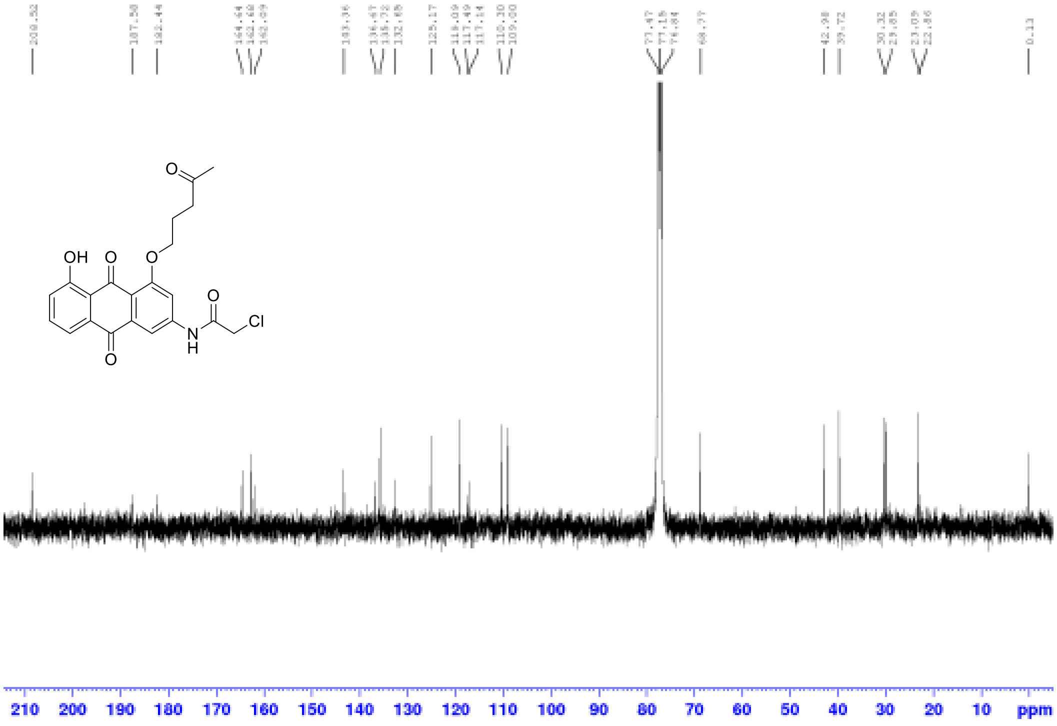

BW-AQ-350:

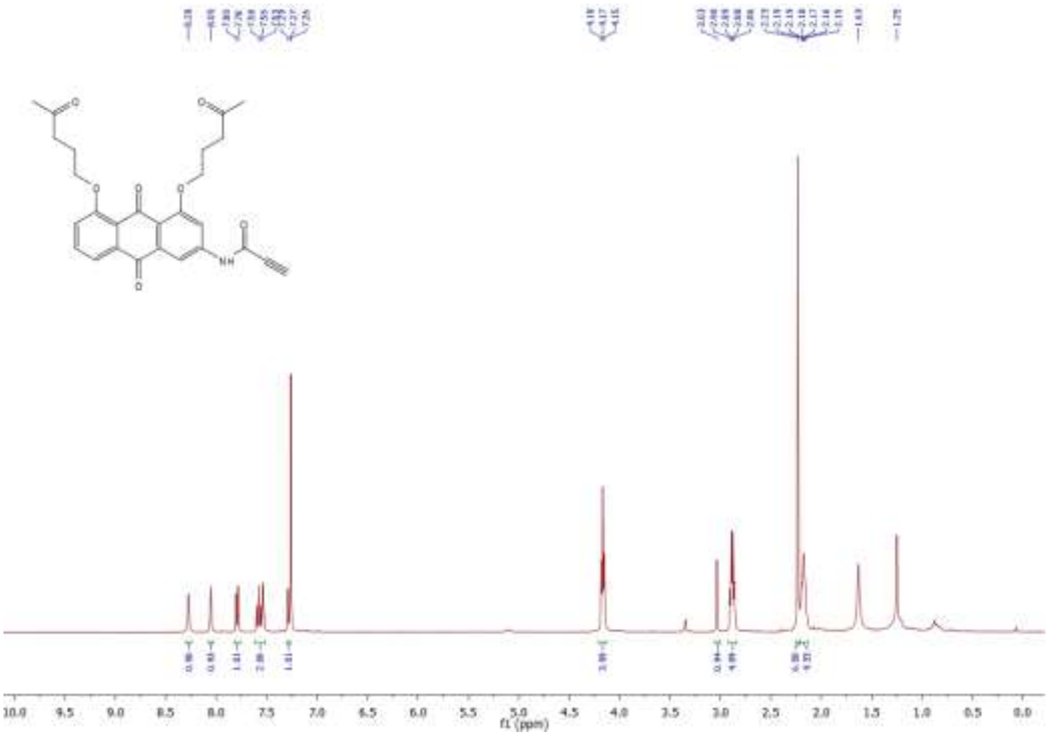

BW-AQ-350:

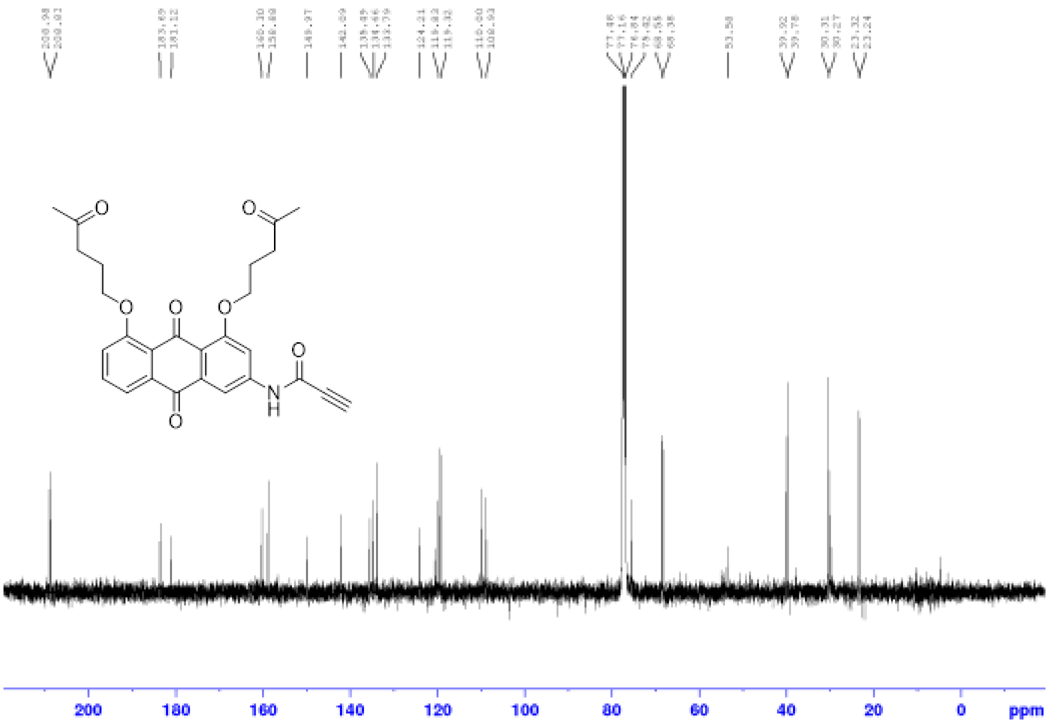

BW-AQ-353

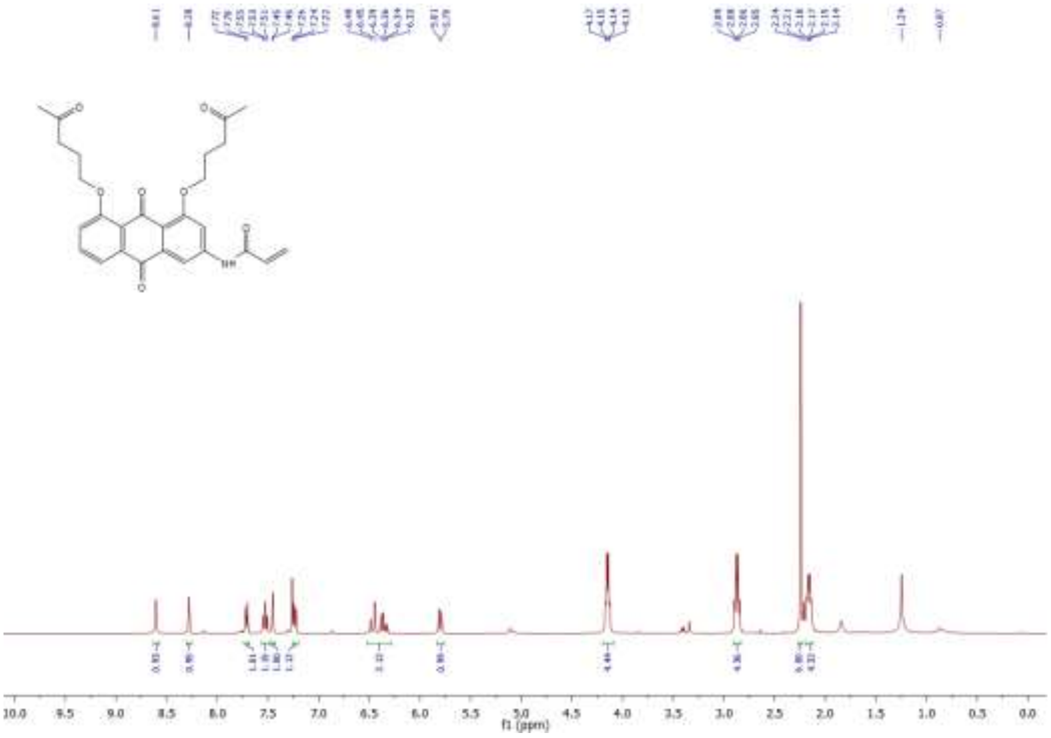

BW-AQ-353:

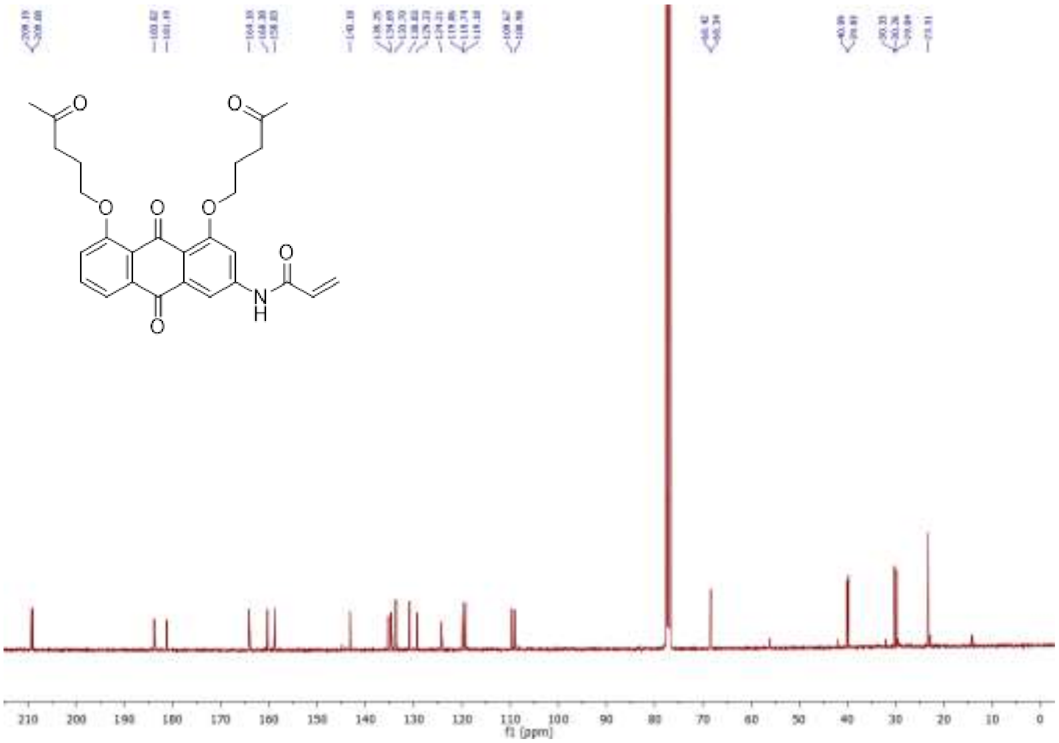

BW-AQ-349:

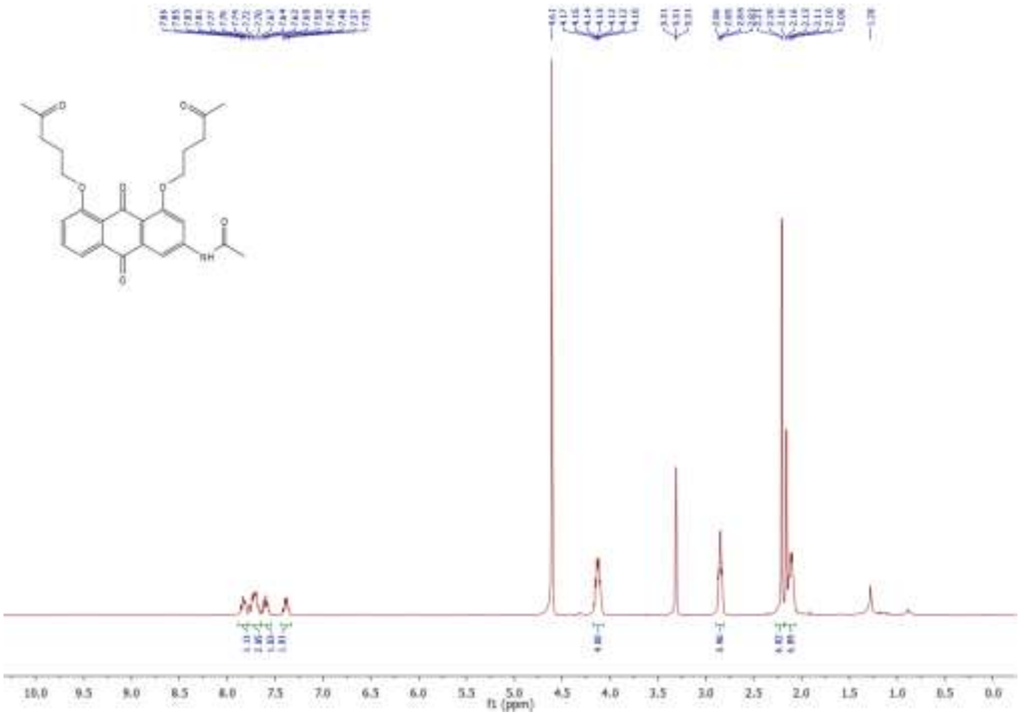

BW-AQ-349:

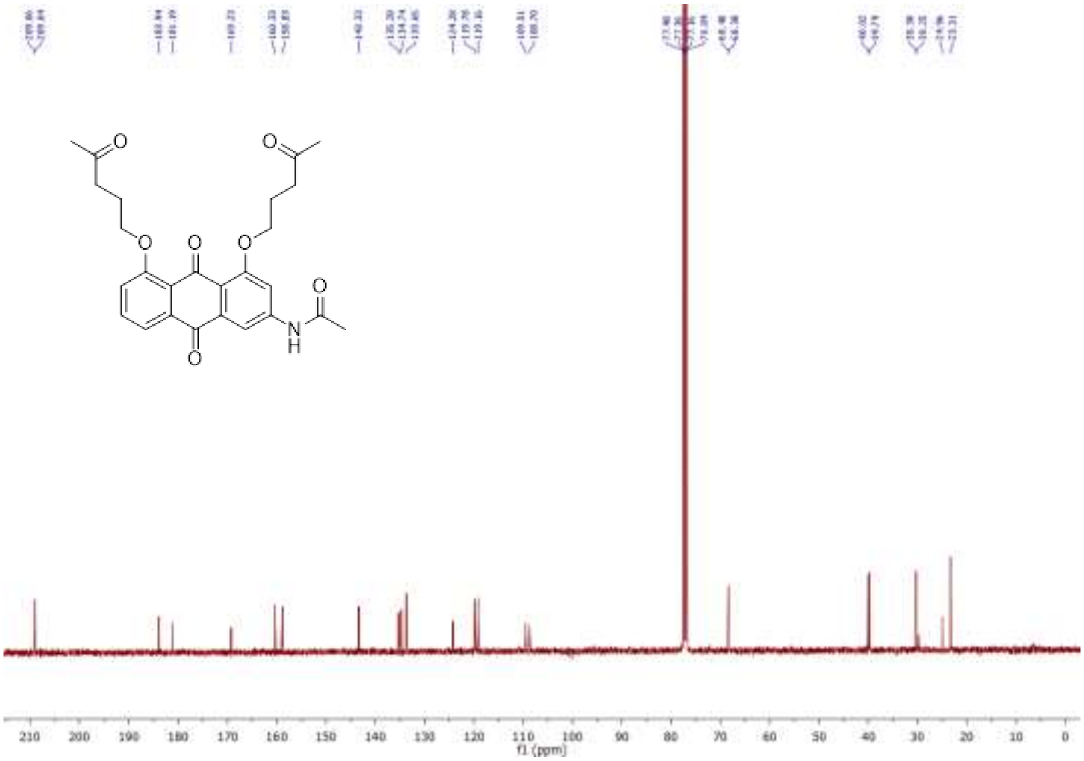

BW-AQ-354:

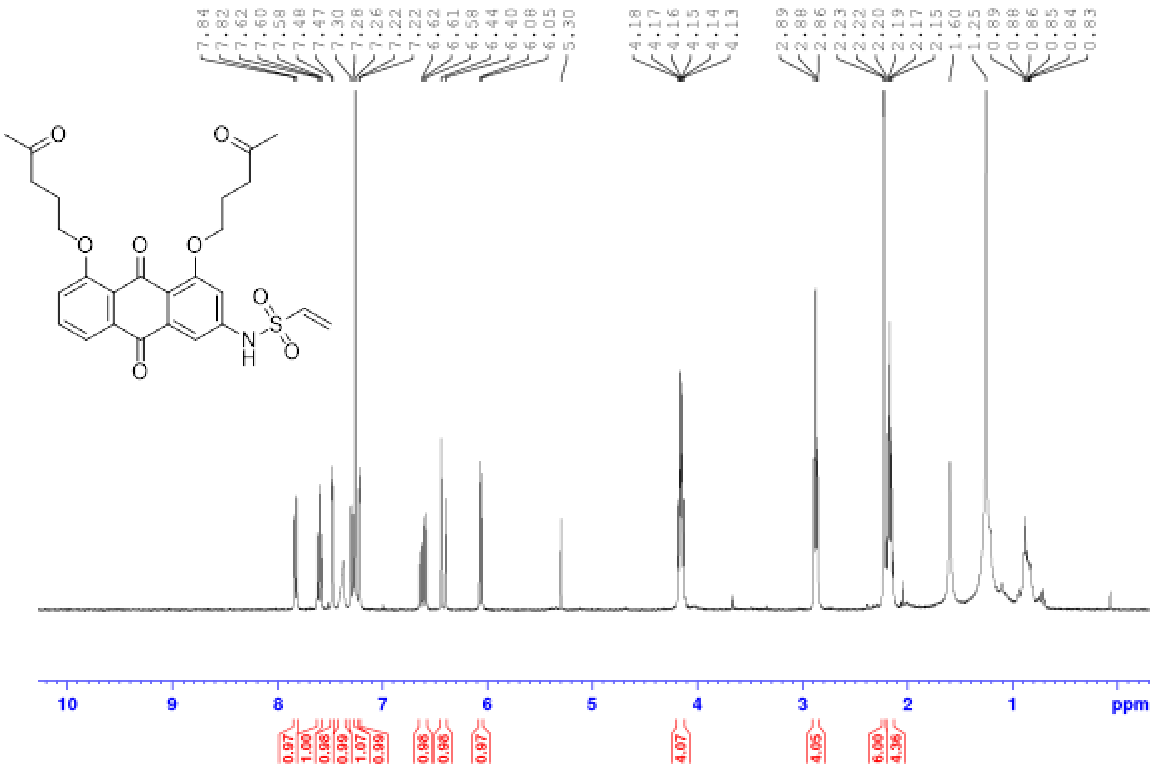

BW-AQ-354:

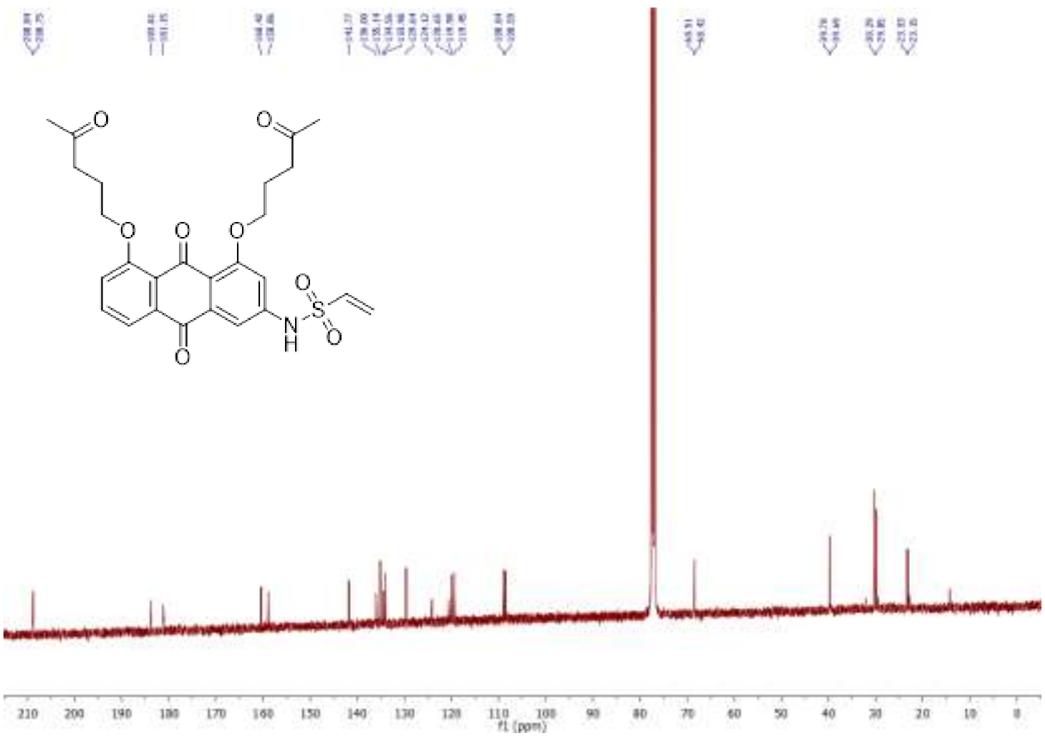

Supplement: Supplemental Material [file IENZ_A_2116699_SM8605.pdf]
